# Supplementary material for: Dehydrogenative reagent-free annulation of alkenes with diols for the synthesis of saturated O-heterocycles
Source: Nat Commun. 2018 Sep 3;9:3551. doi: 10.1038/s41467-018-06020-8 (PMC6120897; doi:10.1038/s41467-018-06020-8)
Supplement: Supplementary file 1 — Supplementary Information [file 41467_2018_6020_MOESM1_ESM.pdf]

Dehydrogenative Reagent-Free Annulation of Alkenes with Diols for the Synthesis of Saturated O-Heterocycles

Cai et al.

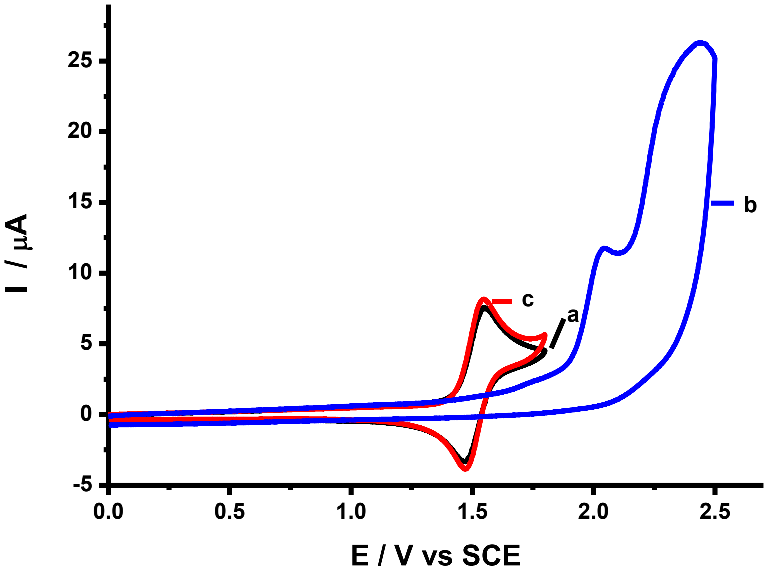

Supplementary Fig. 1. Cyclic voltammograms. a) **3** (2.6 mM). b) **4** (1.3 mM). c) **4** (1.3 mM) +**3** (2.6 mM).

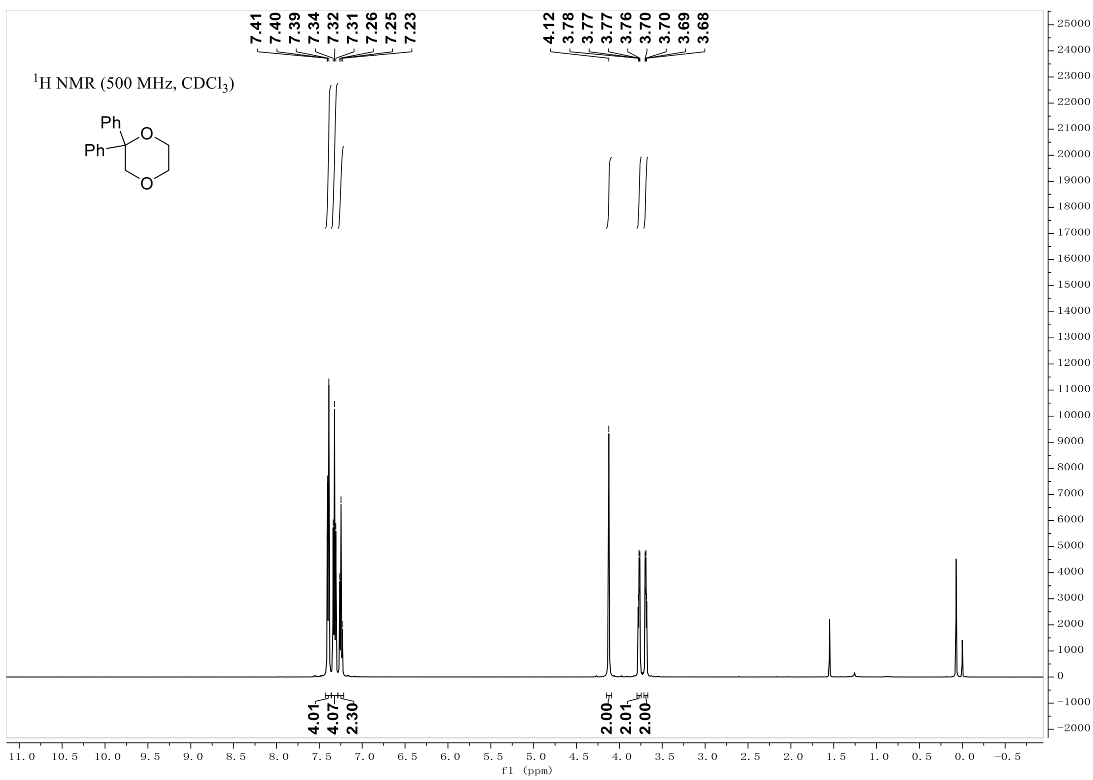

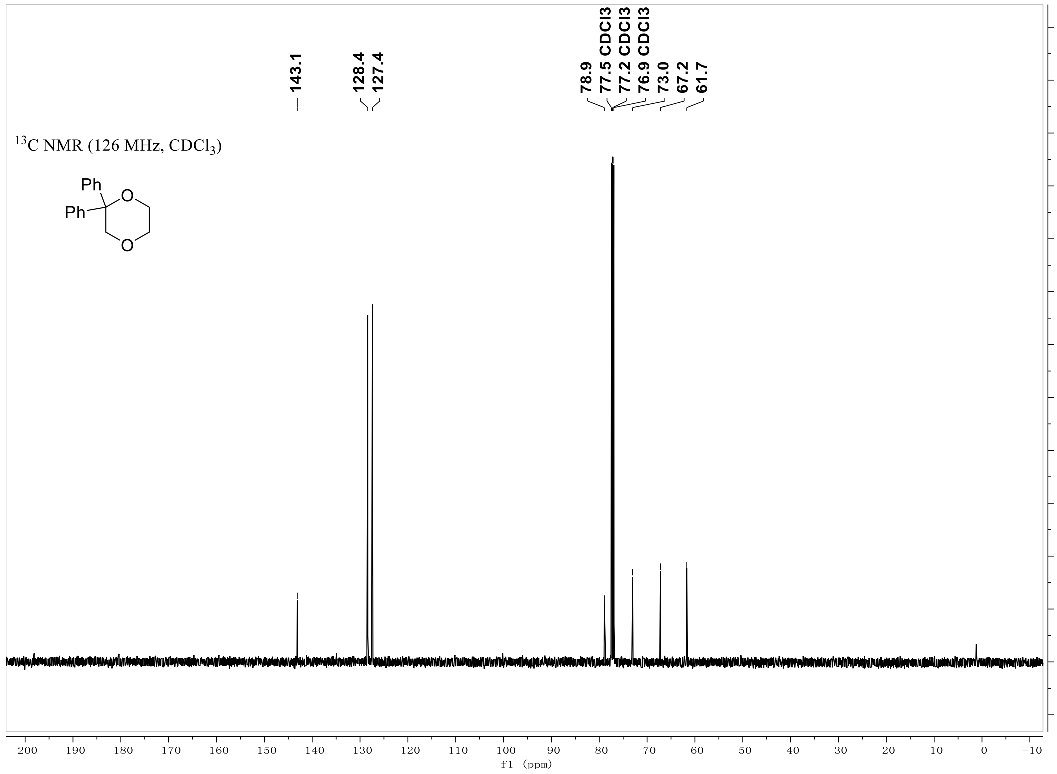

Supplementary Fig. 2. ^{1}H NMR and ^{13}C NMR spectra for compound 4

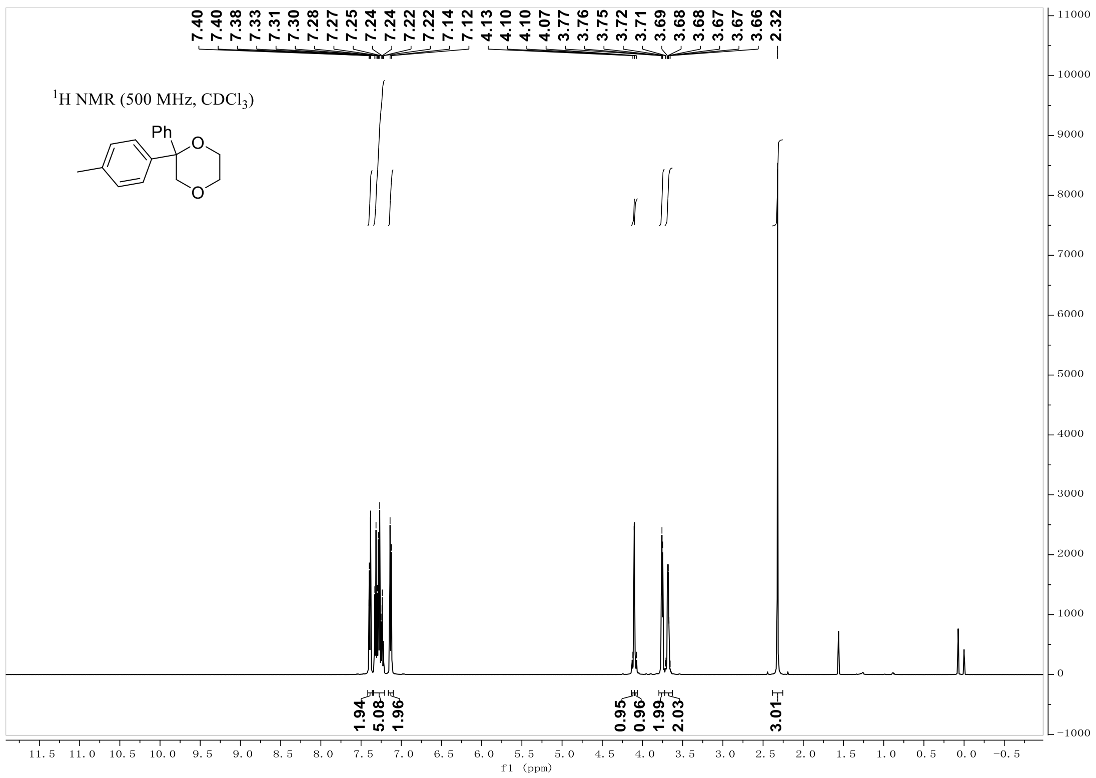

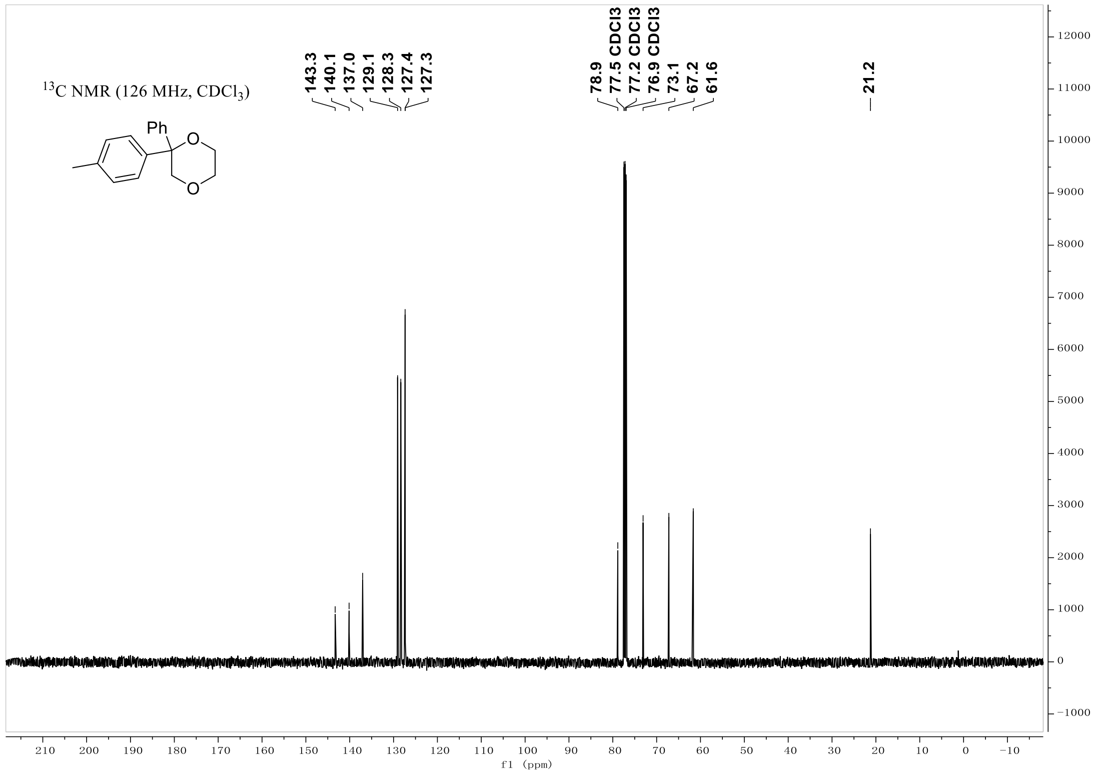

Supplementary Fig. 3. ^{1}H NMR and ^{13}C NMR spectra for compound 7

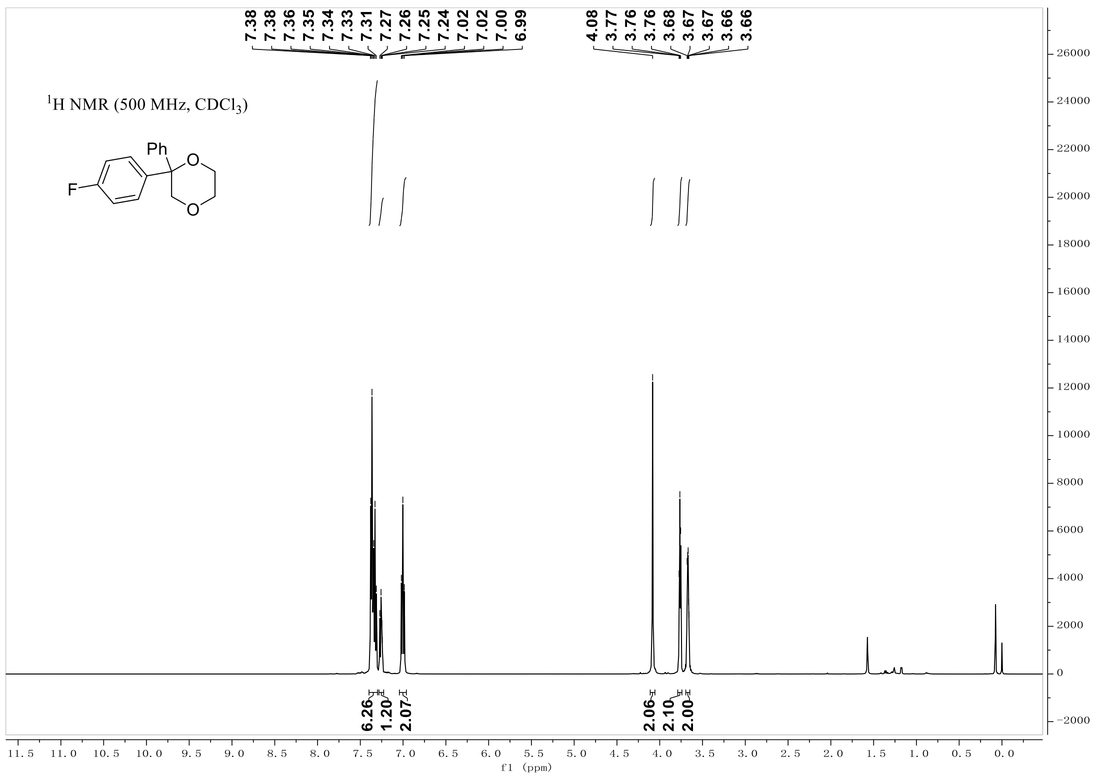

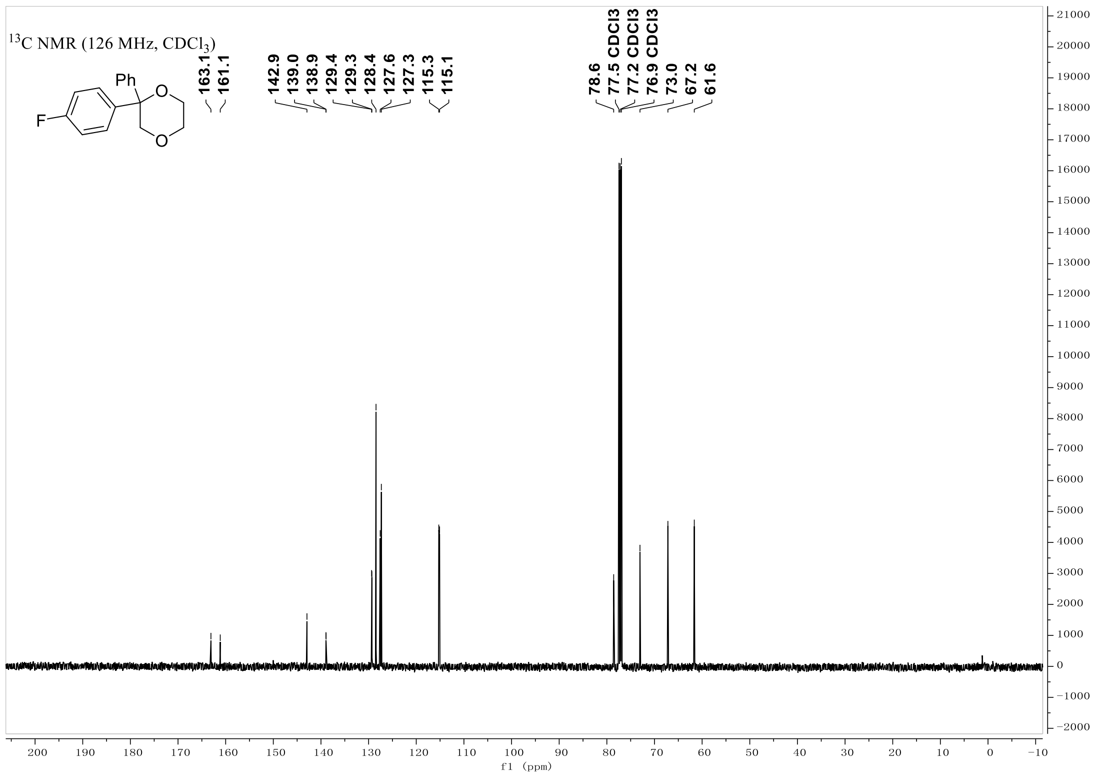

Supplementary Fig. 4. ^{1}H NMR and ^{13}C NMR spectra for compound 8

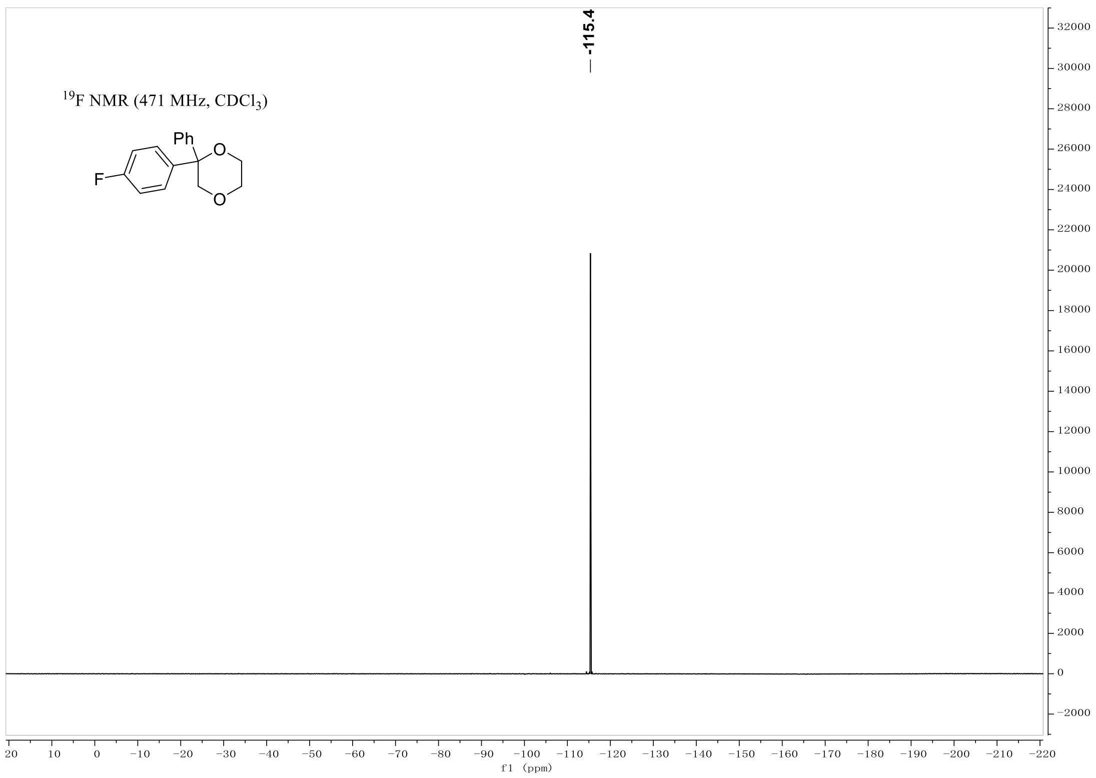

Supplementary Fig. 5. ^{19}F NMR spectra for compound 8

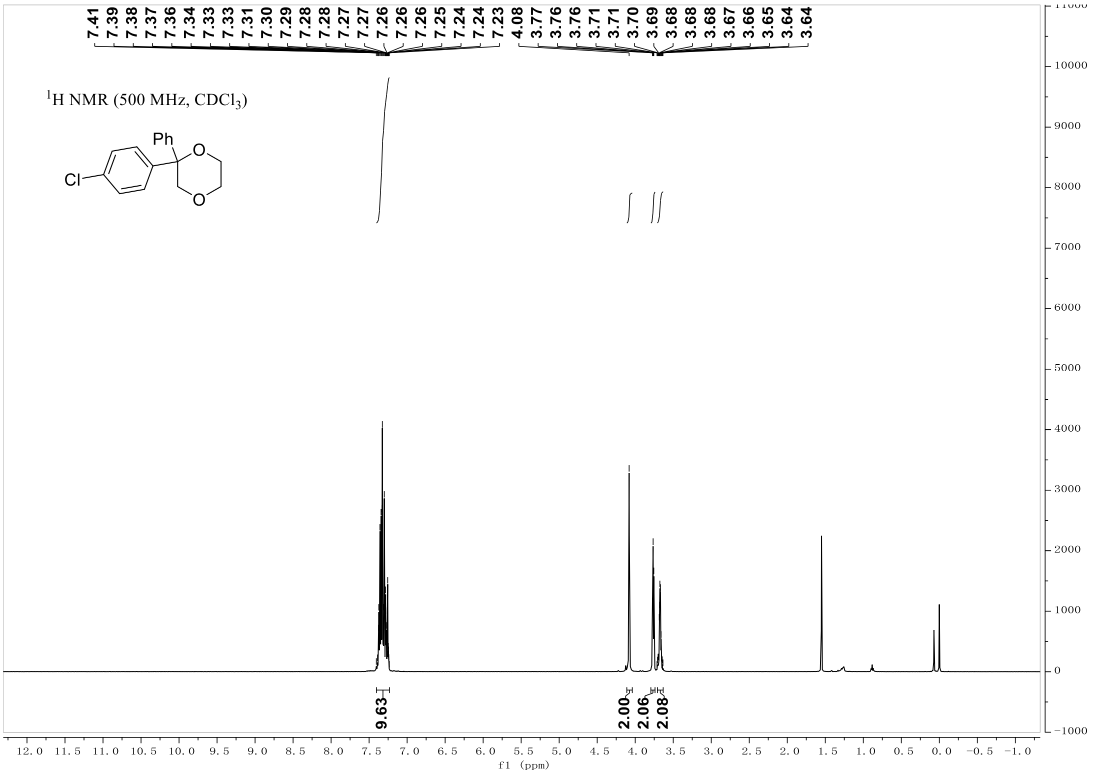

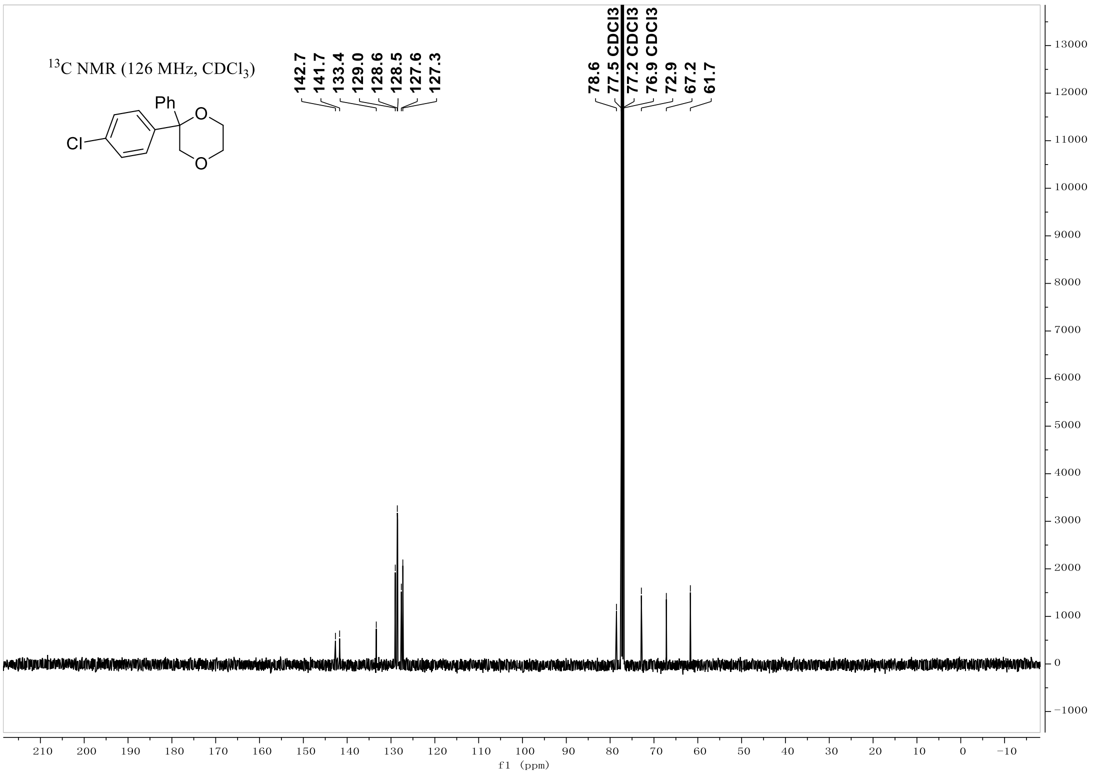

Supplementary Fig. 6. ^{1}H NMR and ^{13}C NMR spectra for compound 9

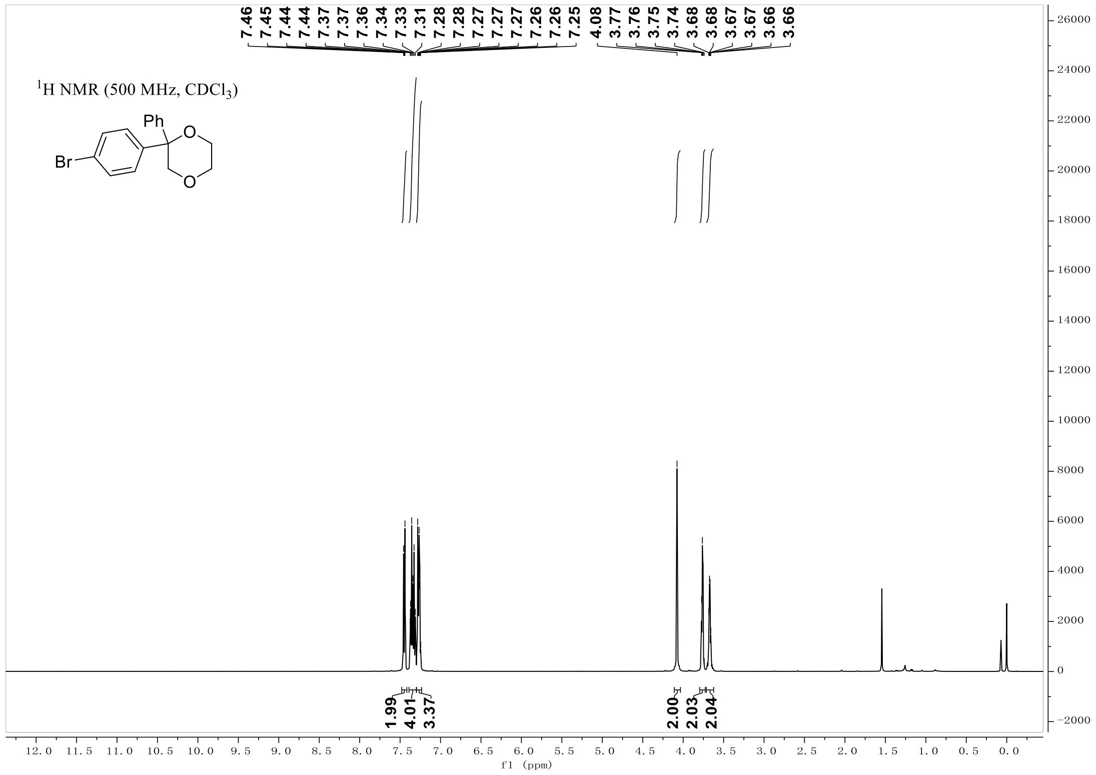

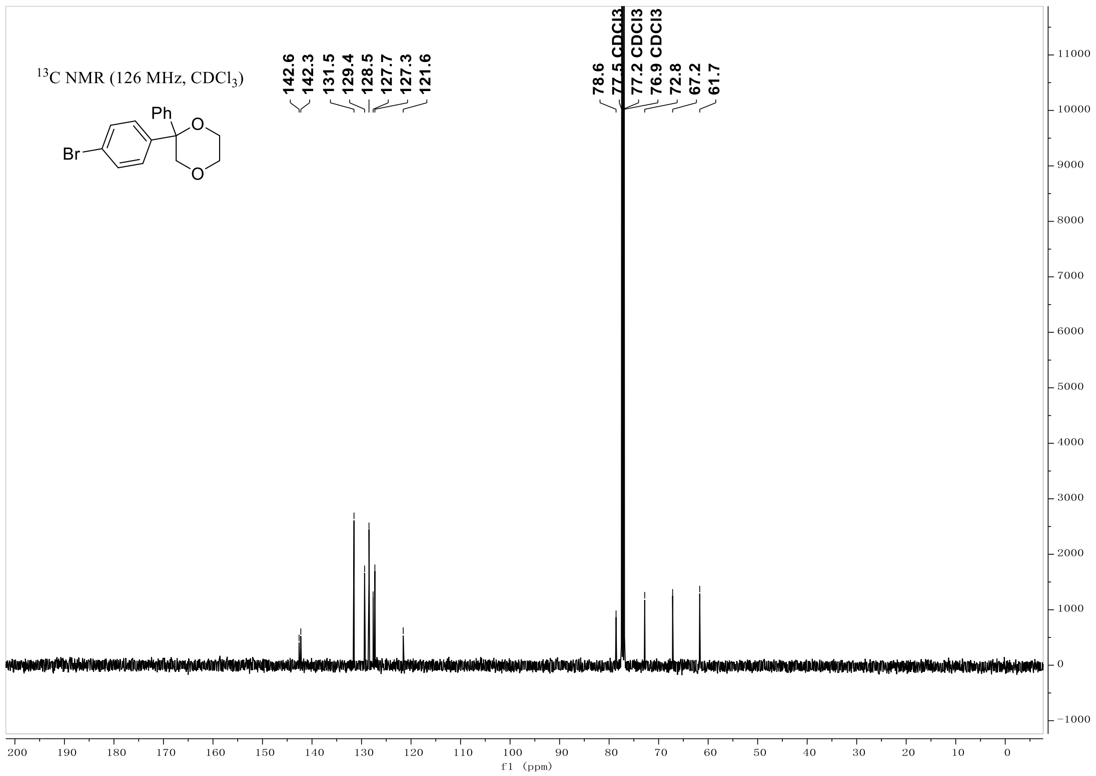

Supplementary Fig. 7. ^{1}H NMR and ^{13}C NMR spectra for compound 10

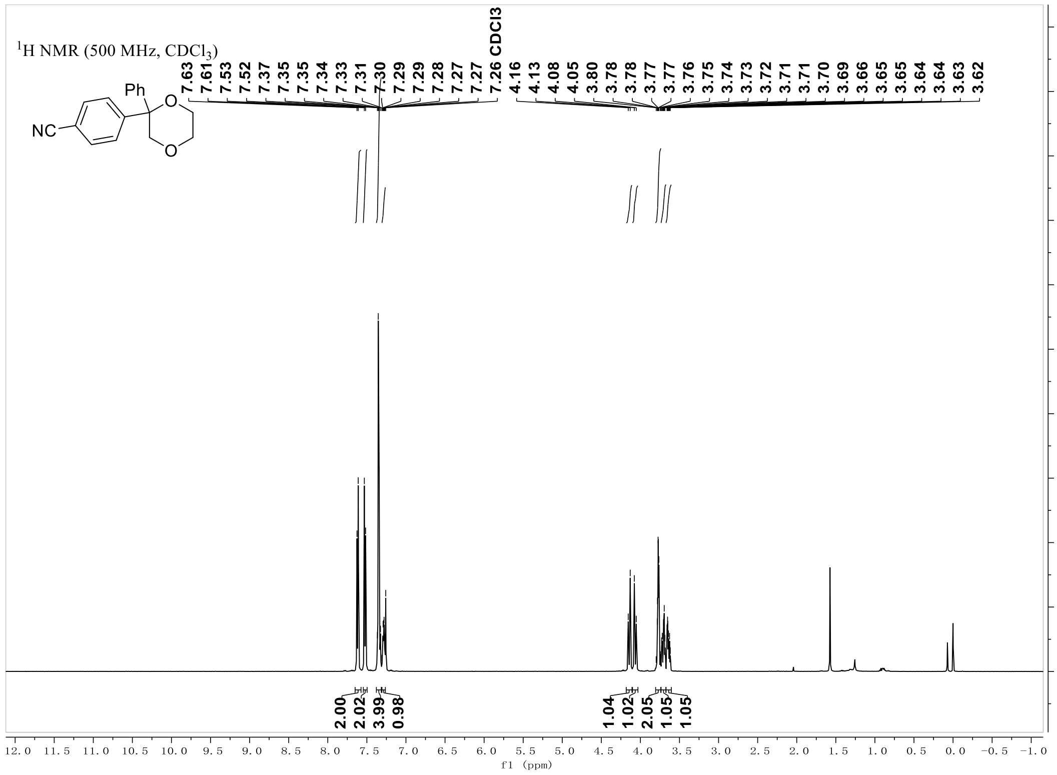

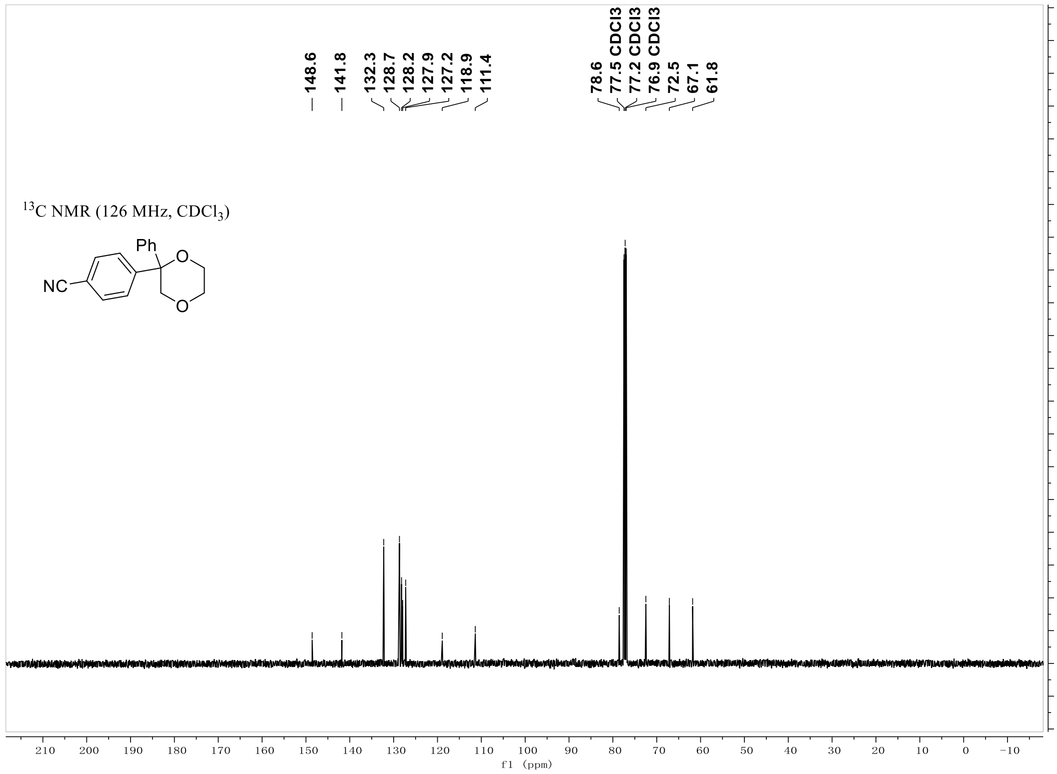

Supplementary Fig. 8. ^{1}H NMR and ^{13}C NMR spectra for compound 11

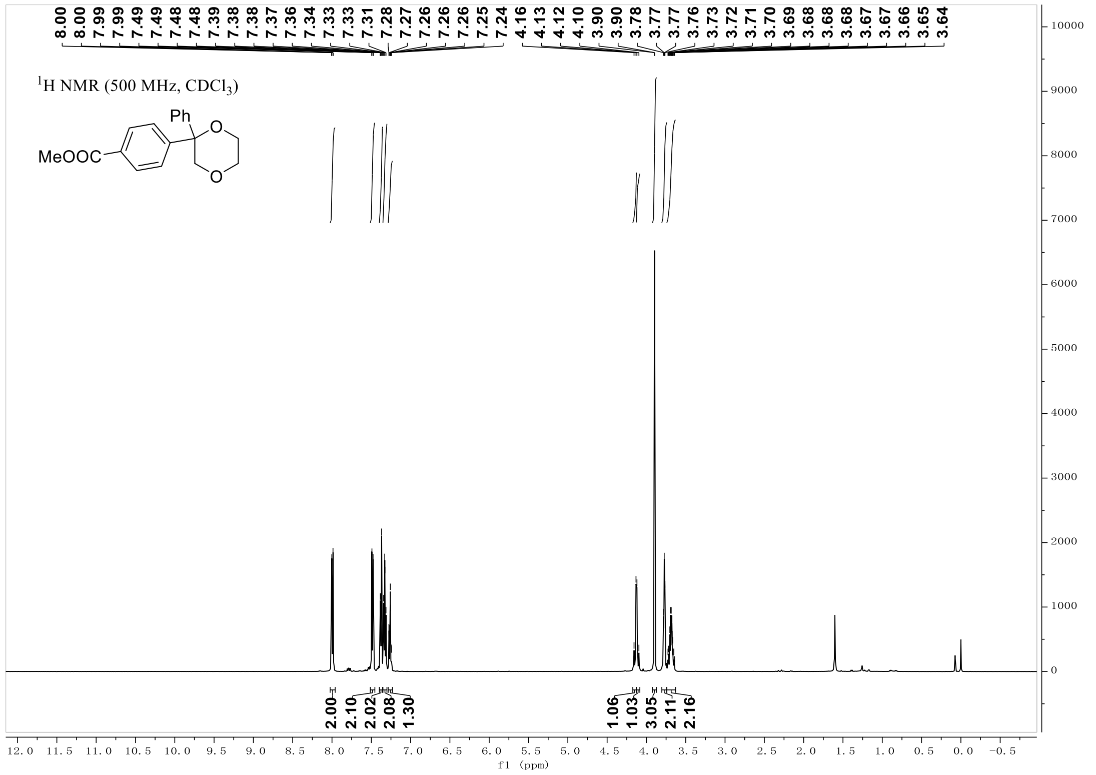

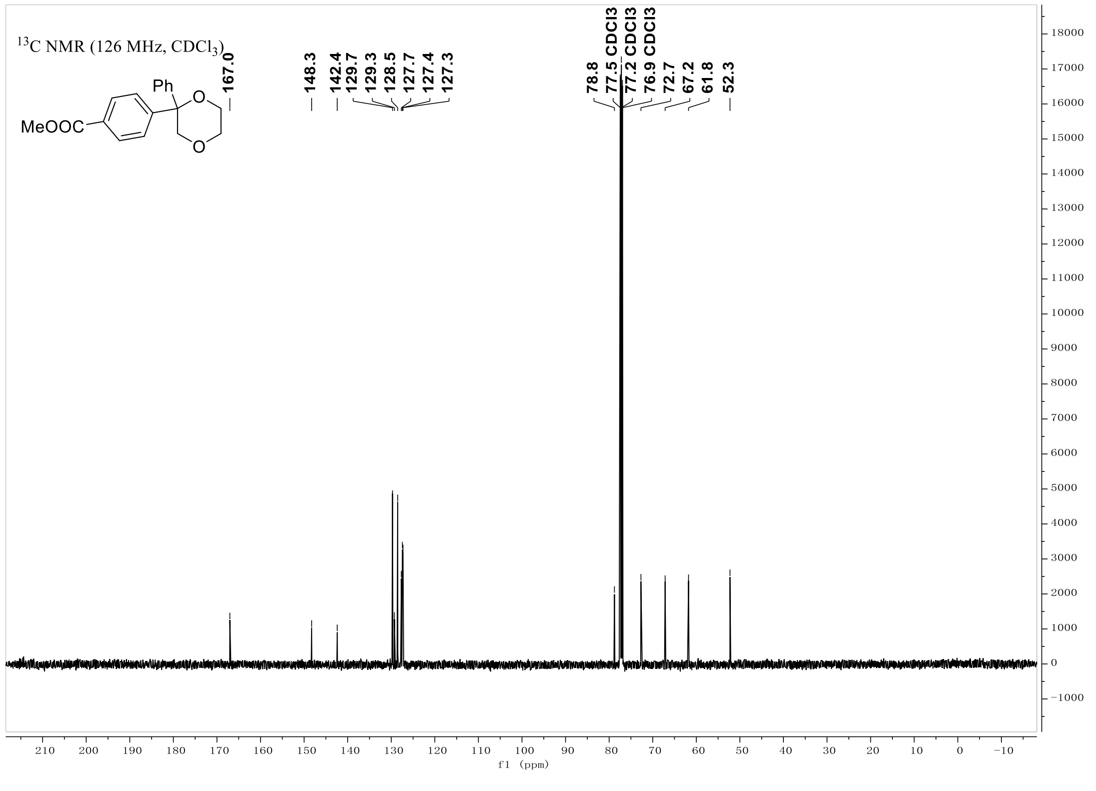

Supplementary Fig. 9. ^1H NMR and ^{13}C NMR spectra for compound 12

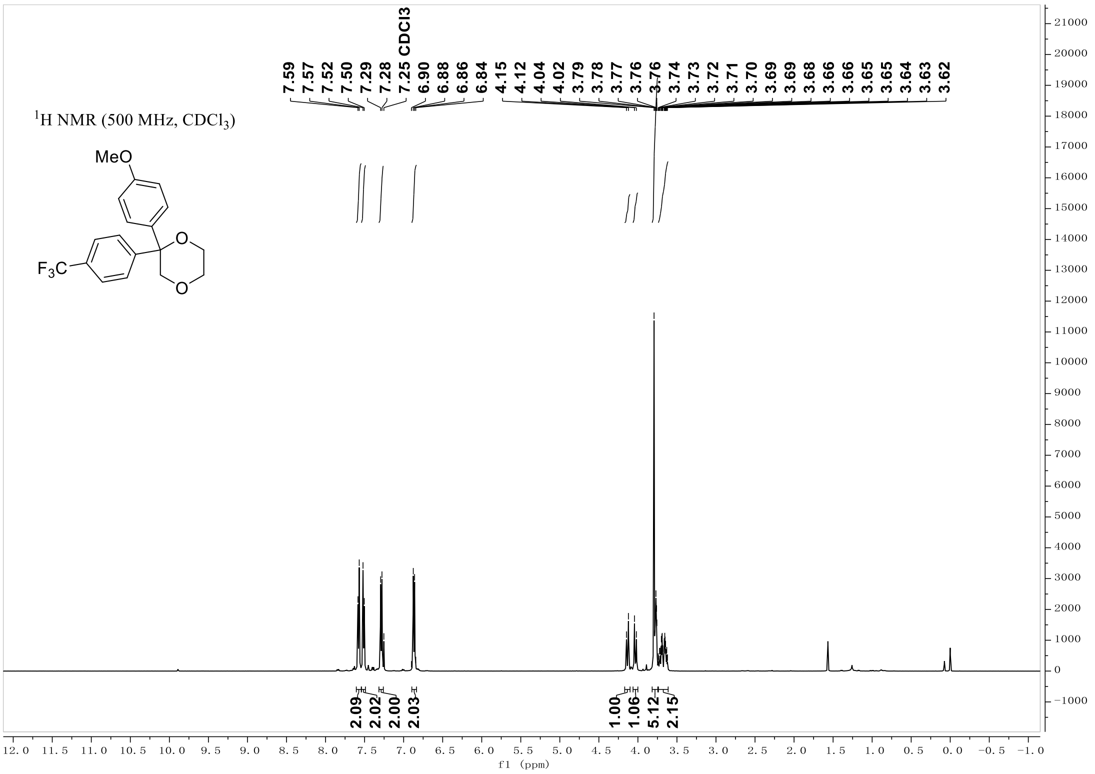

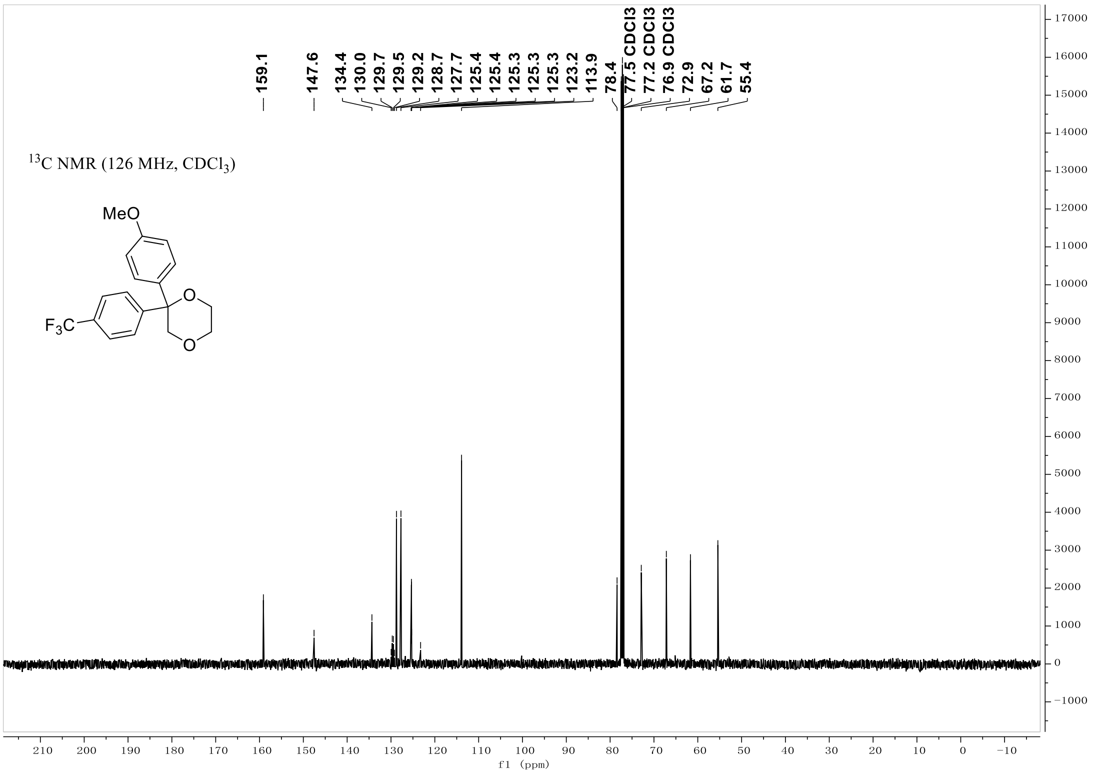

Supplementary Fig. 10. ^{1}H NMR and ^{13}C NMR spectra for compound 13

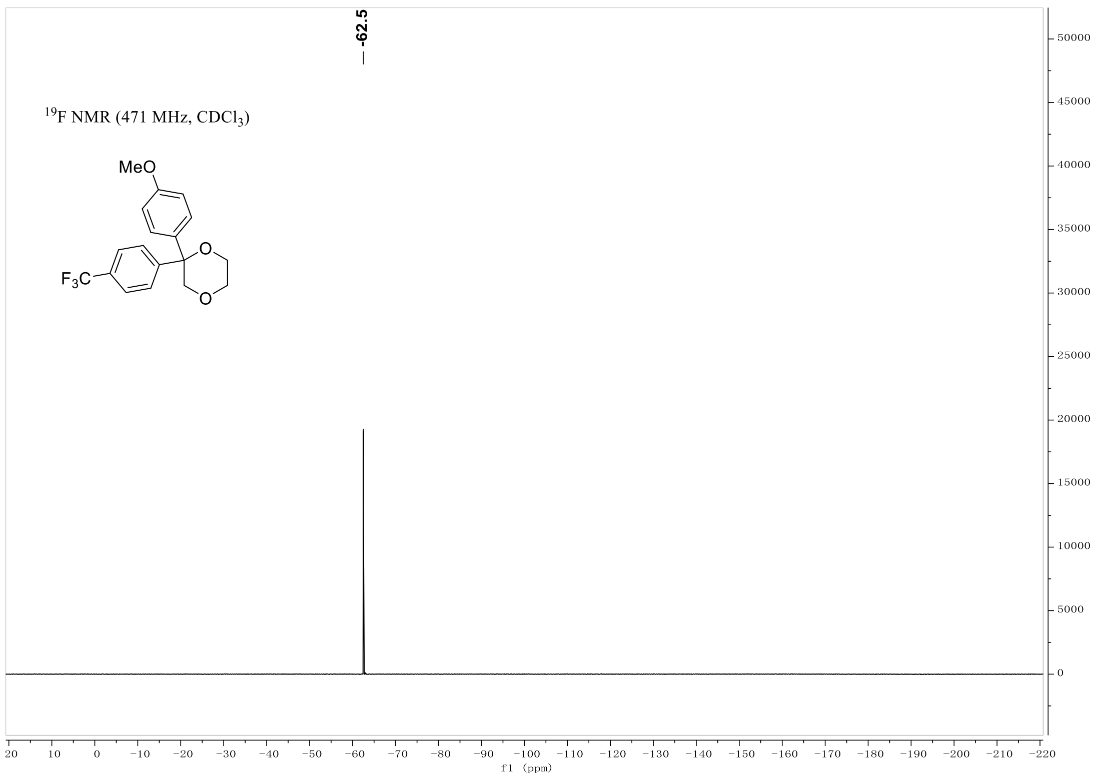

Supplementary Fig. 11. ^{19}F NMR spectra for compound 13

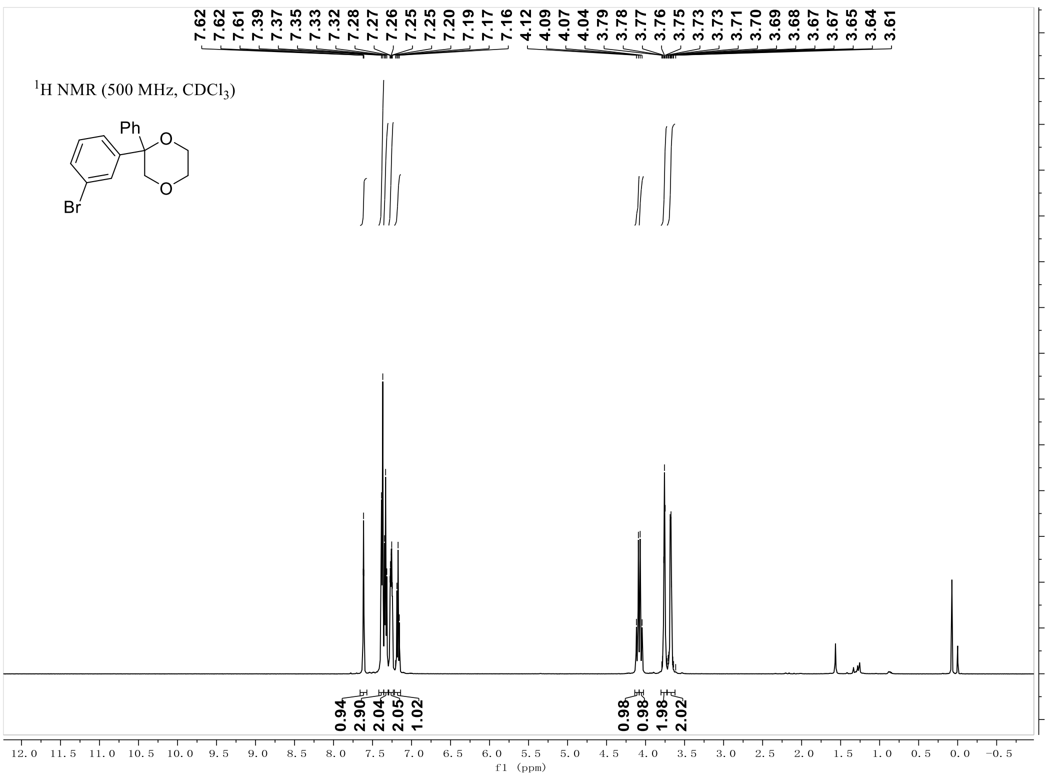

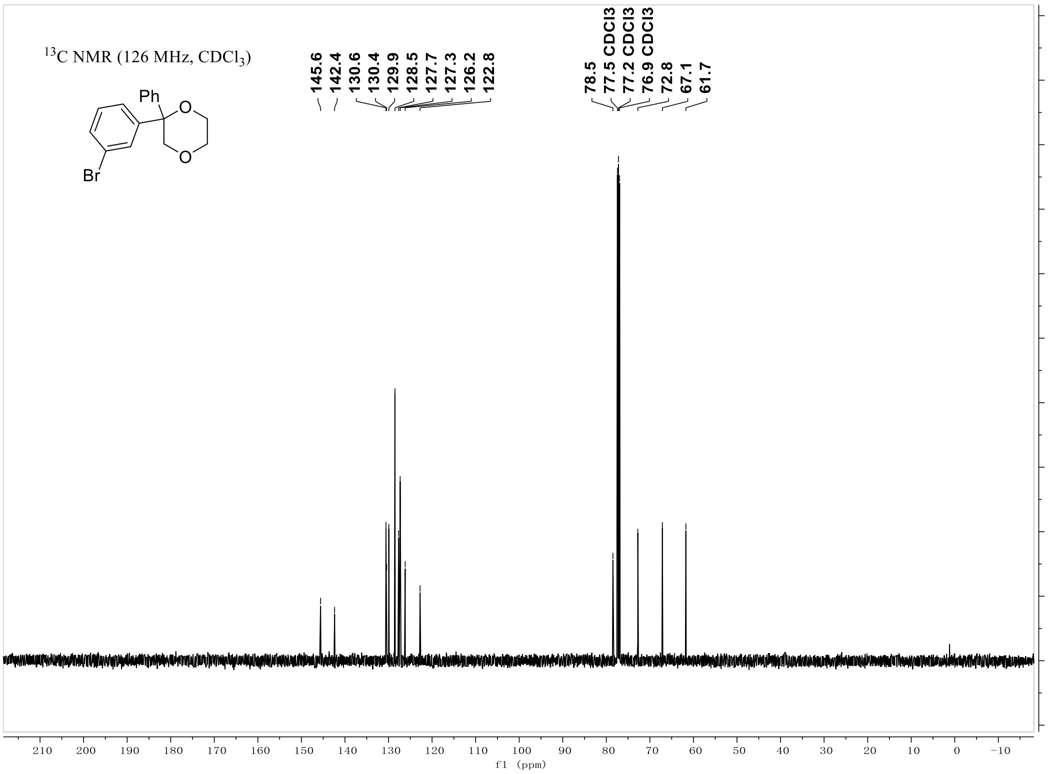

Supplementary Fig. 12. ^{1}H NMR and ^{13}C NMR spectra for compound 14

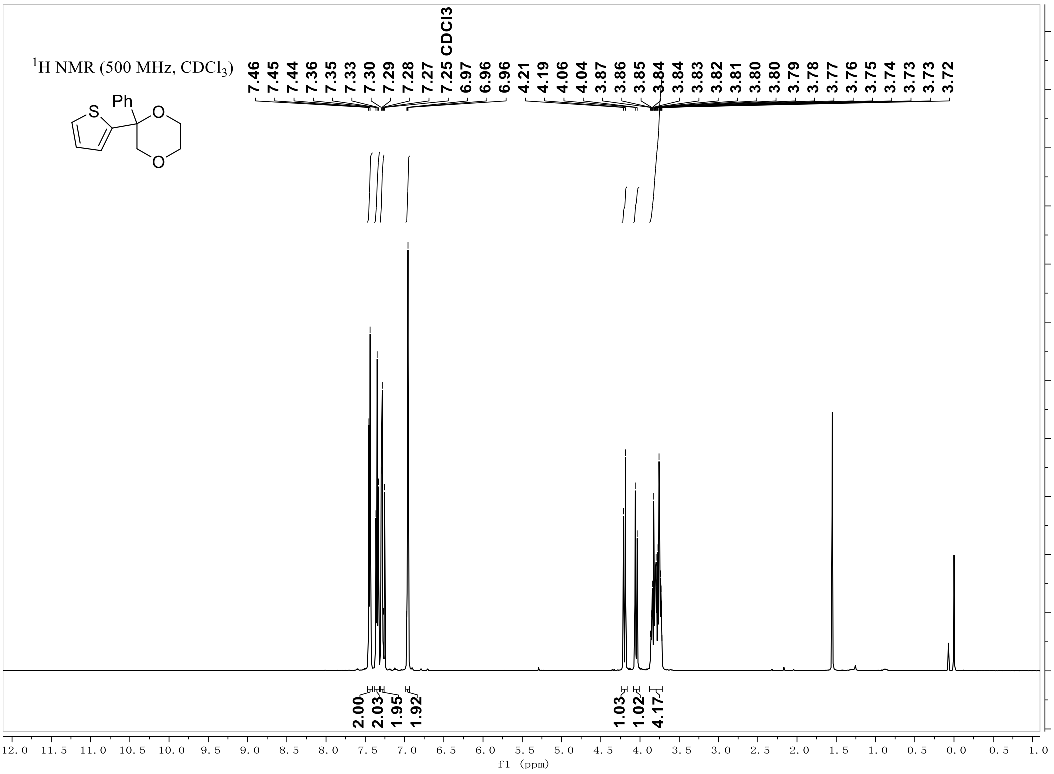

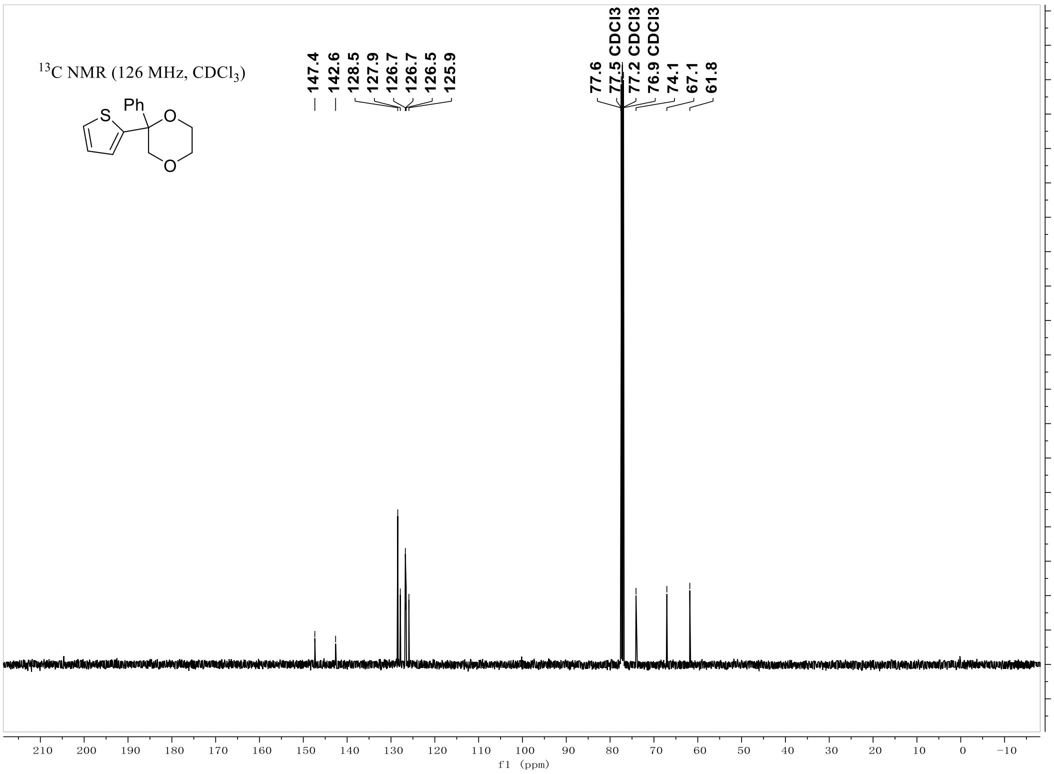

Supplementary Fig. 13. ^{1}H NMR and ^{13}C NMR spectra for compound 15

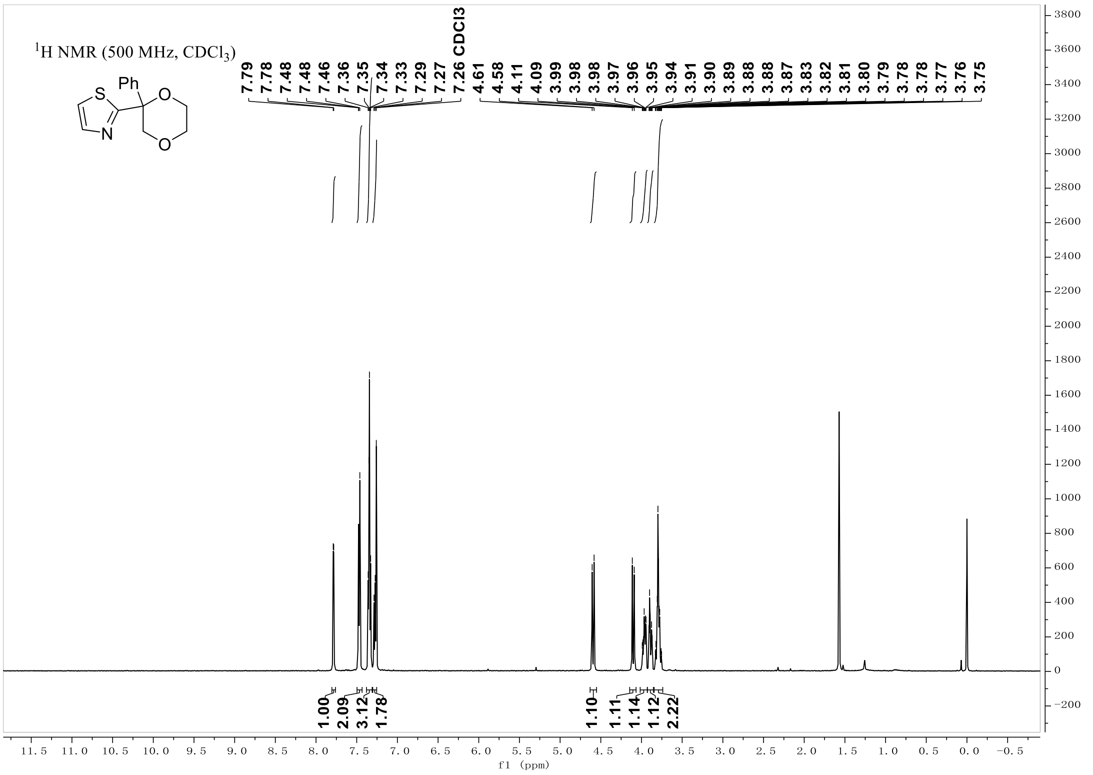

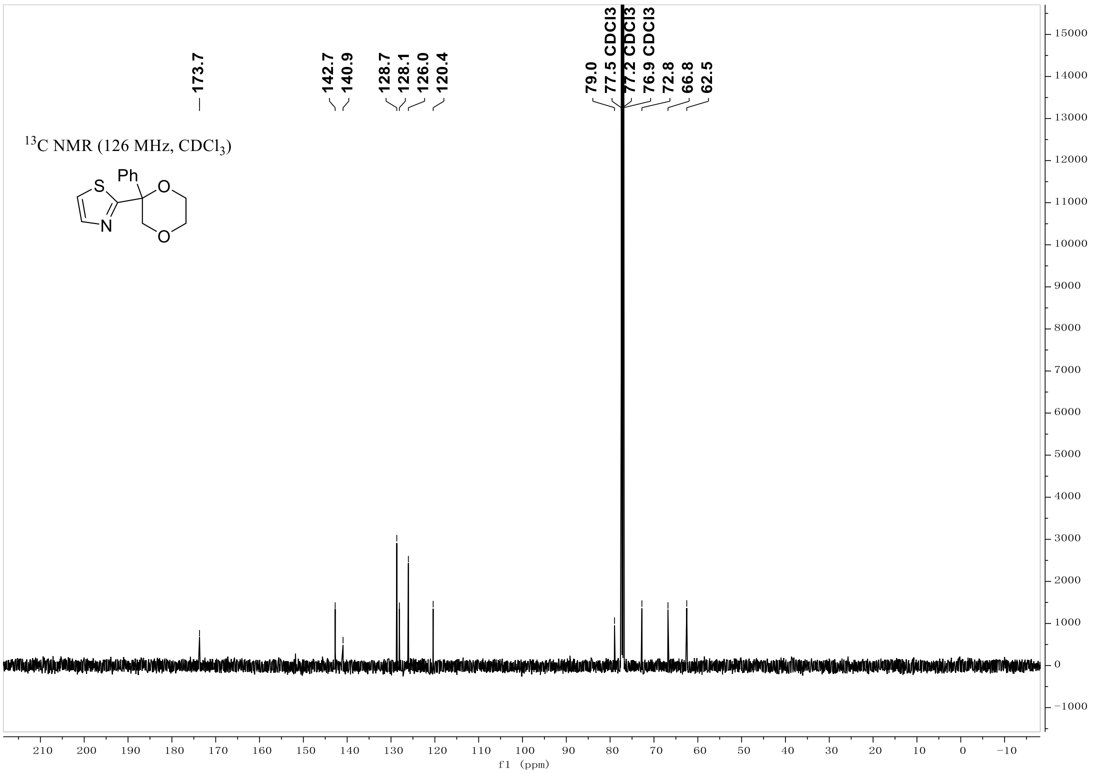

Supplementary Fig. 14. ^{1}H NMR and ^{13}C NMR spectra for compound 16

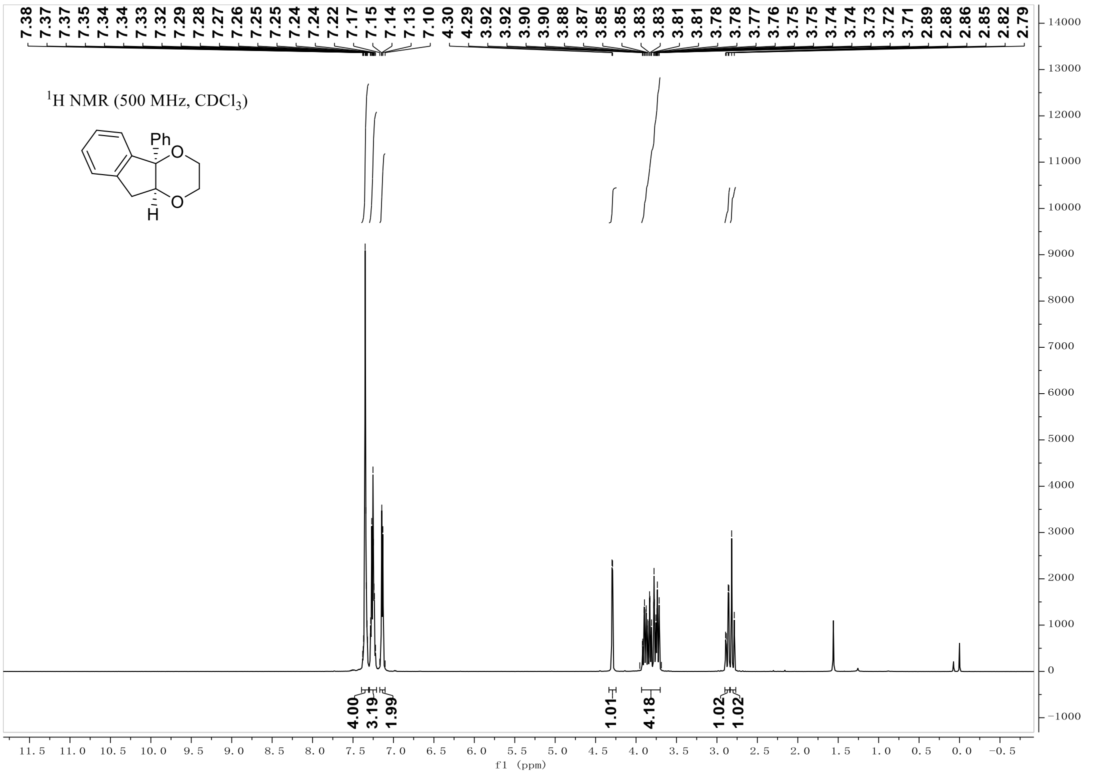

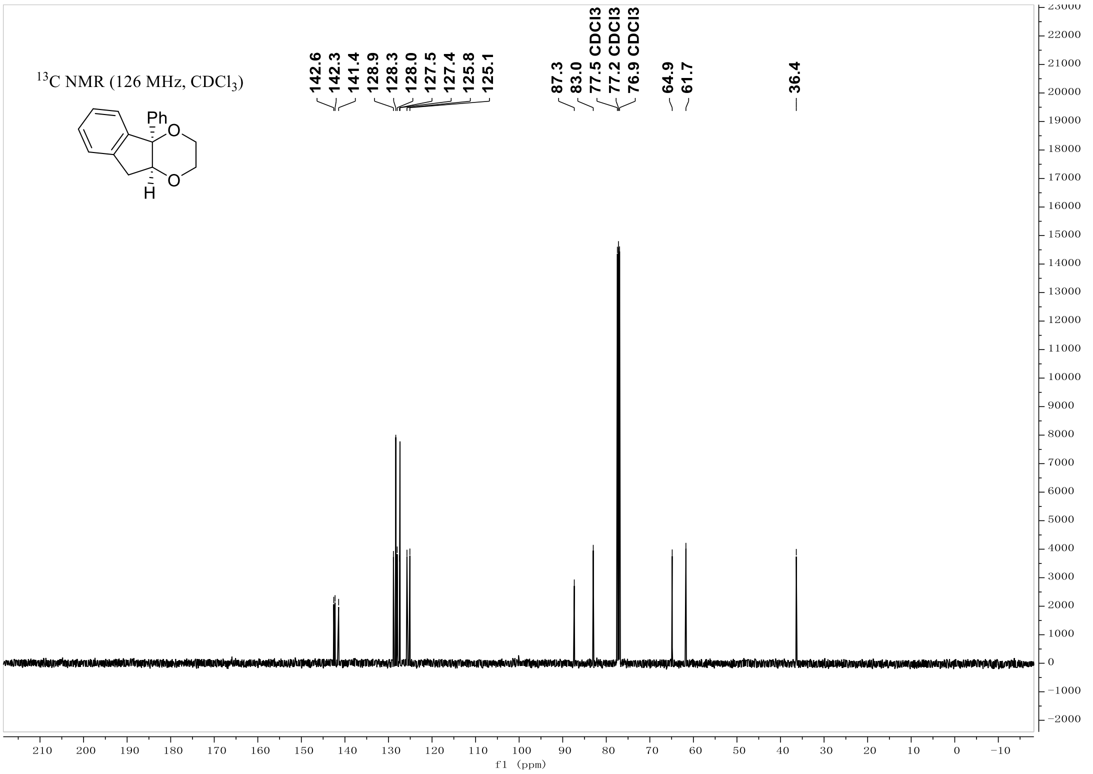

Supplementary Fig. 15. ^{1}H NMR and ^{13}C NMR spectra for compound 17

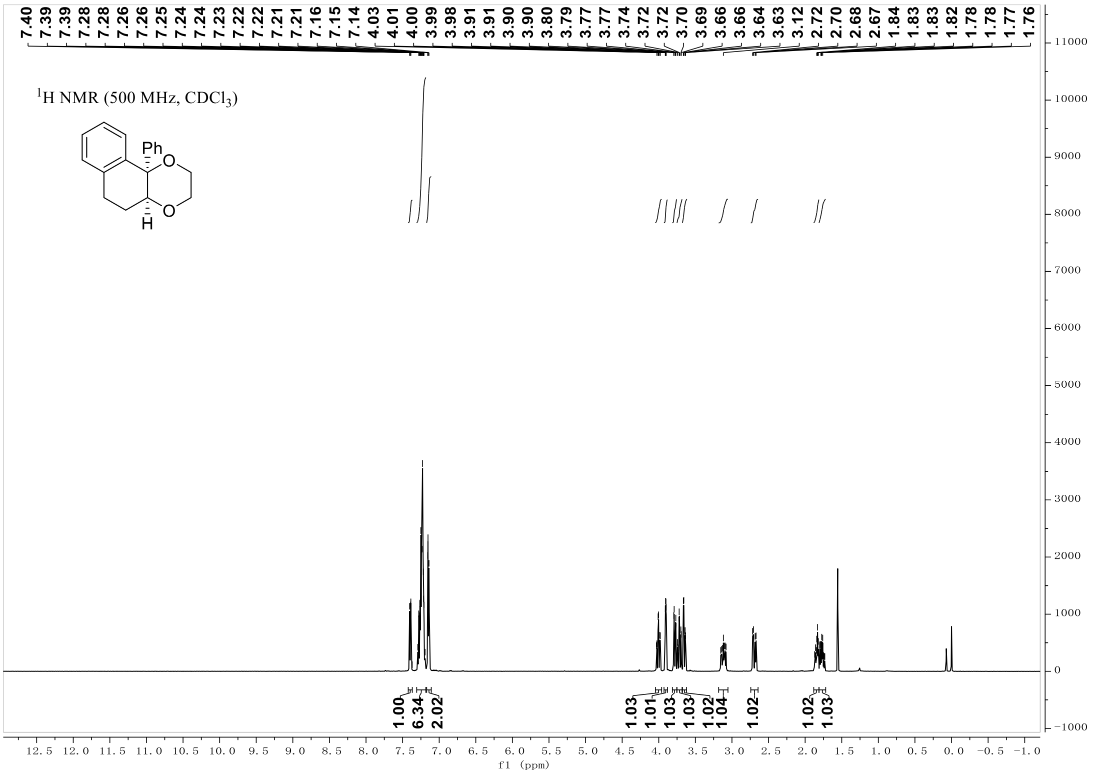

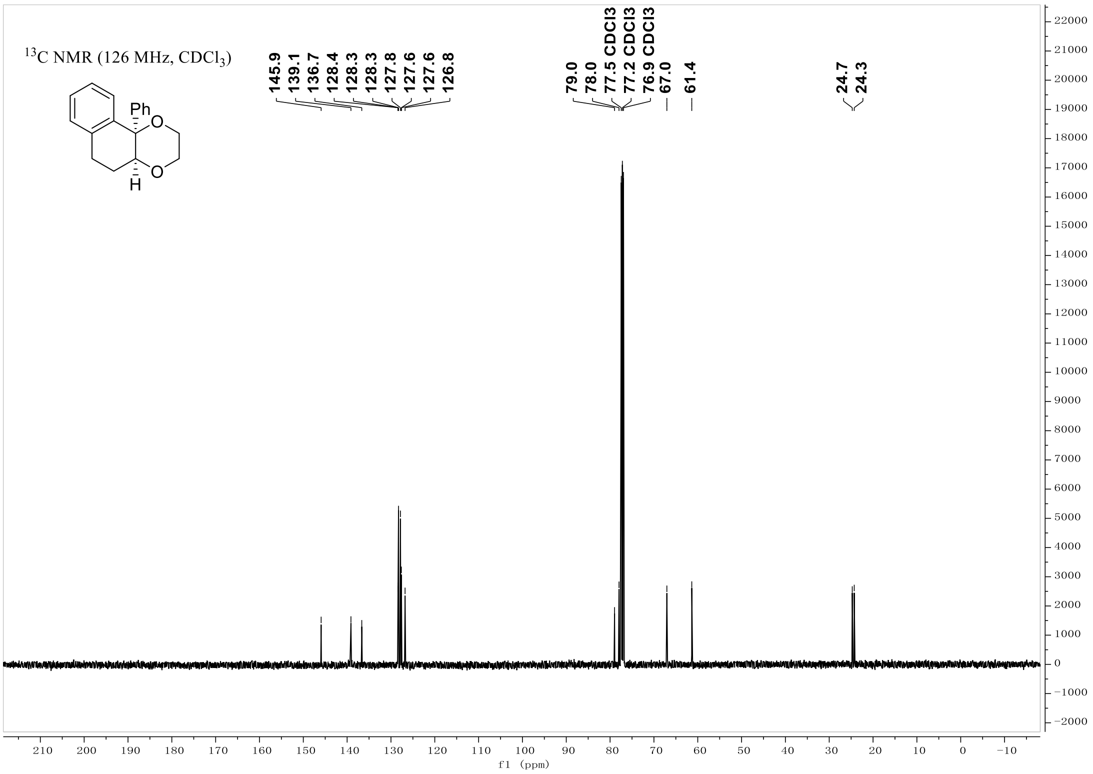

Supplementary Fig. 16. ^{1}H NMR and ^{13}C NMR spectra for compound 18

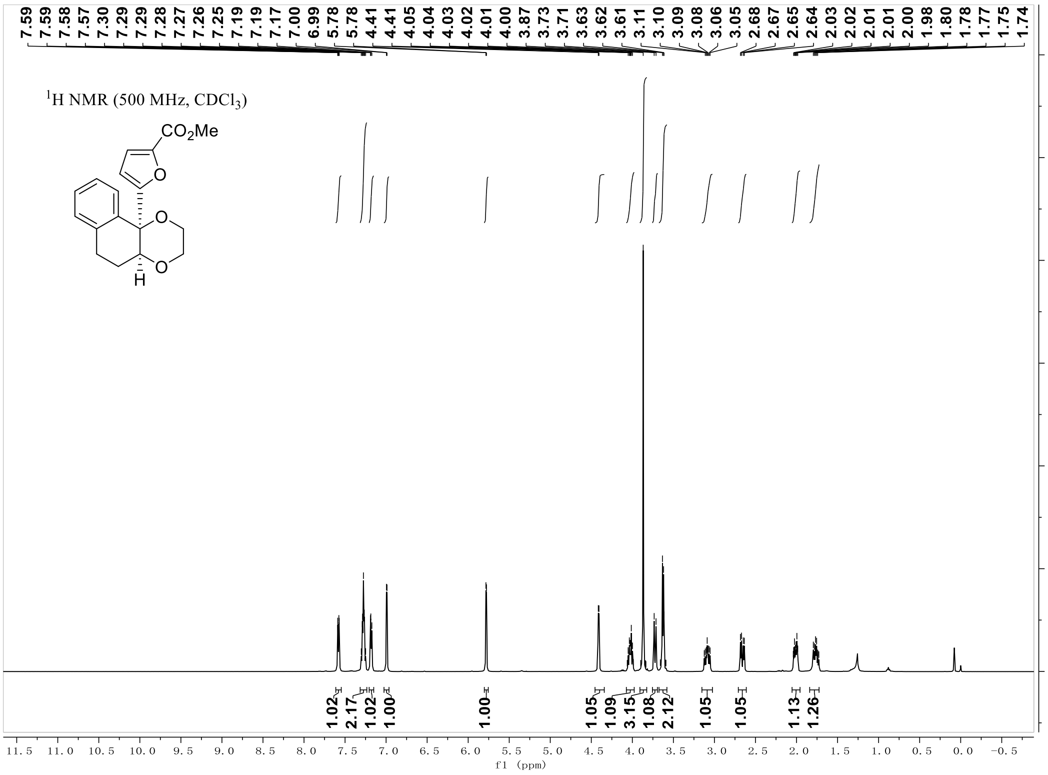

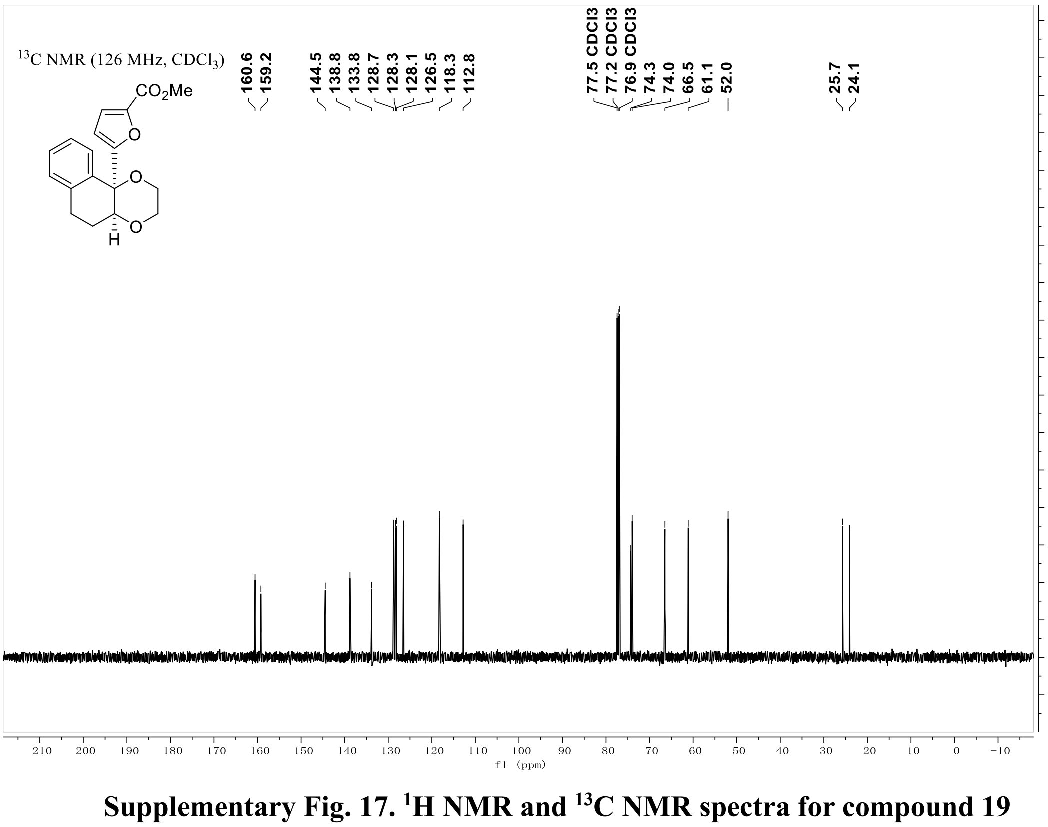

Supplementary Fig. 17. ^{1}H NMR and ^{13}C NMR spectra for compound 19

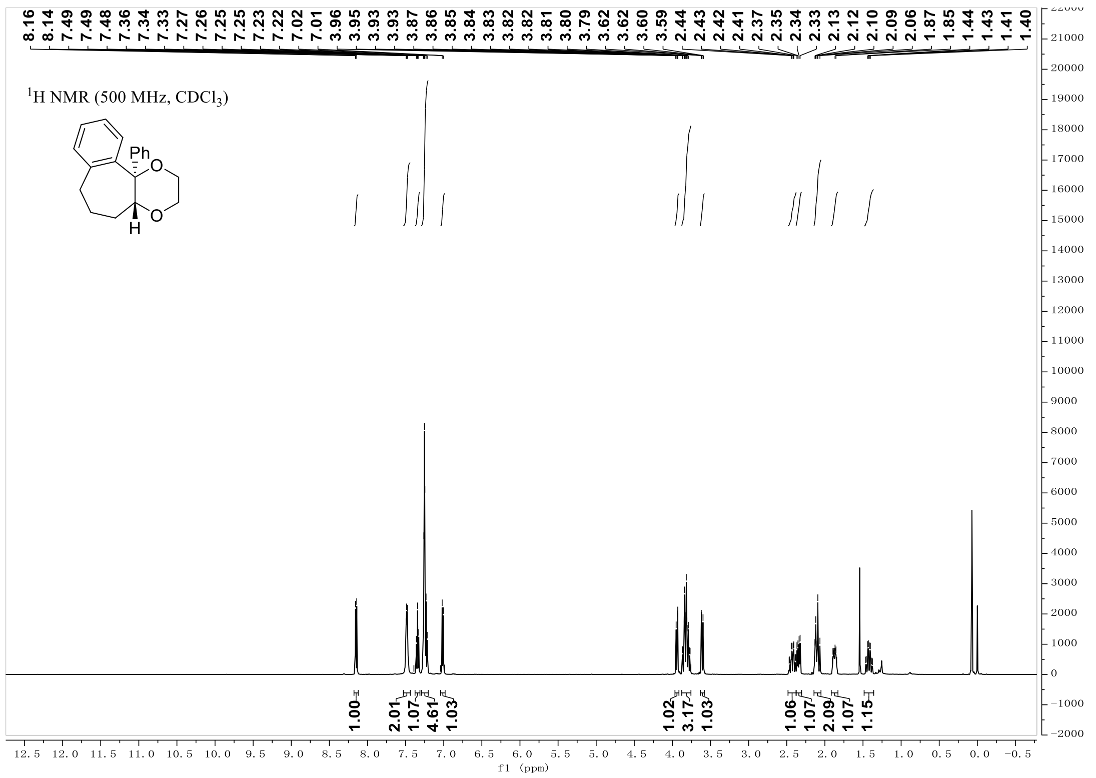

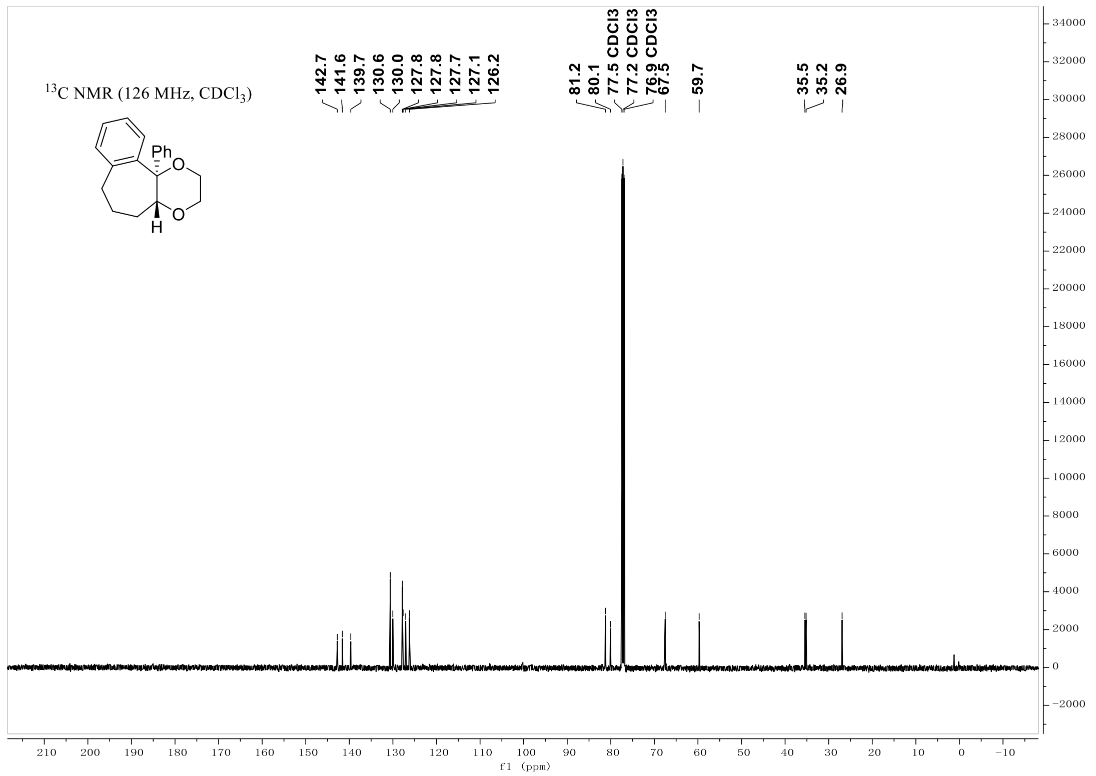

Supplementary Fig. 18. ^{1}H NMR and ^{13}C NMR spectra for compound trans-20

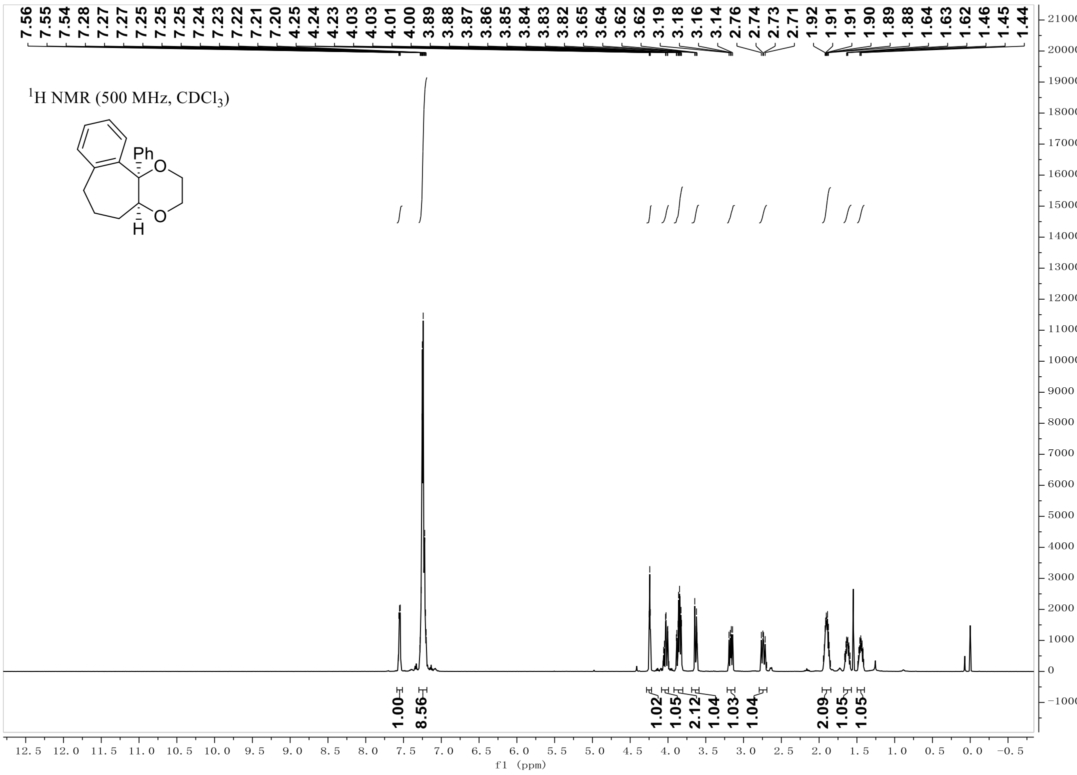

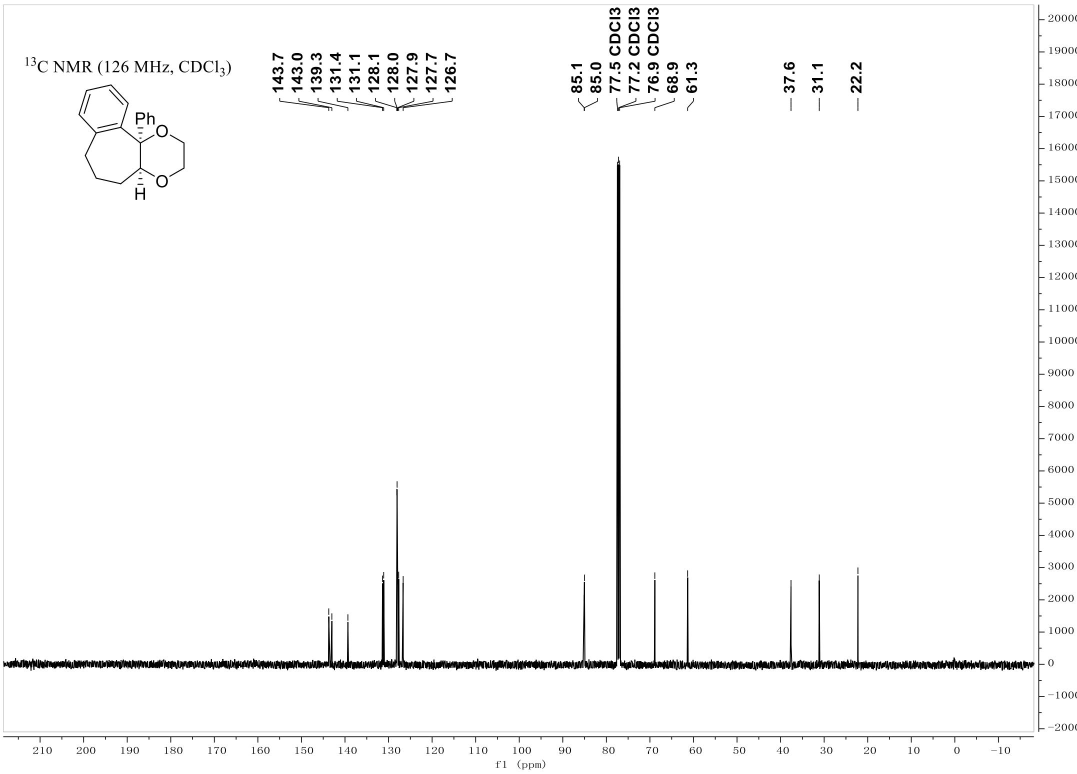

Supplementary Fig. 19. ^1H NMR and ^{13}C NMR spectra for compound cis-20

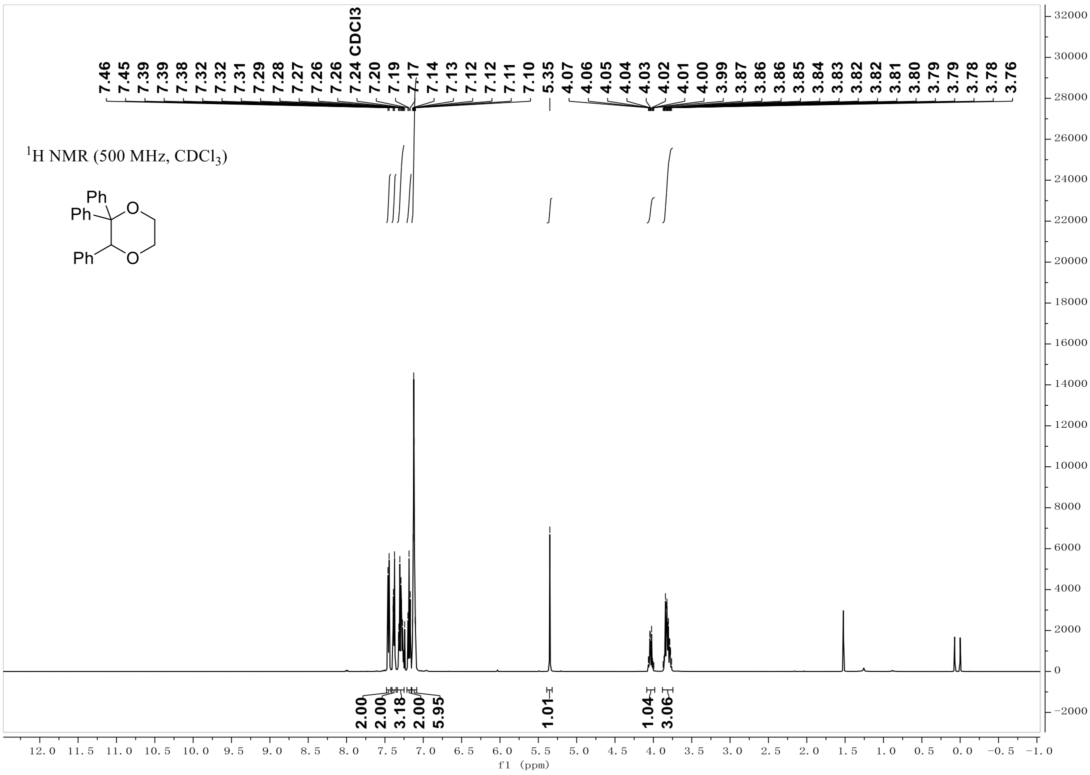

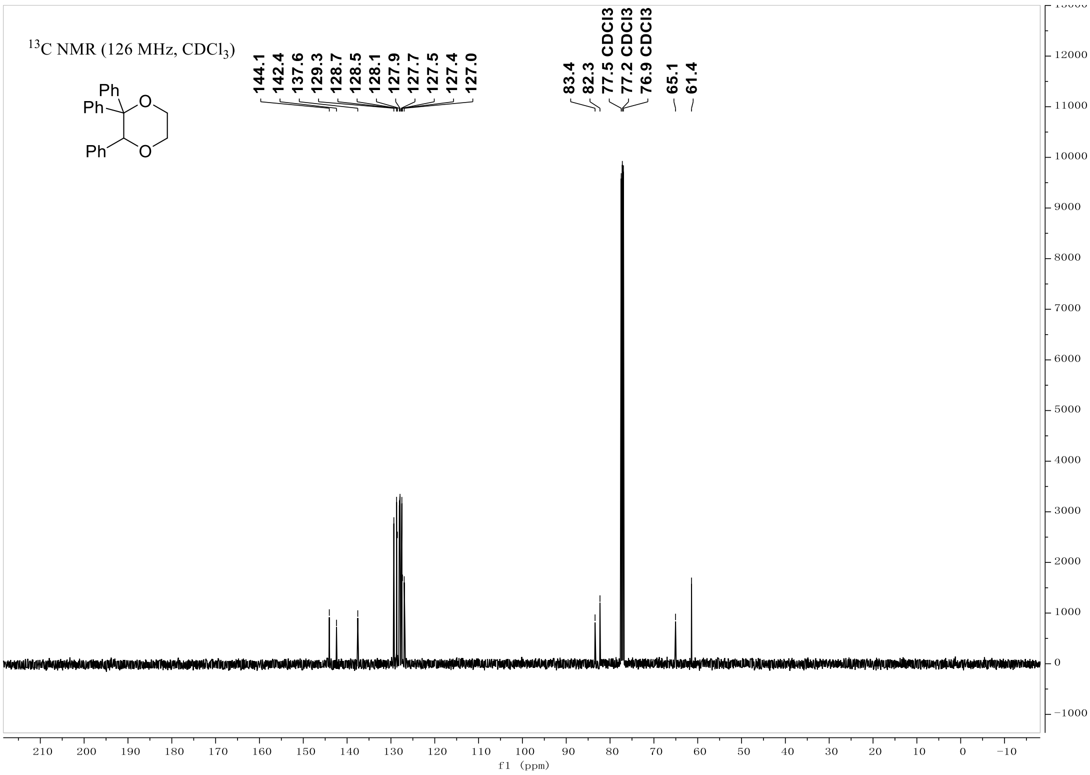

Supplementary Fig. 20. ^{1}H NMR and ^{13}C NMR spectra for compound 21

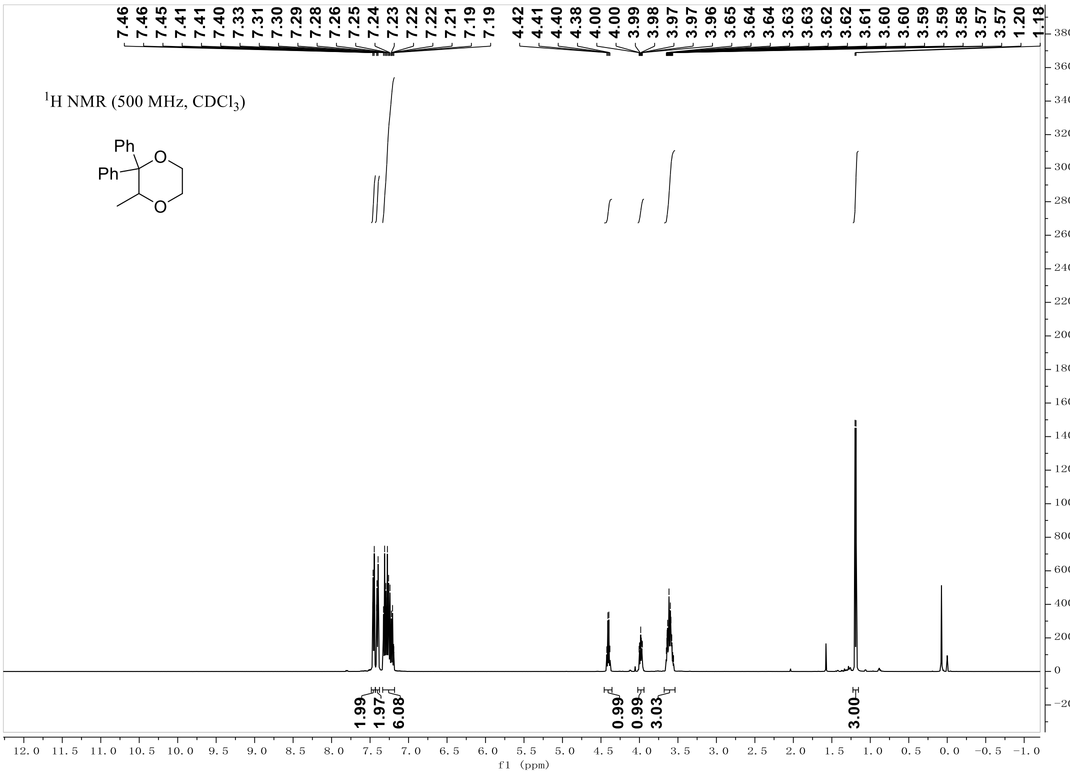

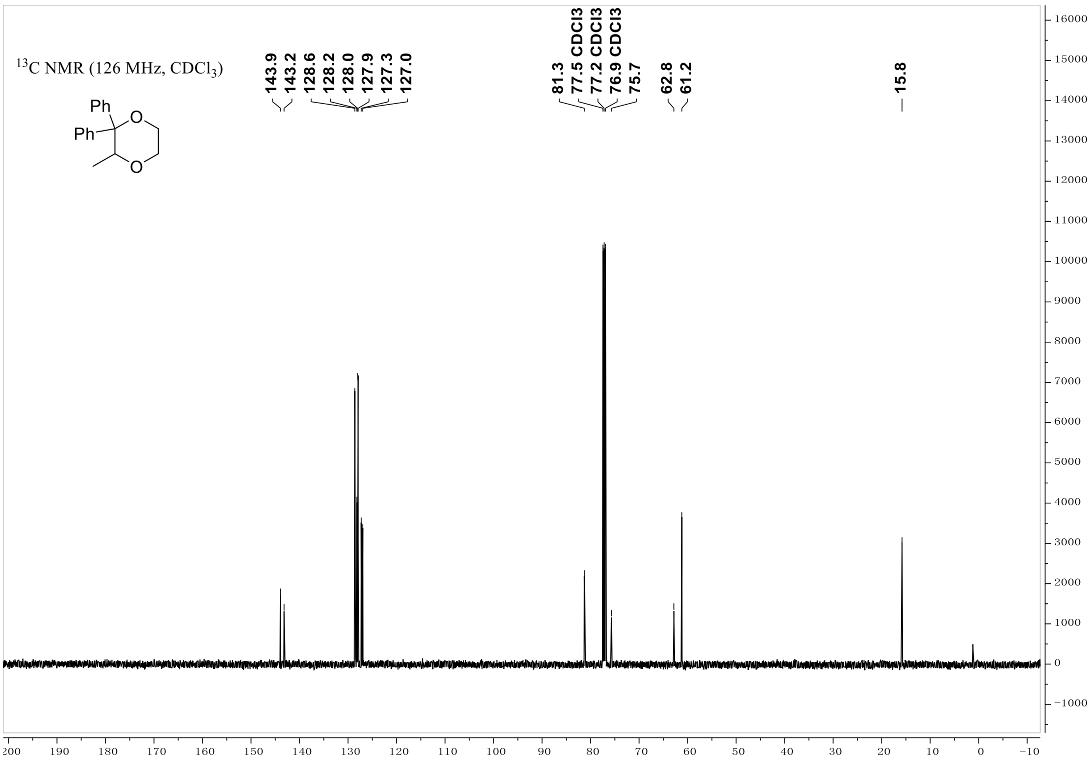

Supplementary Fig. 21. ^{1}H NMR and ^{13}C NMR spectra for compound 22

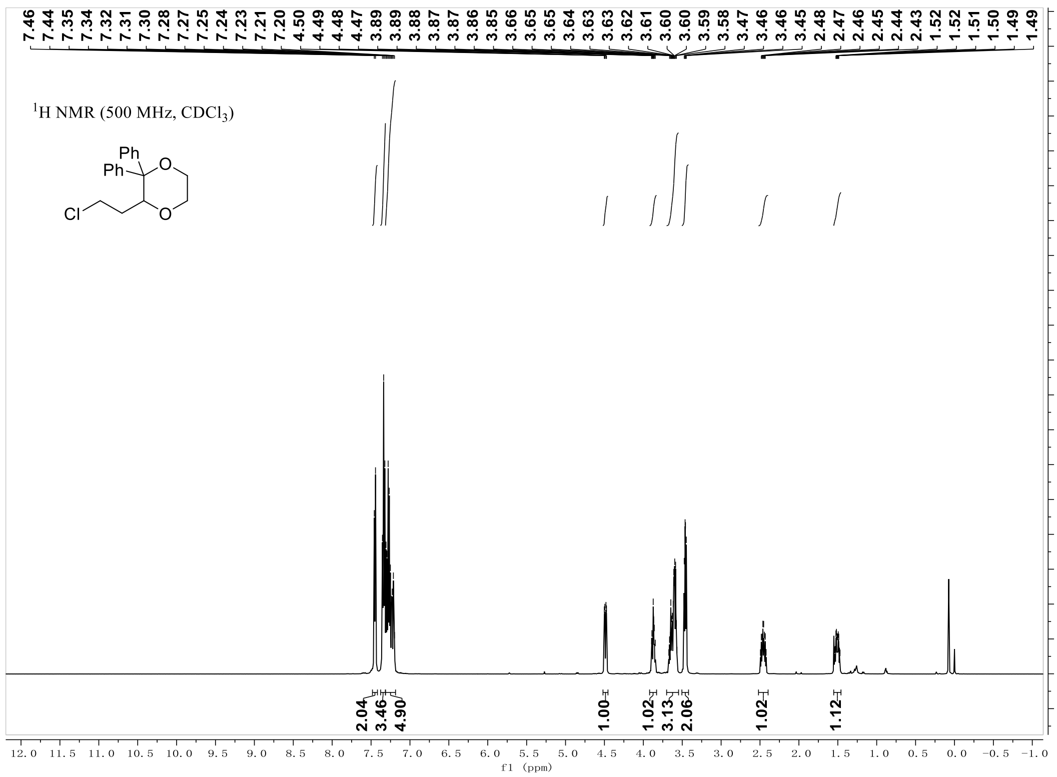

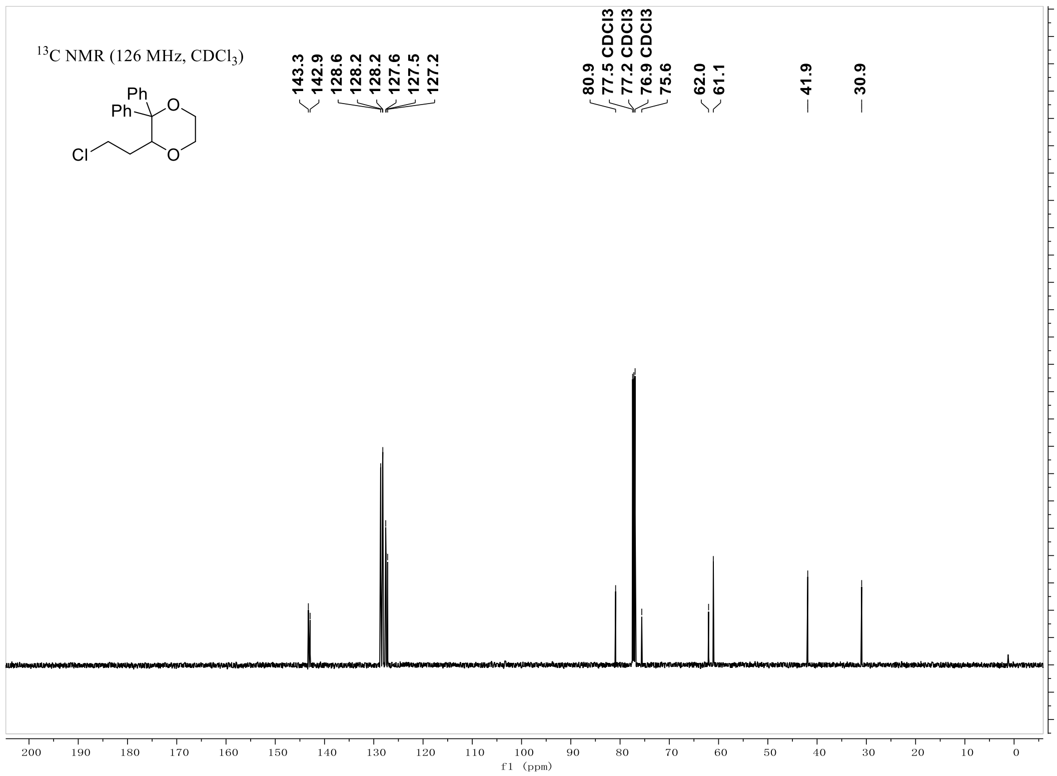

Supplementary Fig. 22. ^{1}H NMR and ^{13}C NMR spectra for compound 23

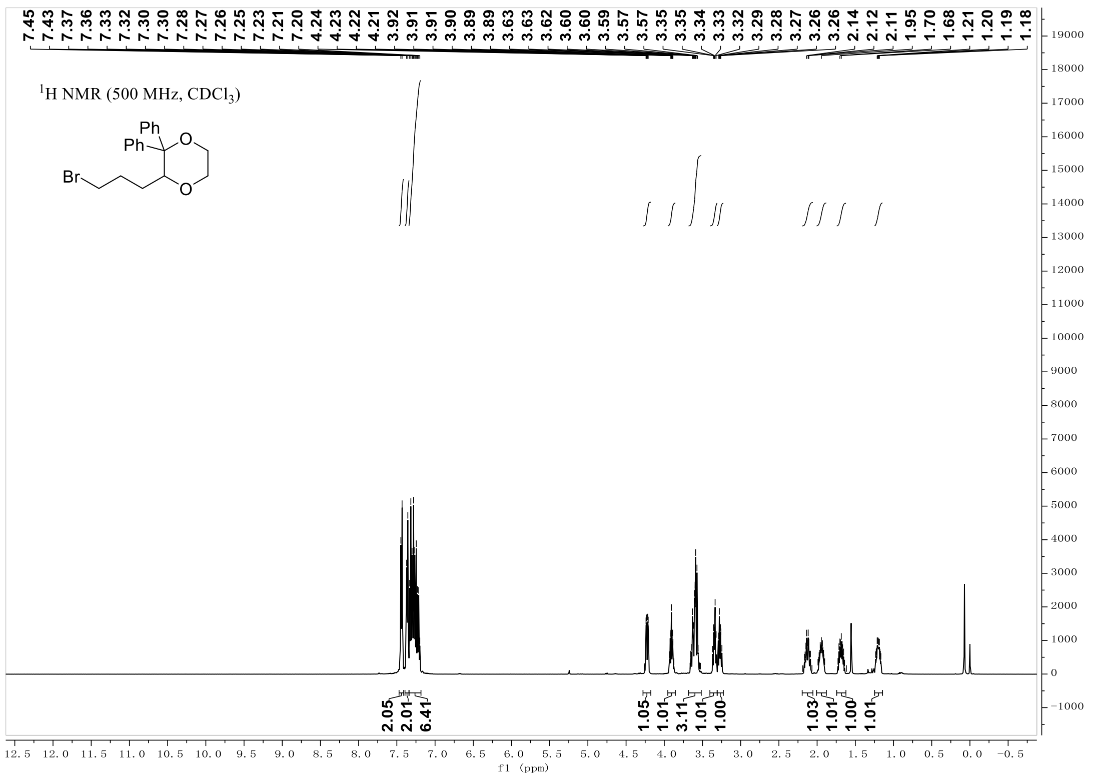

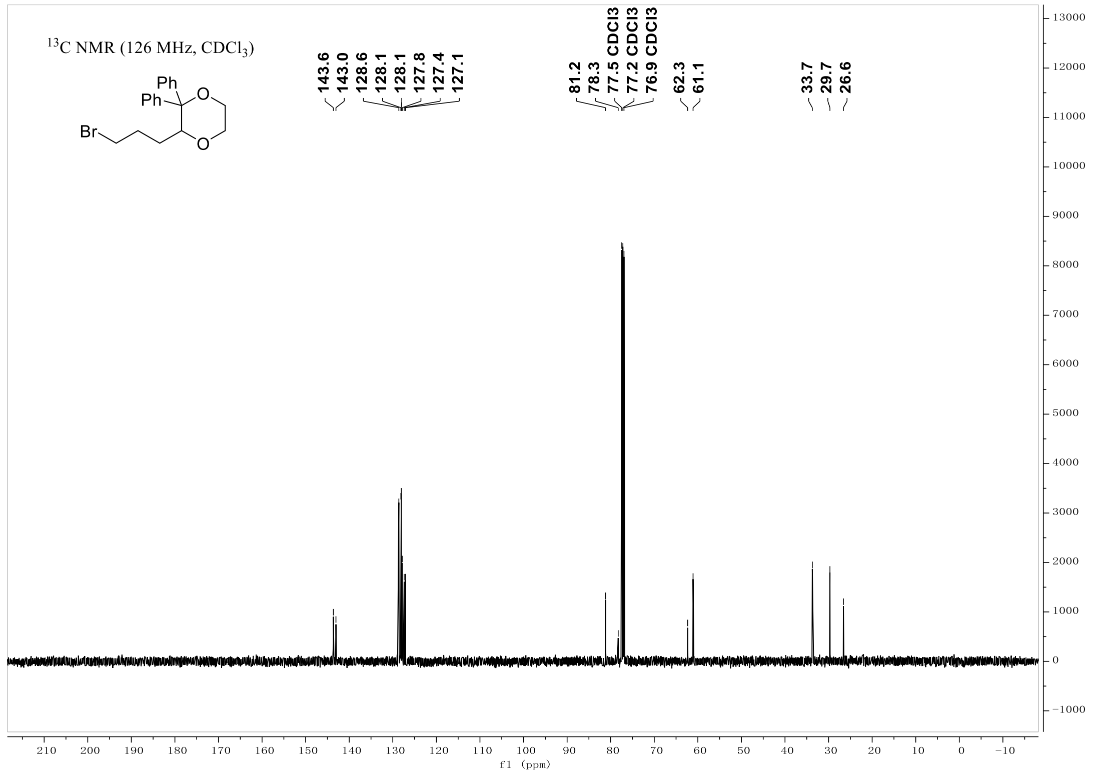

Supplementary Fig. 23. ^{1}H NMR and ^{13}C NMR spectra for compound 24

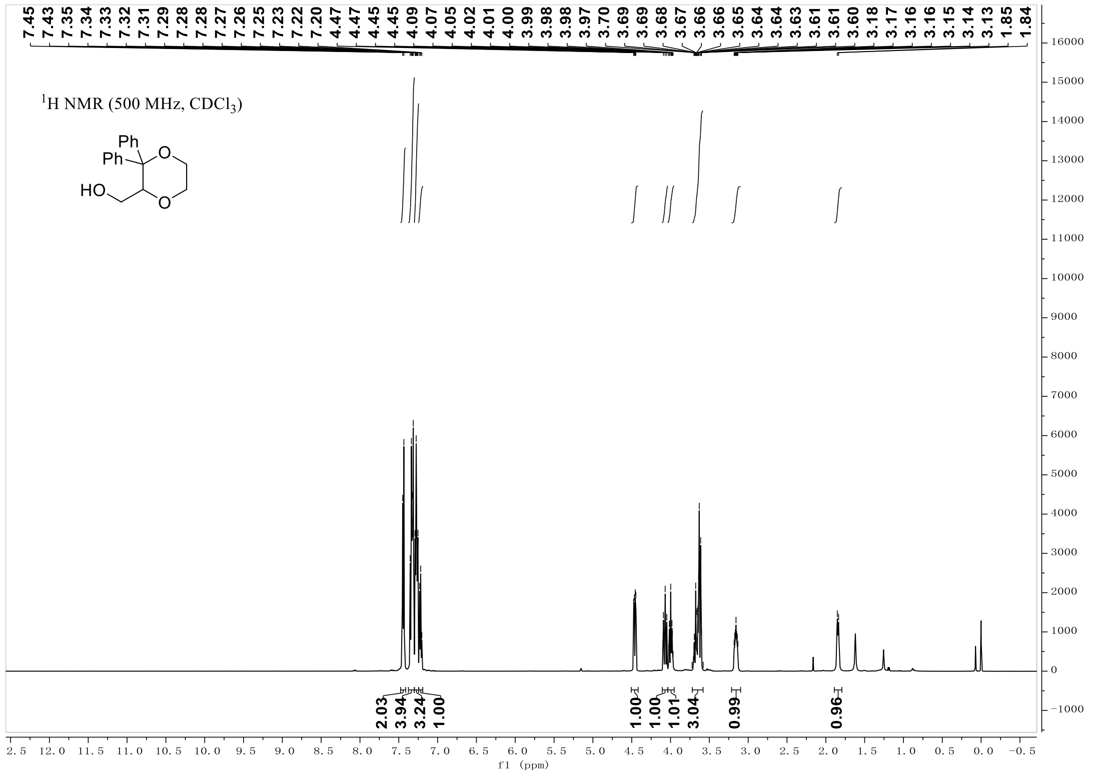

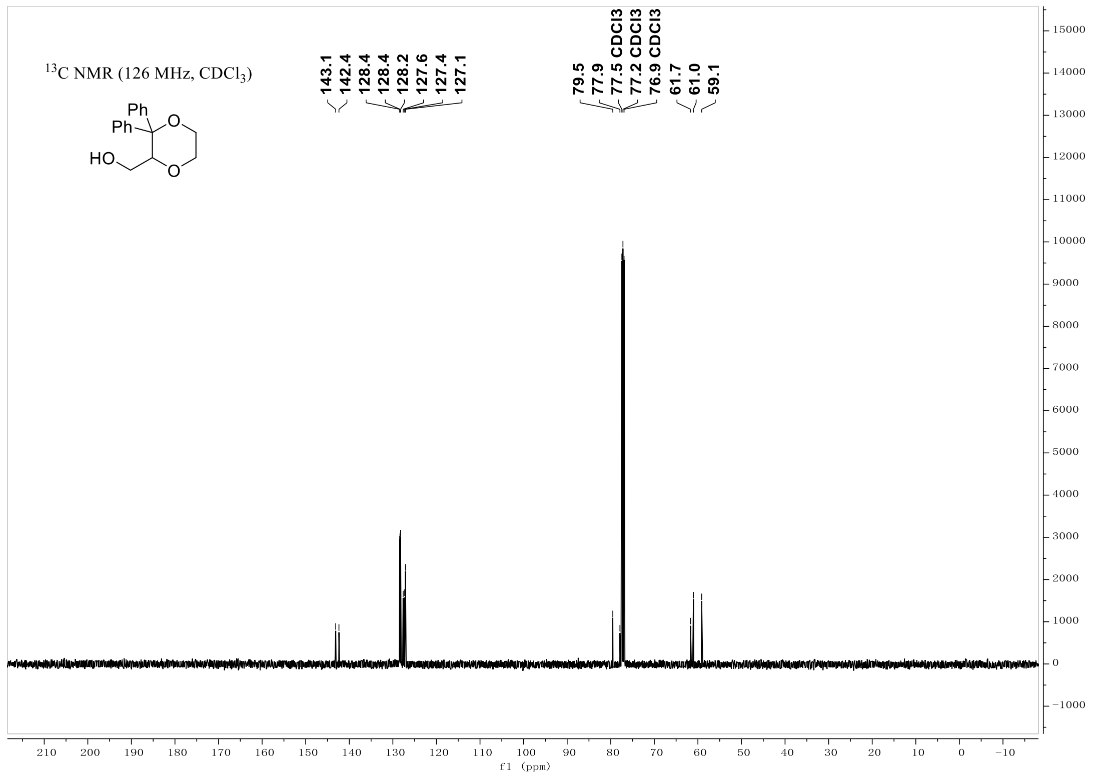

Supplementary Fig. 24. ^{1}H NMR and ^{13}C NMR spectra for compound 25

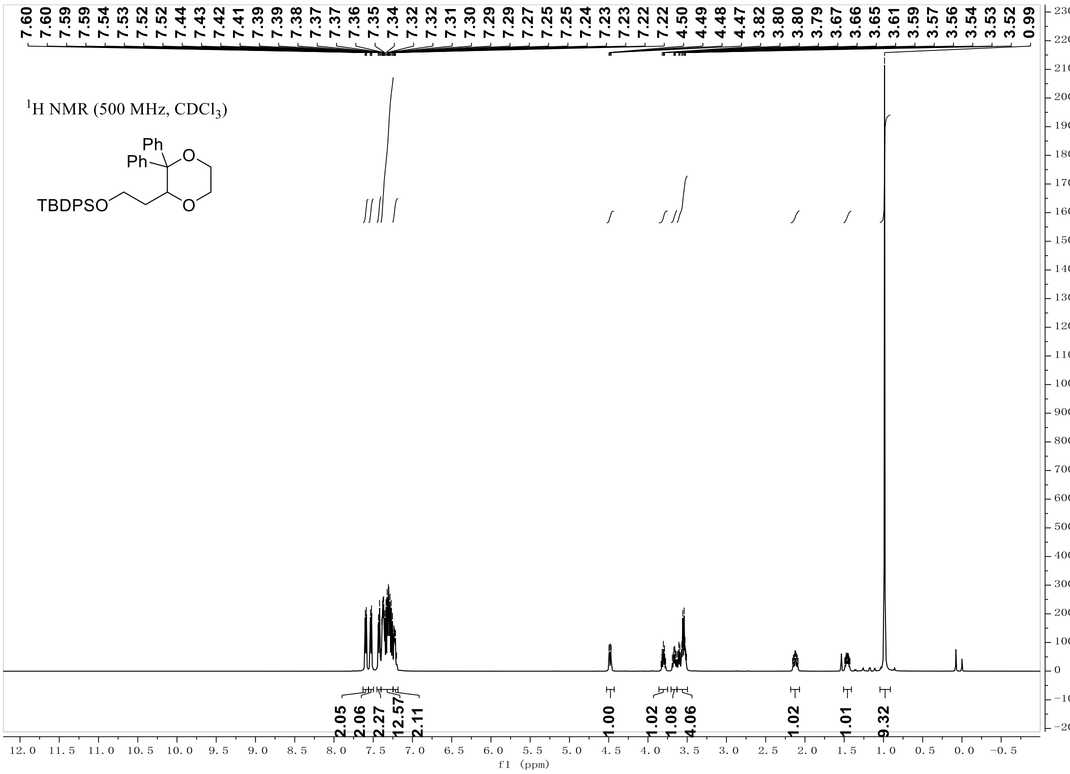

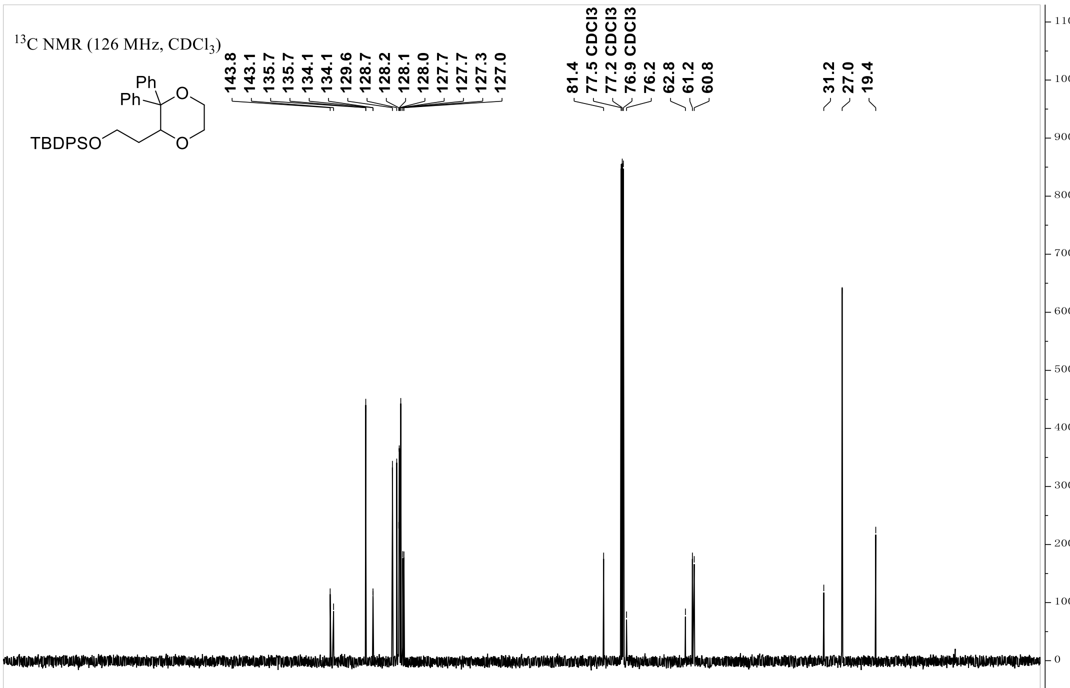

Supplementary Fig. 25. ^{1}H NMR and ^{13}C NMR spectra for compound 26

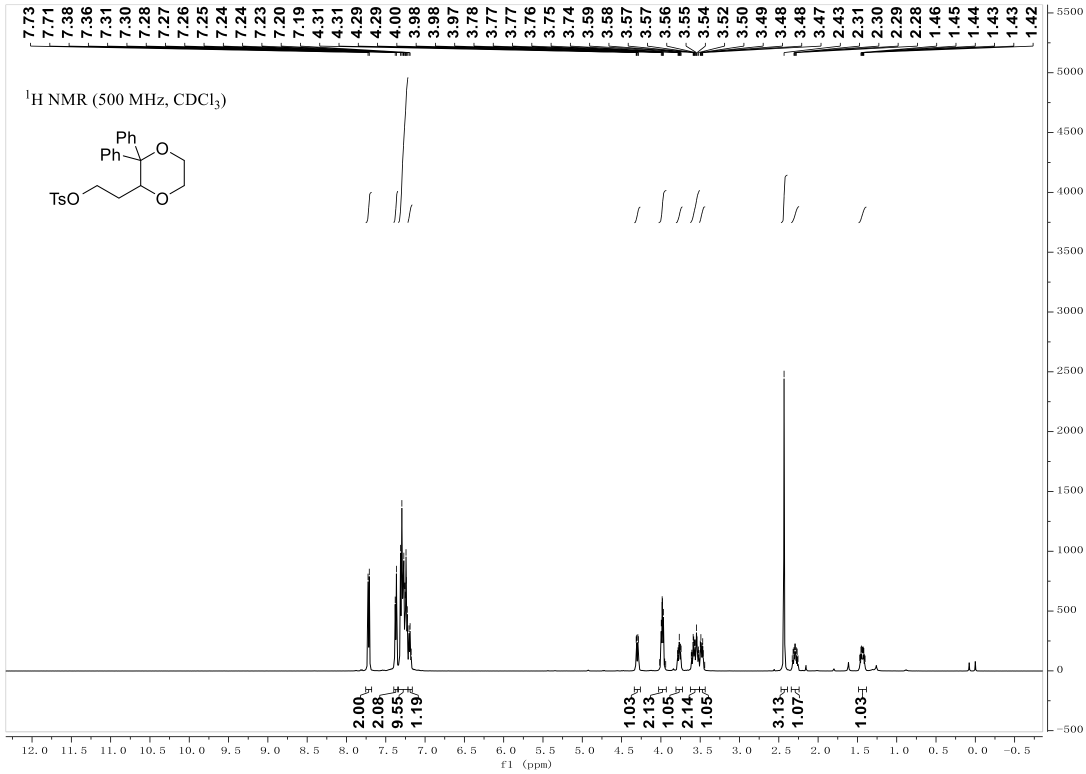

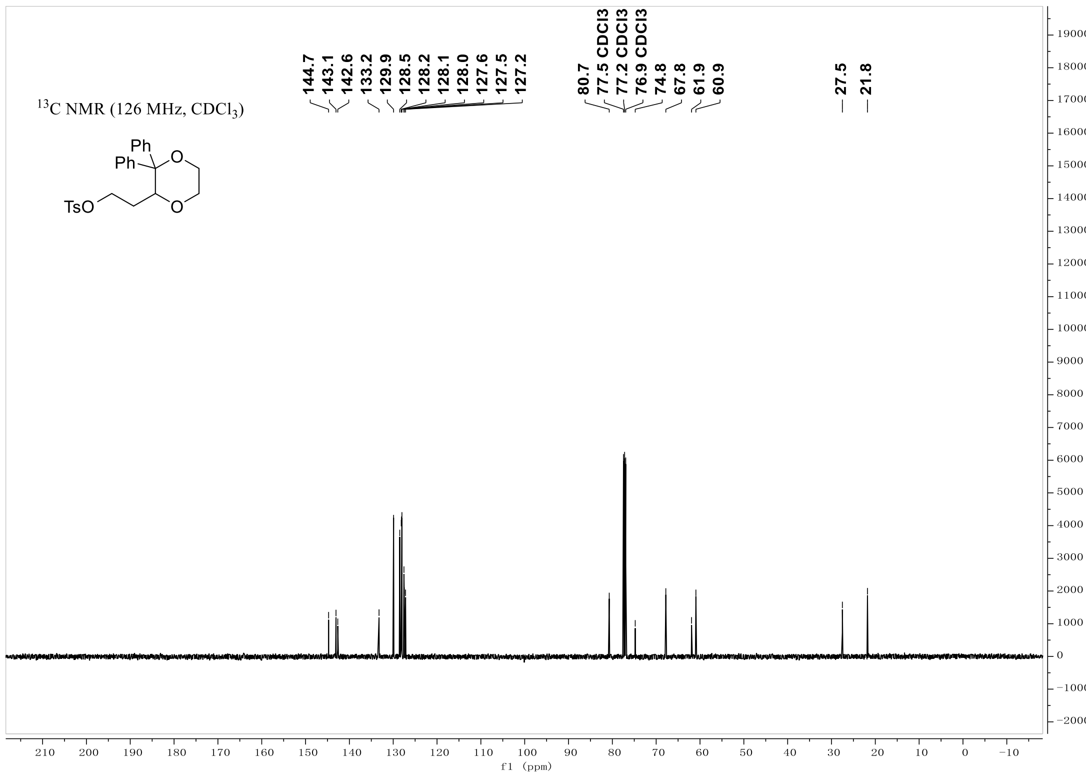

Supplementary Fig. 26. ^{1}H NMR and ^{13}C NMR spectra for compound 27

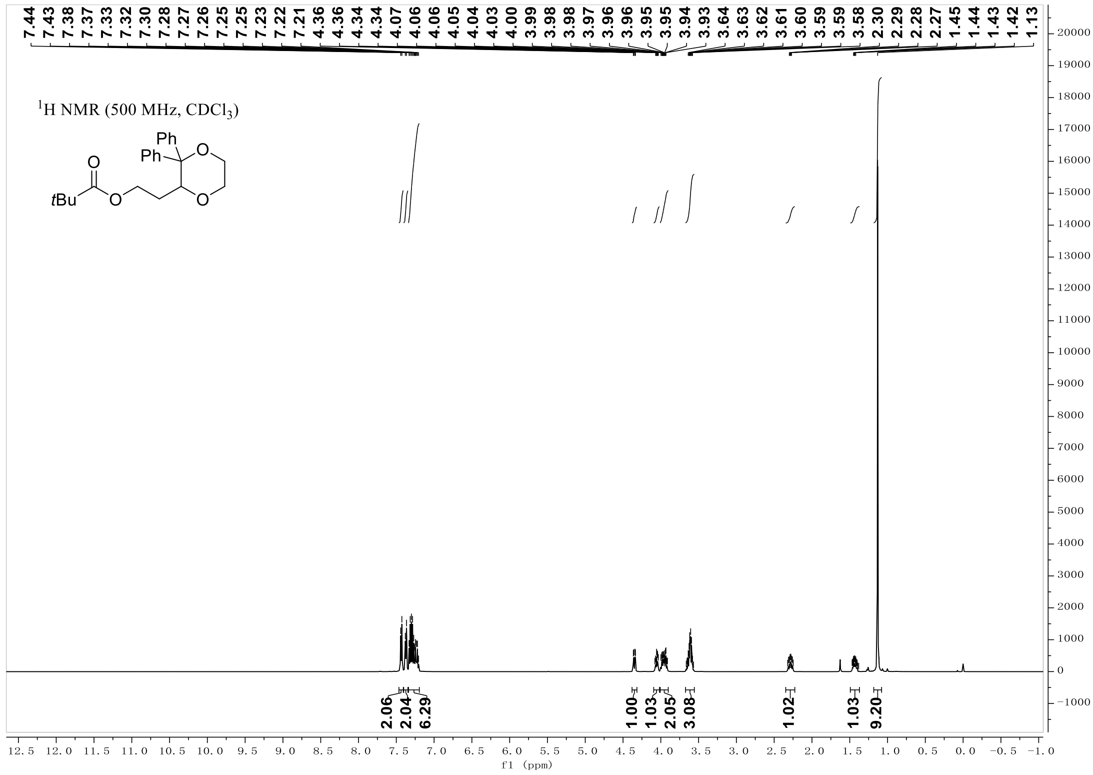

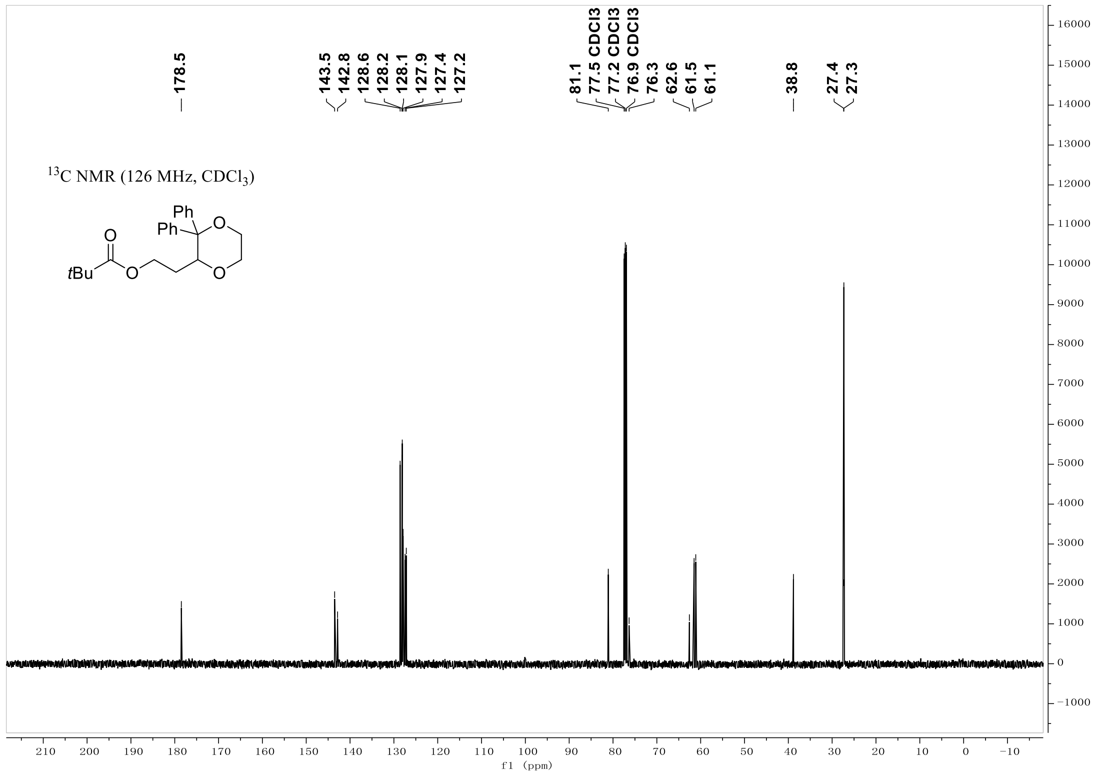

Supplementary Fig. 27. ^{1}H NMR and ^{13}C NMR spectra for compound 28

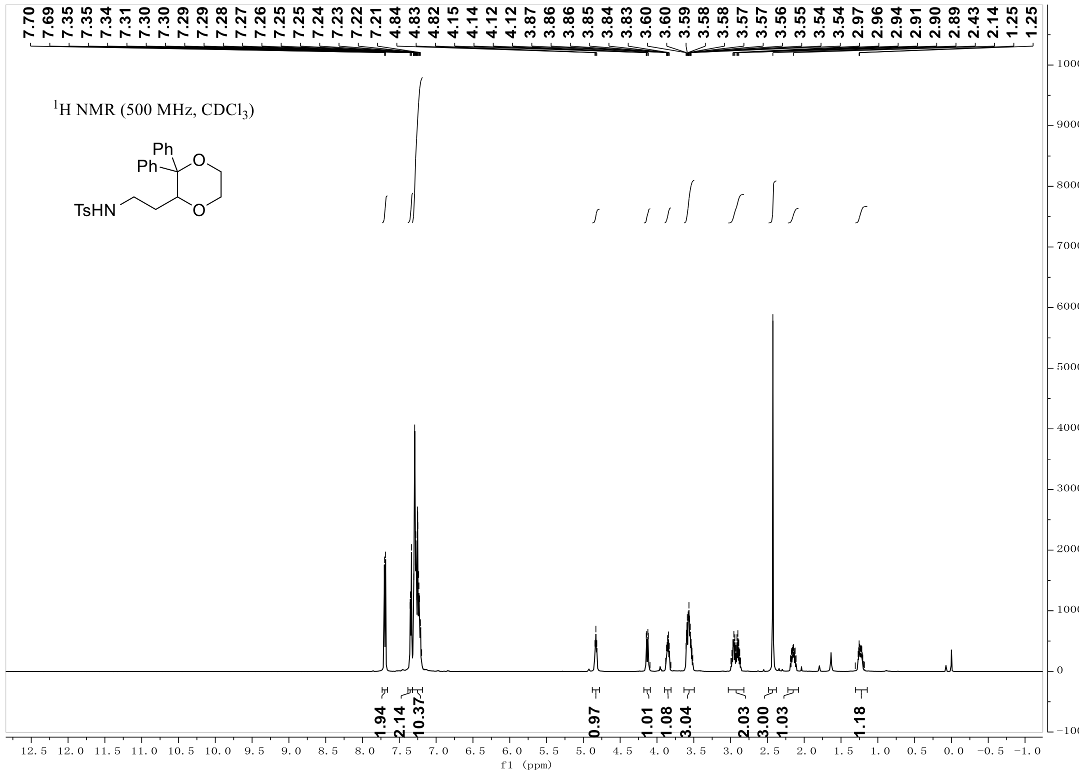

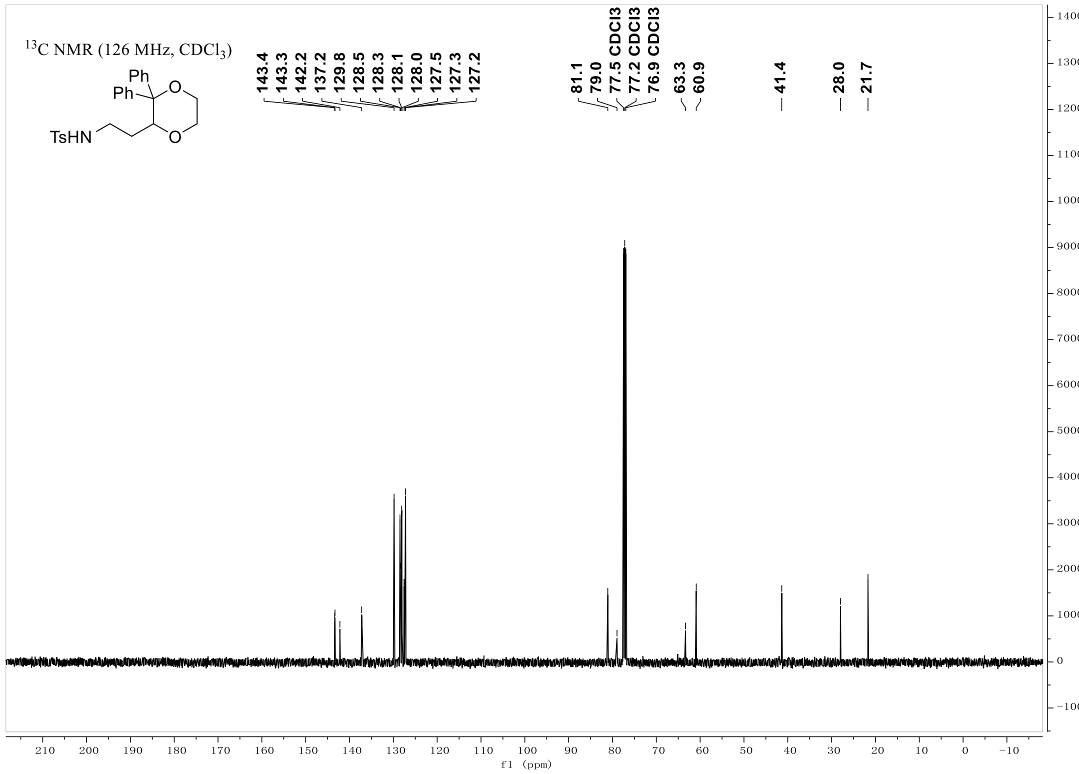

Supplementary Fig. 28. ^{1}H NMR and ^{13}C NMR spectra for compound 29

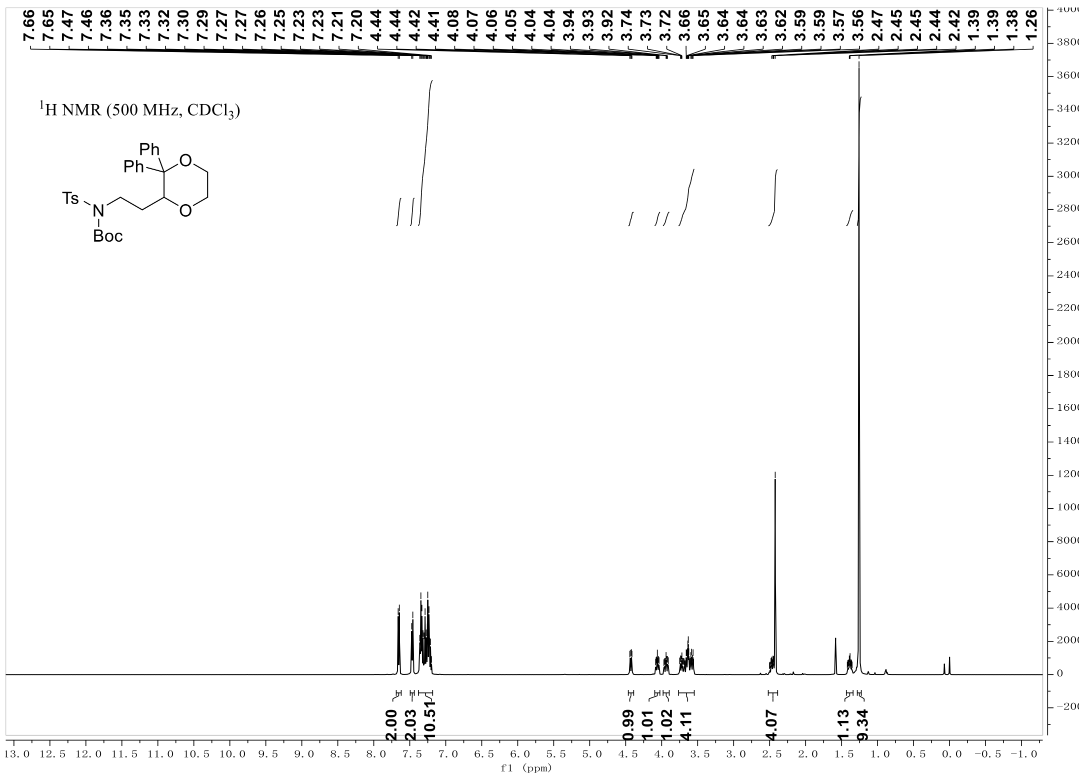

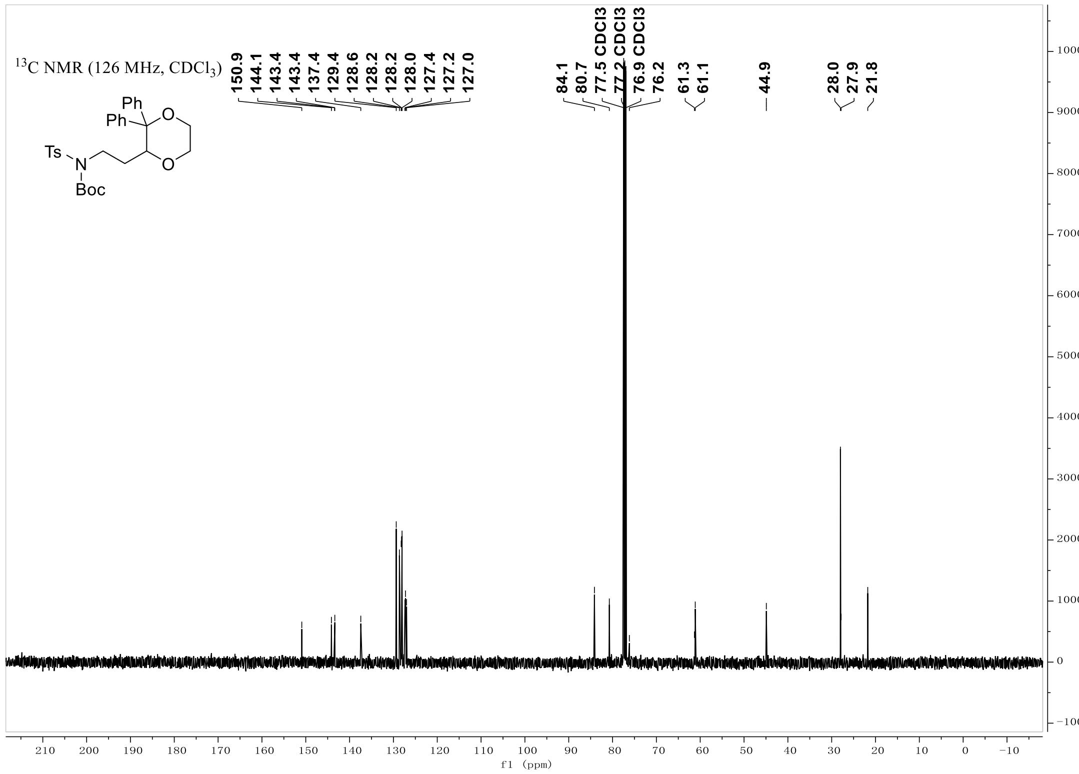

Supplementary Fig. 29. ^{1}H NMR and ^{13}C NMR spectra for compound 30

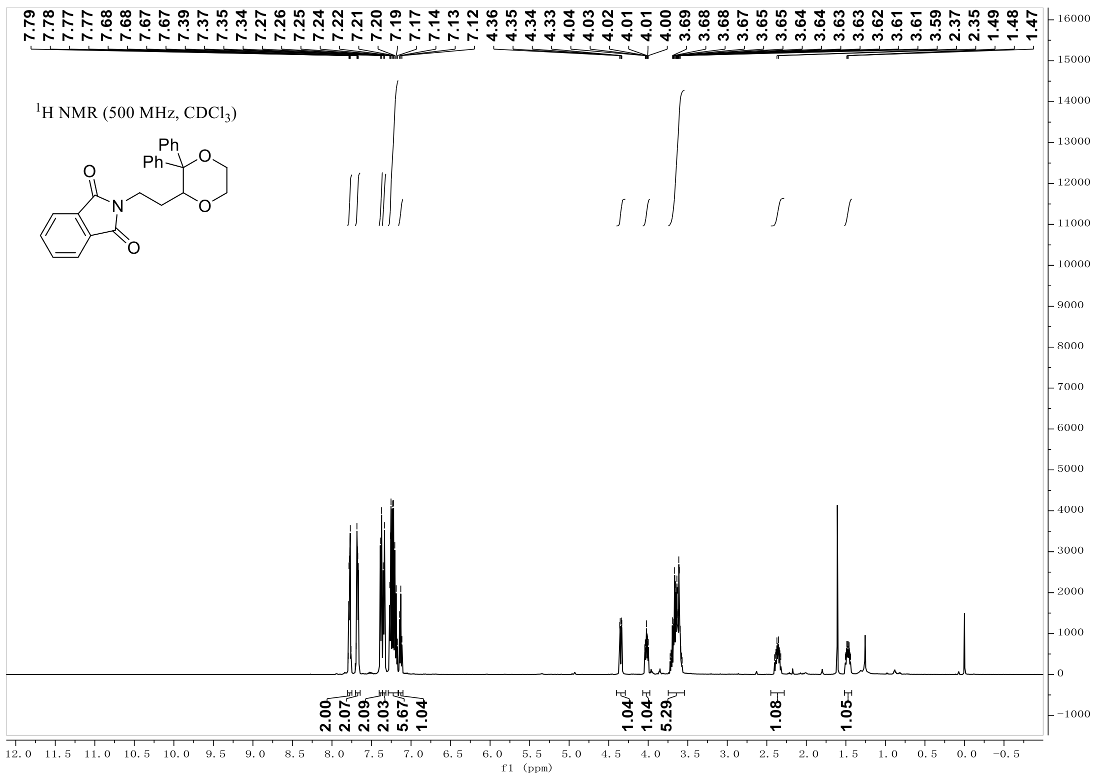

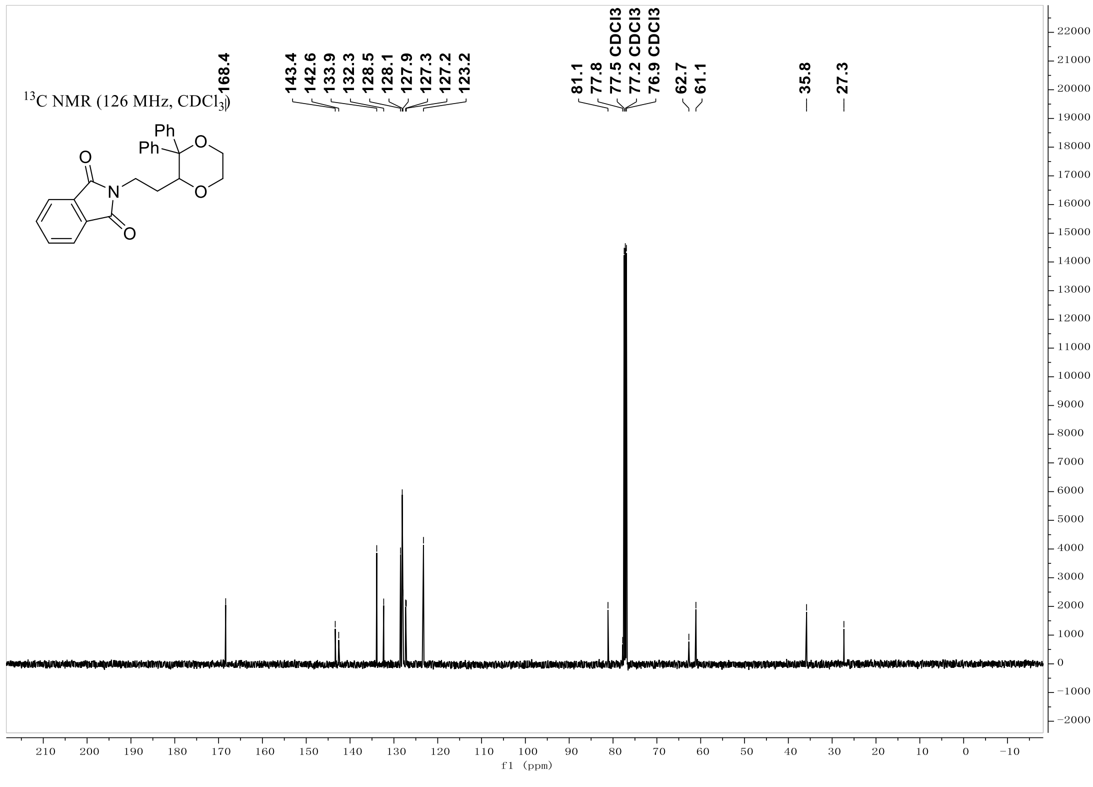

Supplementary Fig. 30. ^{1}H NMR and ^{13}C NMR spectra for compound 31

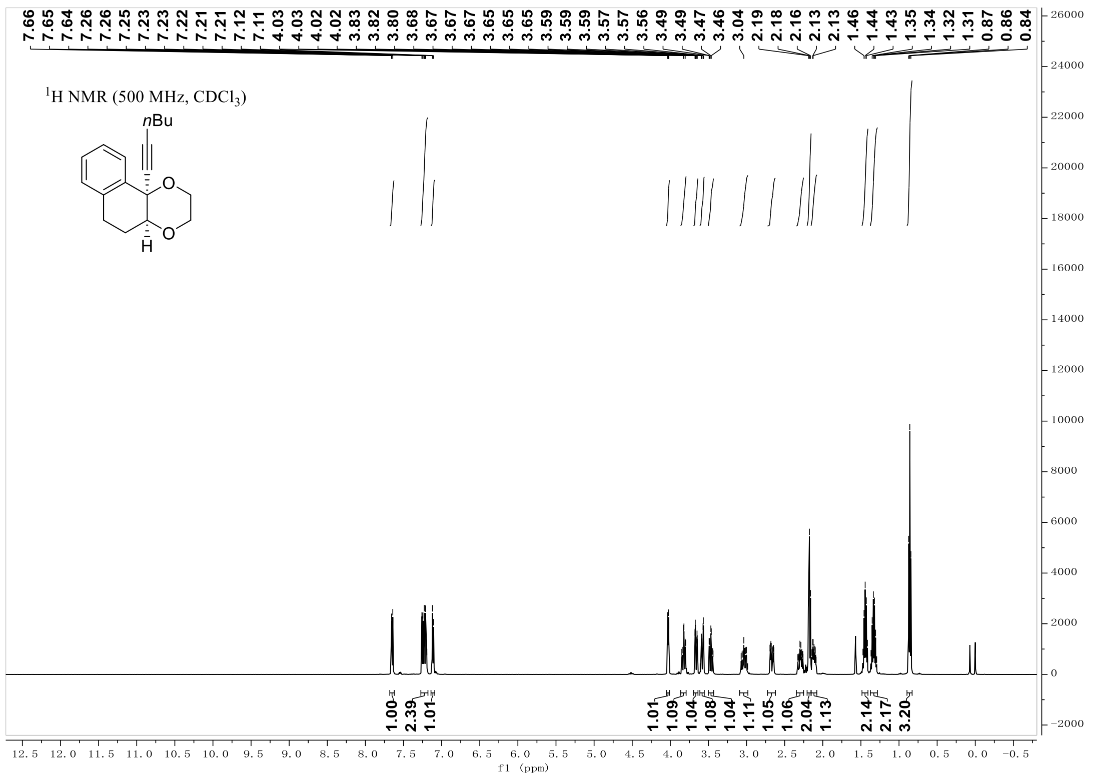

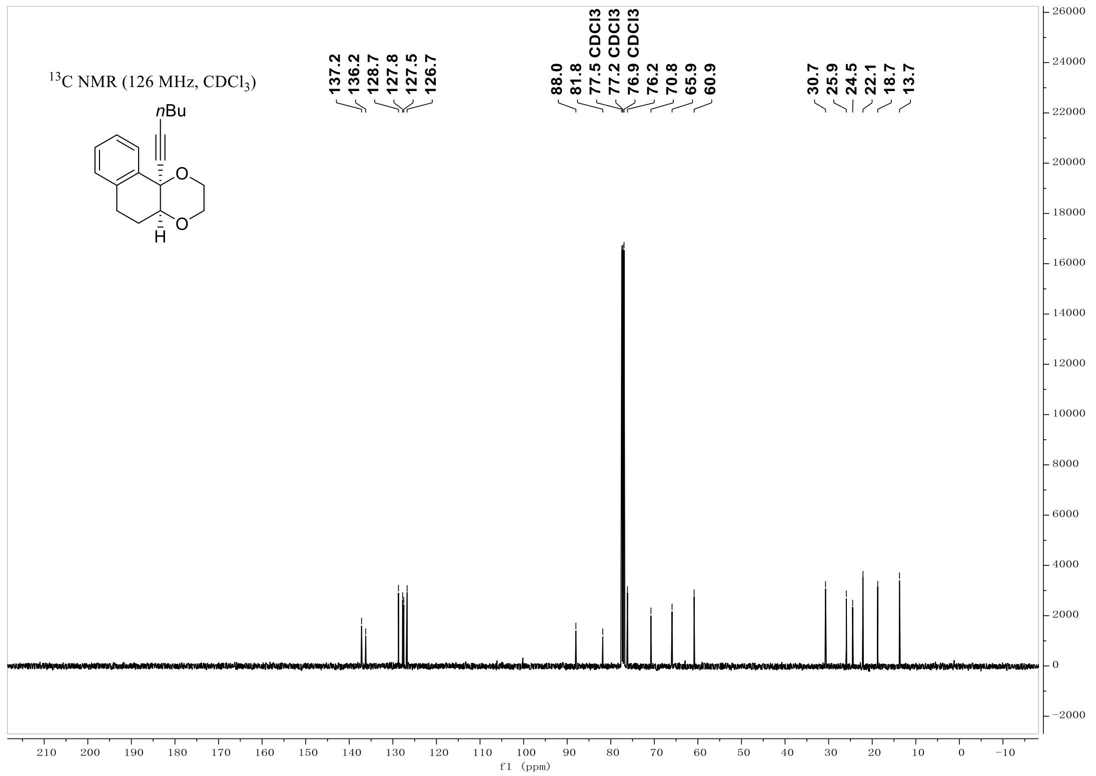

Supplementary Fig. 31. ^{1}H NMR and ^{13}C NMR spectra for compound 32

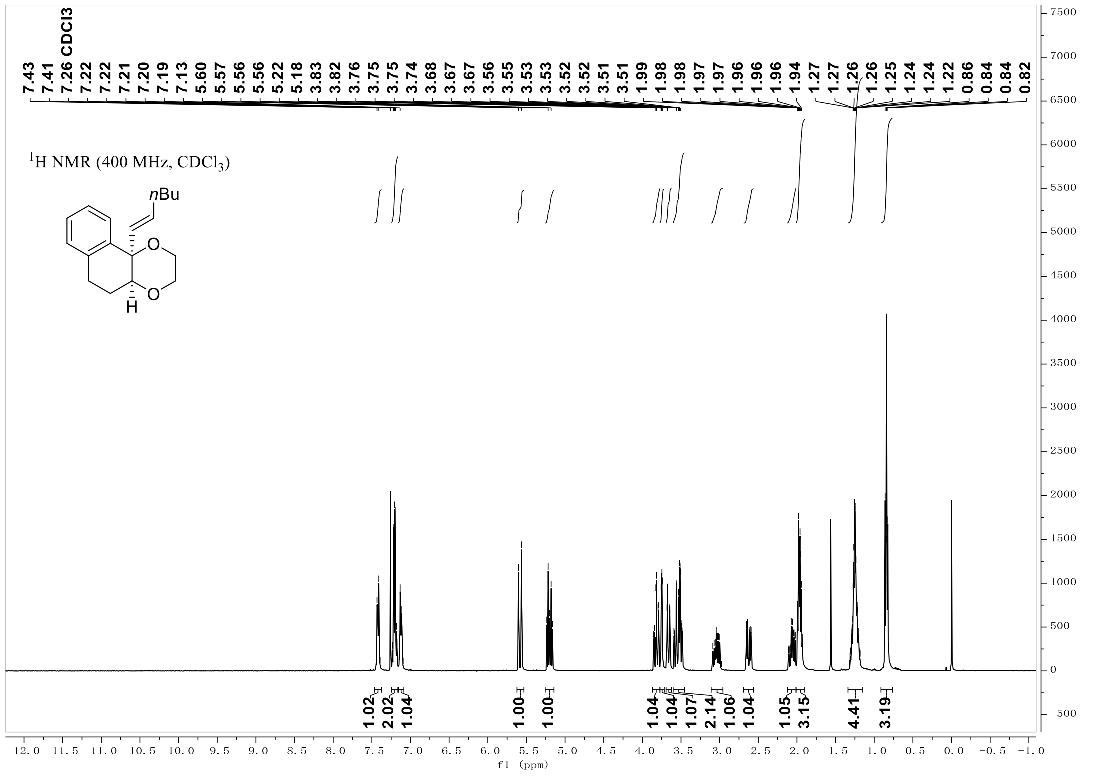

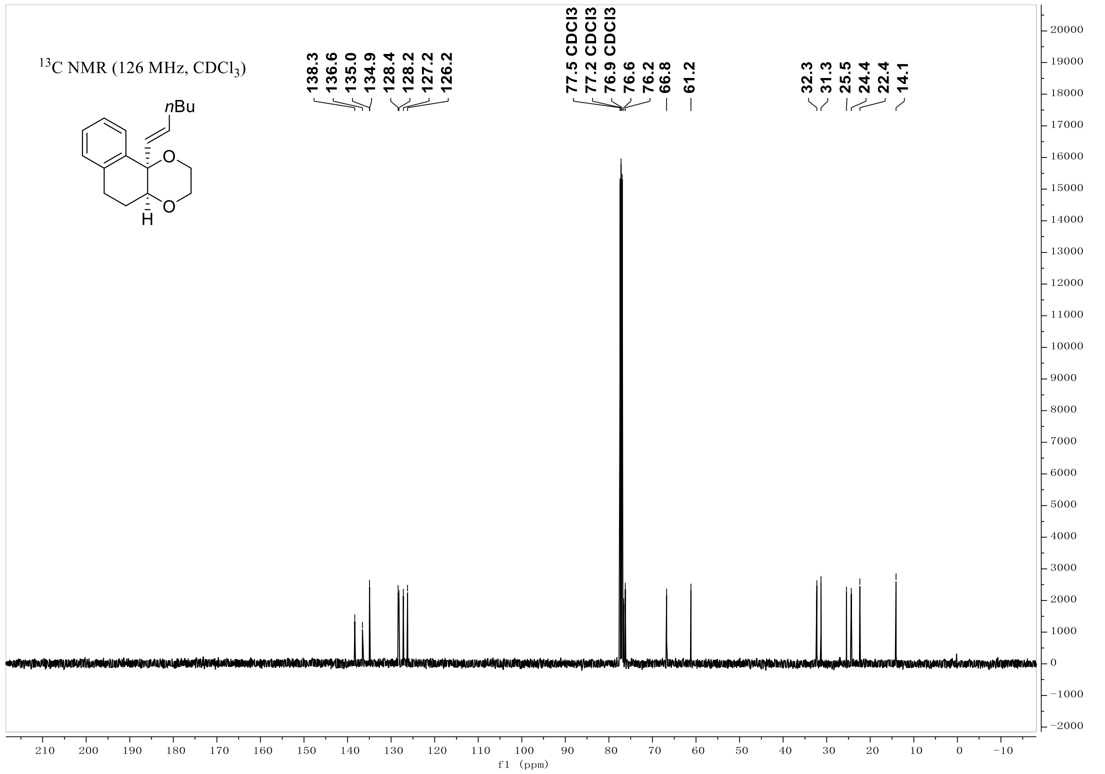

Supplementary Fig. 32. ^{1}H NMR and ^{13}C NMR compound 33

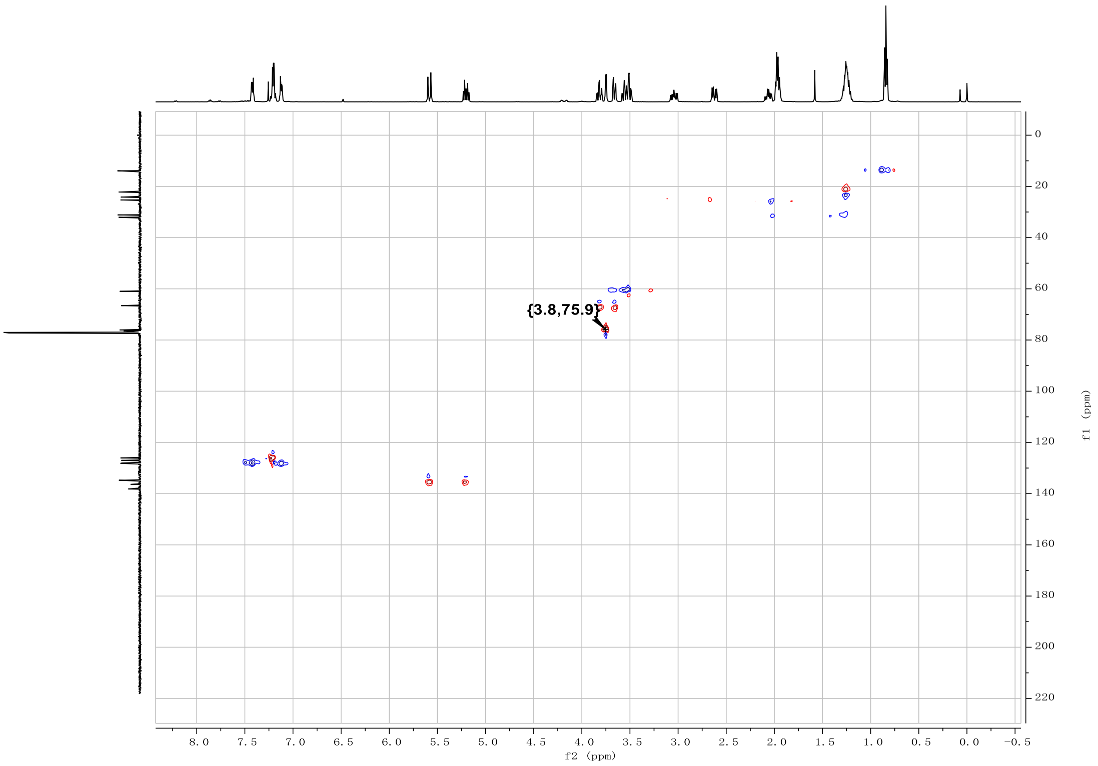

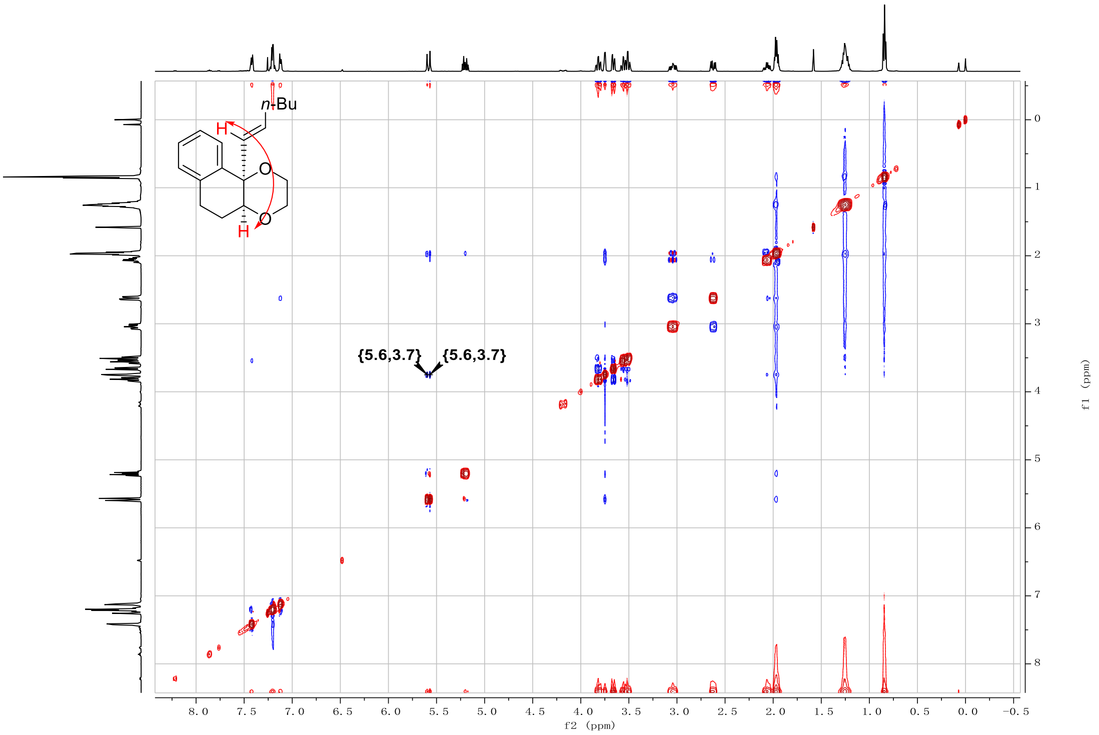

Supplementary Fig. 33. HSQC and NOE spectra for compound 33

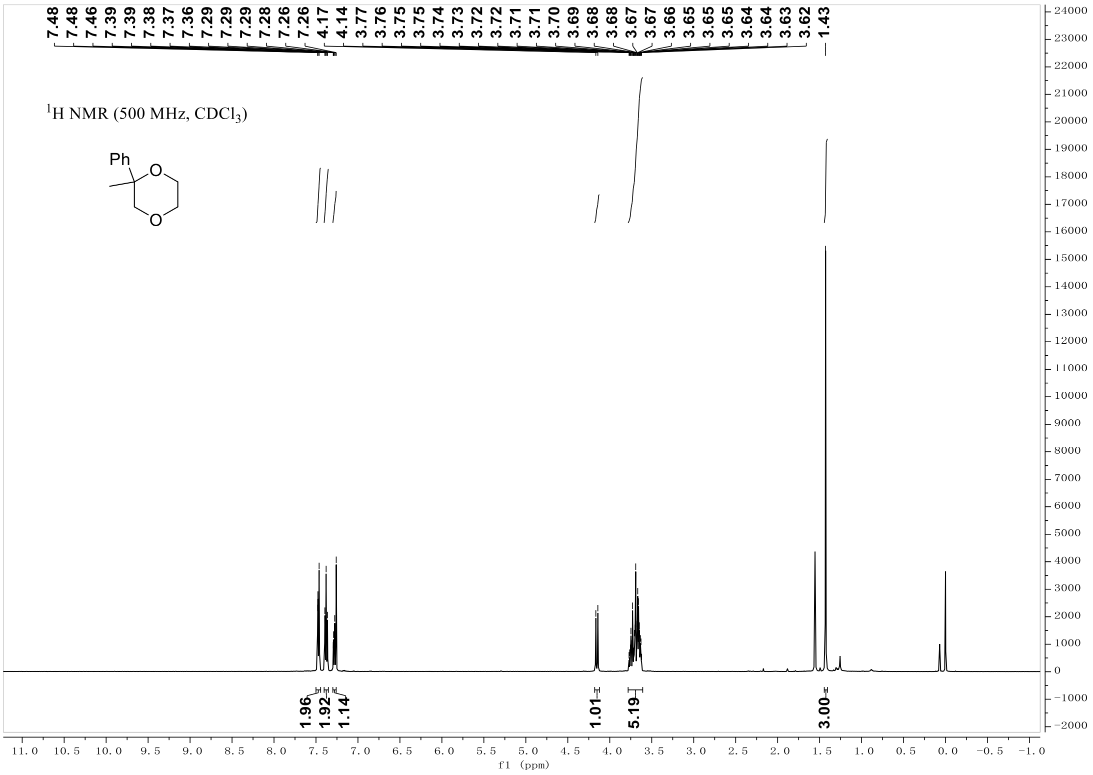

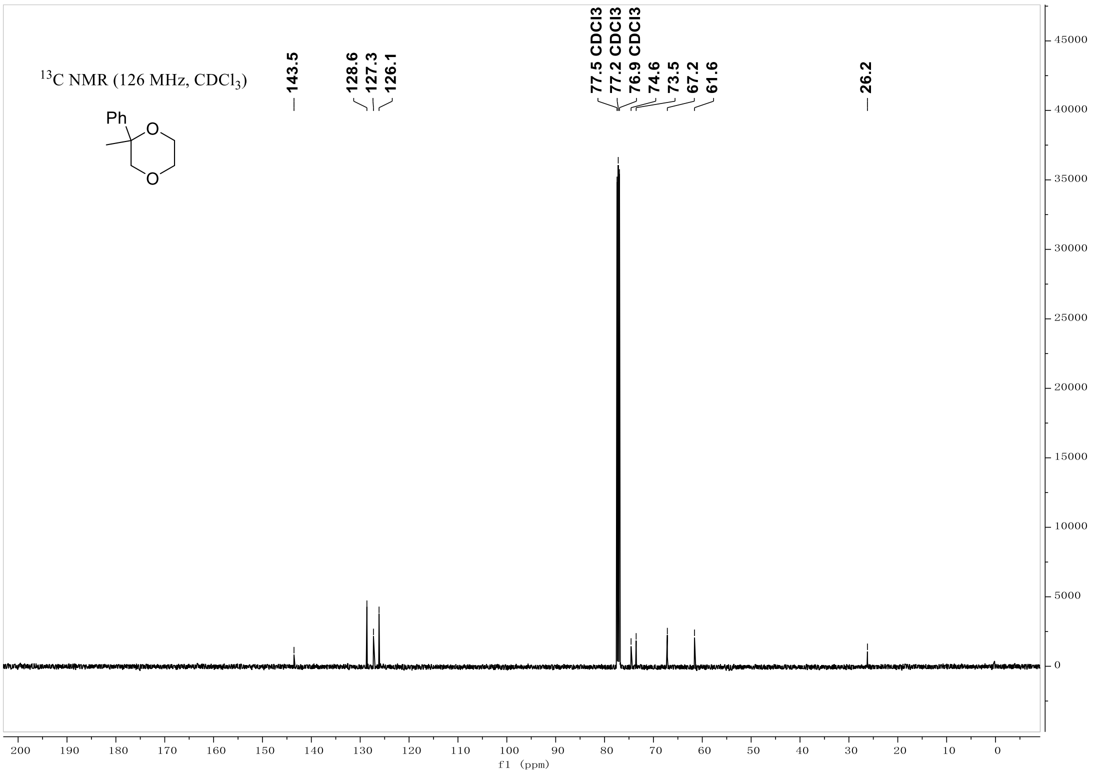

Supplementary Fig. 34. ¹H NMR and ¹³C NMR spectra for compound 34

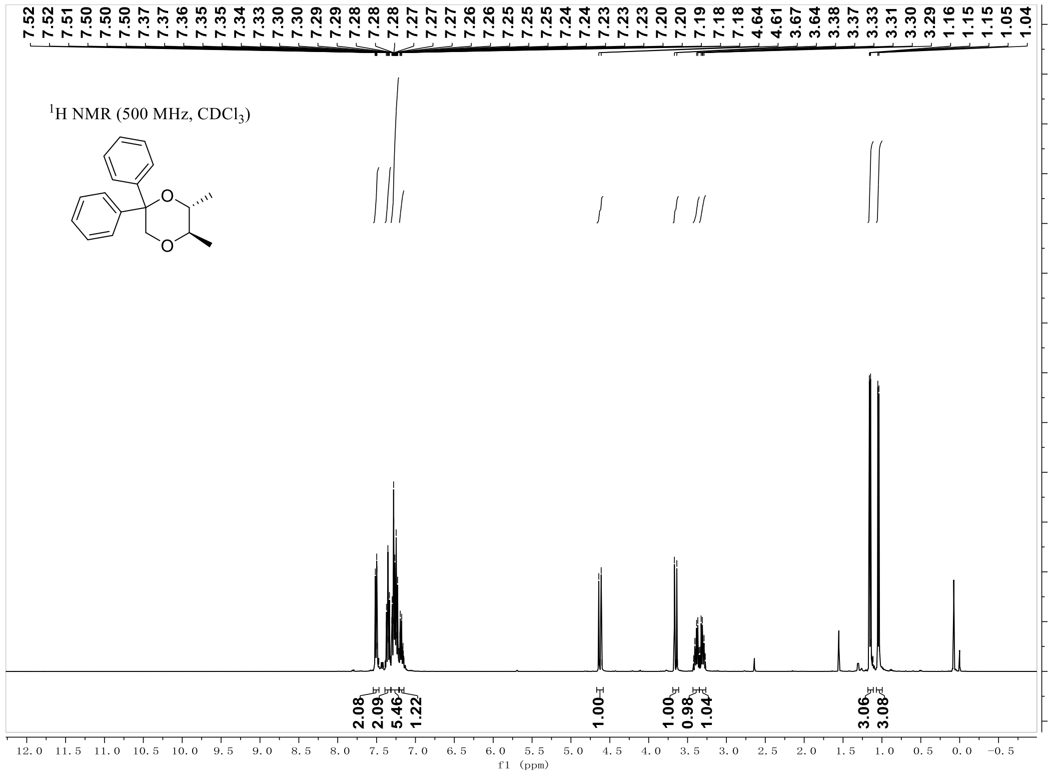

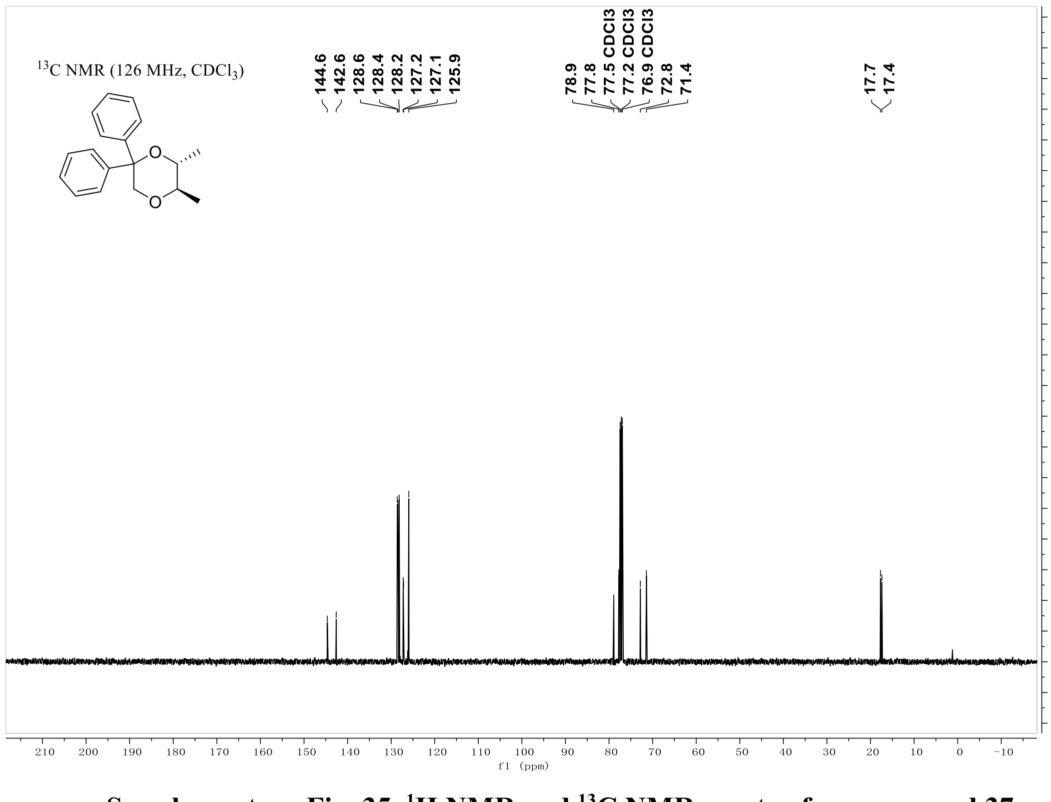

Supplementary Fig. 35. ^{1}H NMR and ^{13}C NMR spectra for compound 37

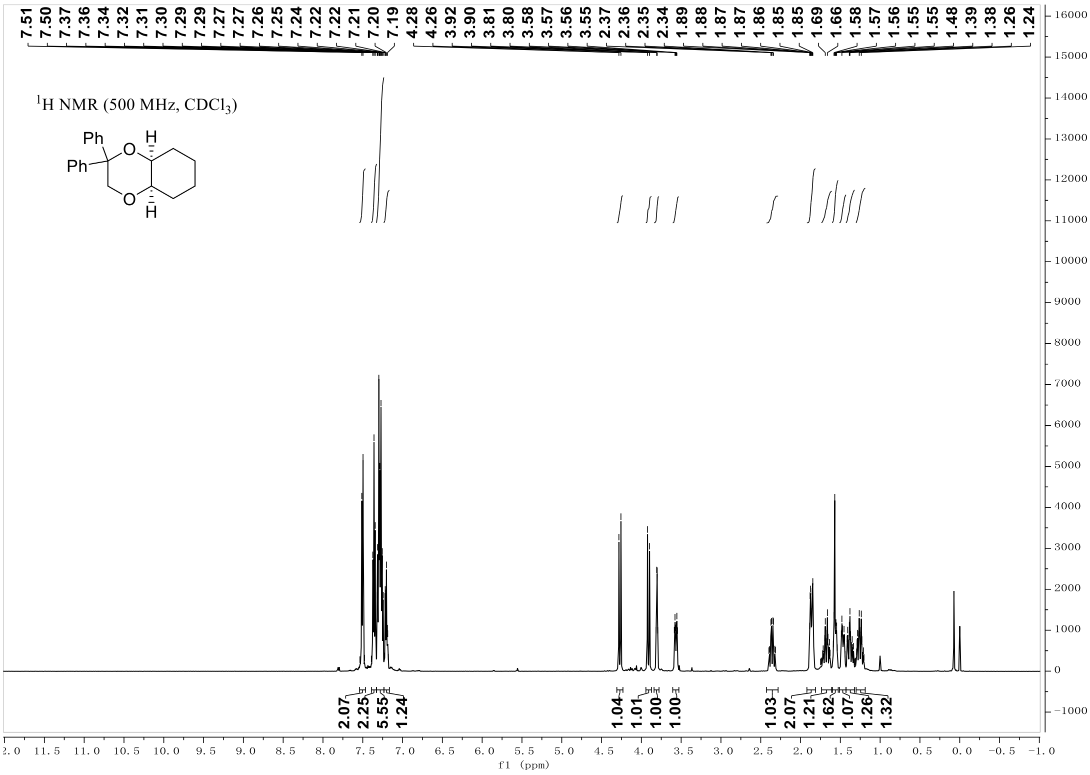

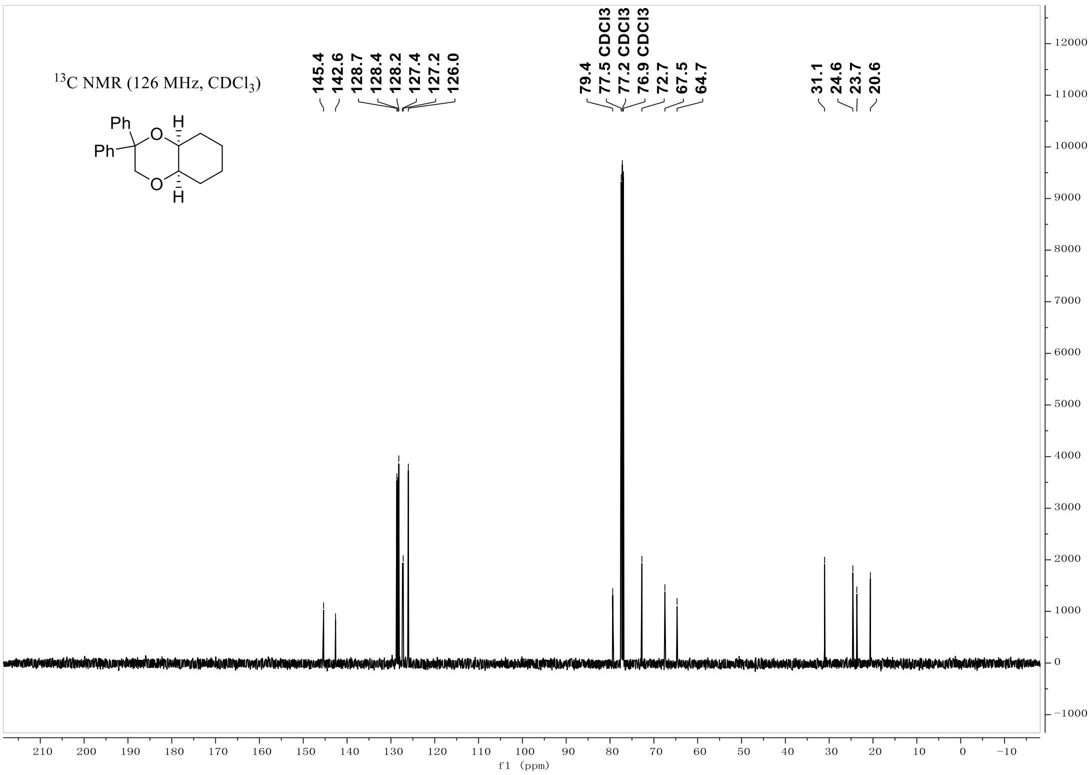

Supplementary Fig. 36. ^{1}H NMR and ^{13}C NMR spectra for compound 38

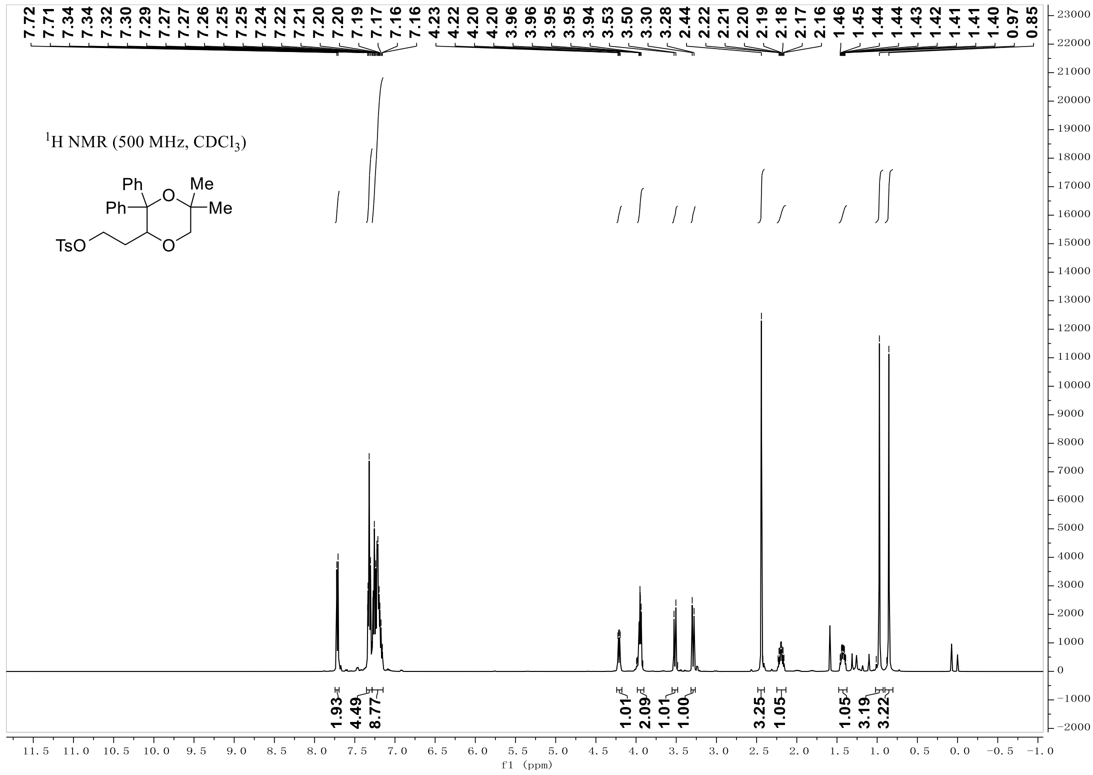

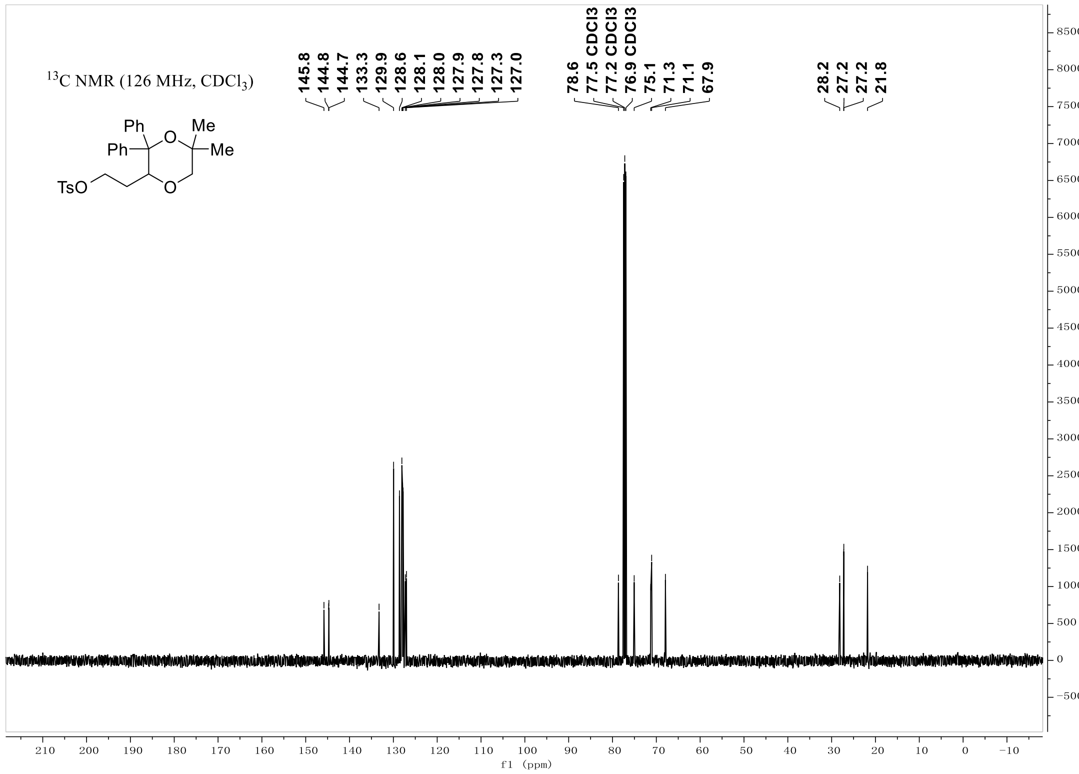

Supplementary Fig. 37. ^{1}H NMR and ^{13}C NMR spectra for compound 39

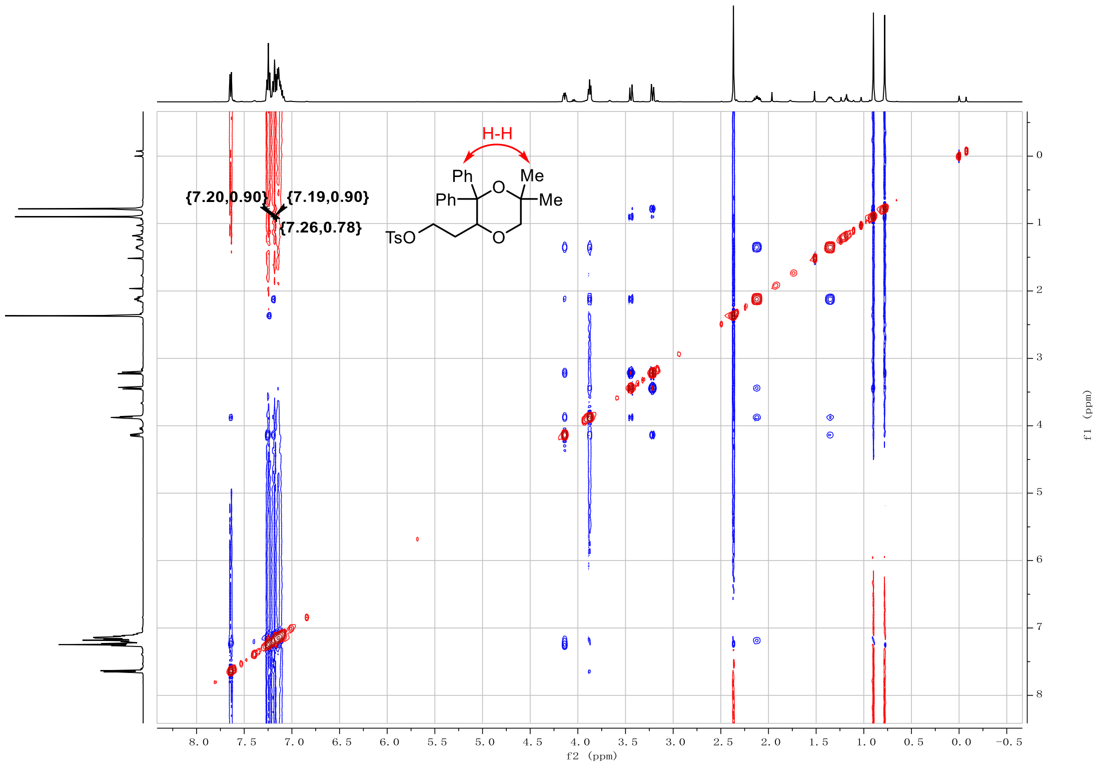

Supplementary Fig. 38. NOE spectra for compound 39

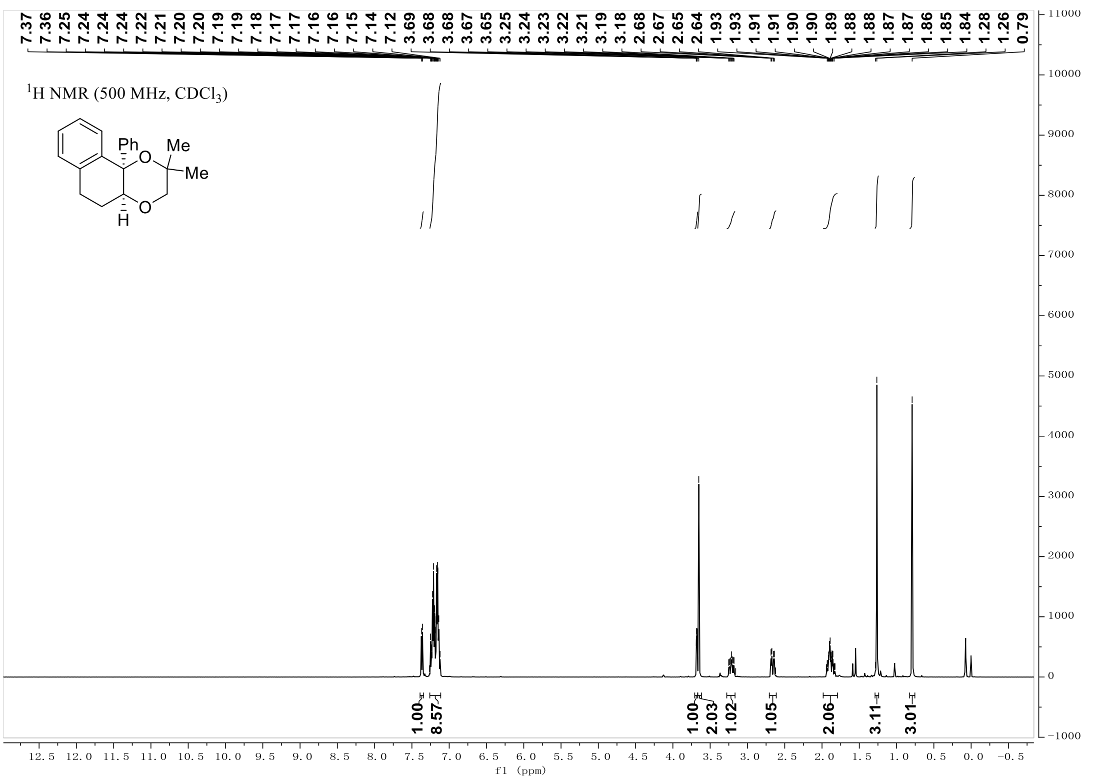

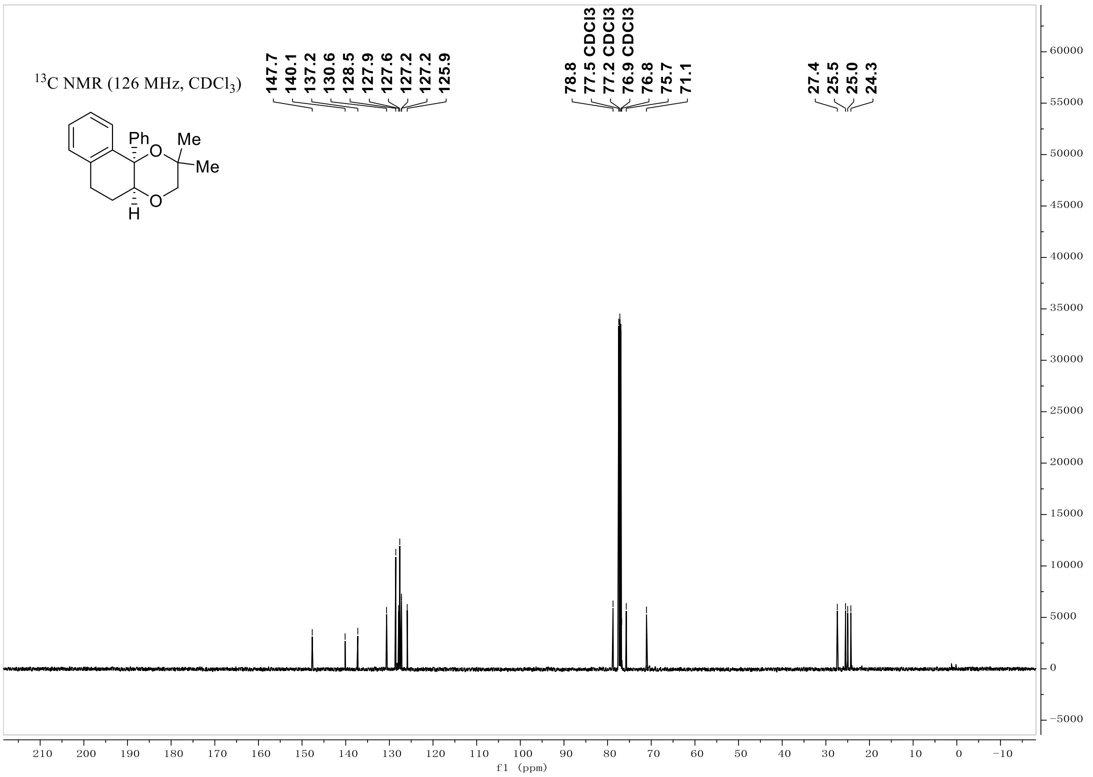

Supplementary Fig. 39. ^{1}H NMR and ^{13}C NMR spectra for compound 40

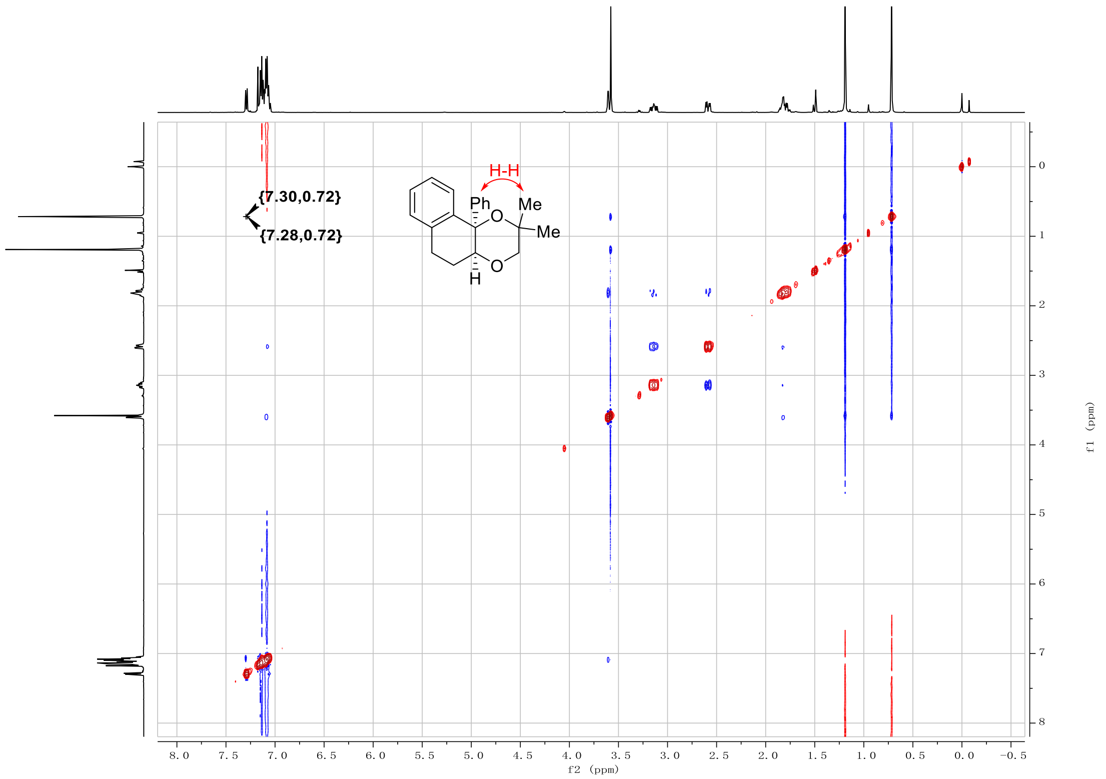

Supplementary Fig. 40. NOE spectra for compound 40

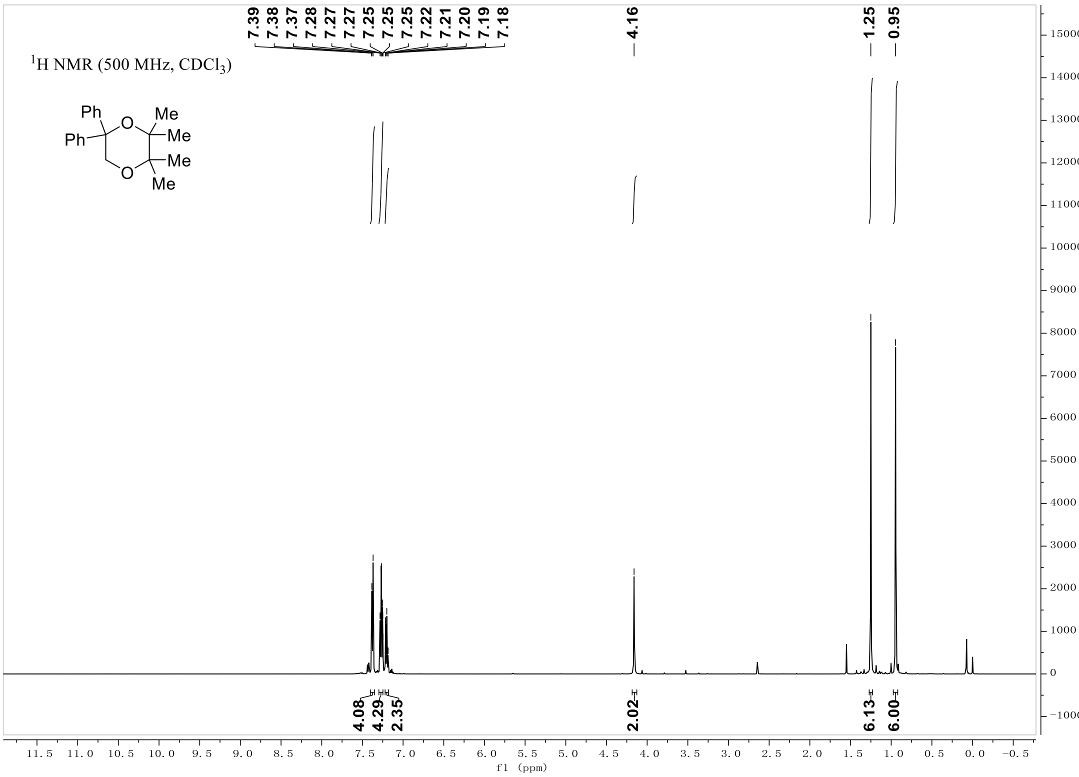

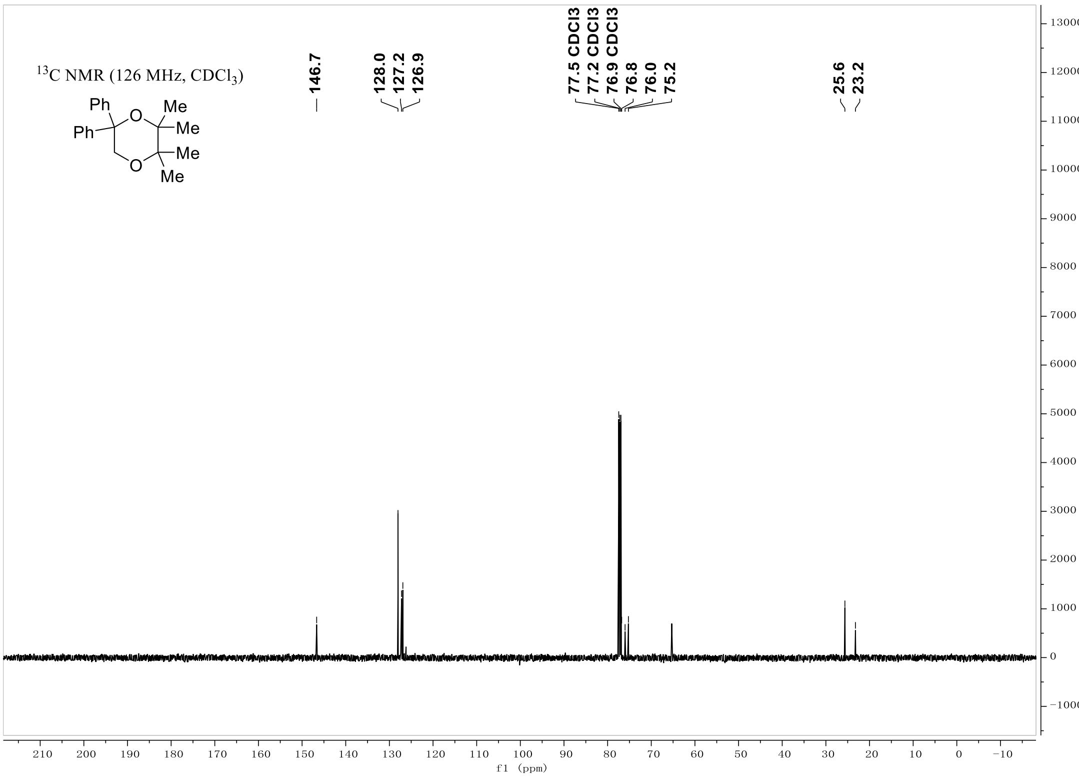

Supplementary Fig. 41. ^{1}H NMR and ^{13}C NMR spectra for compound 41

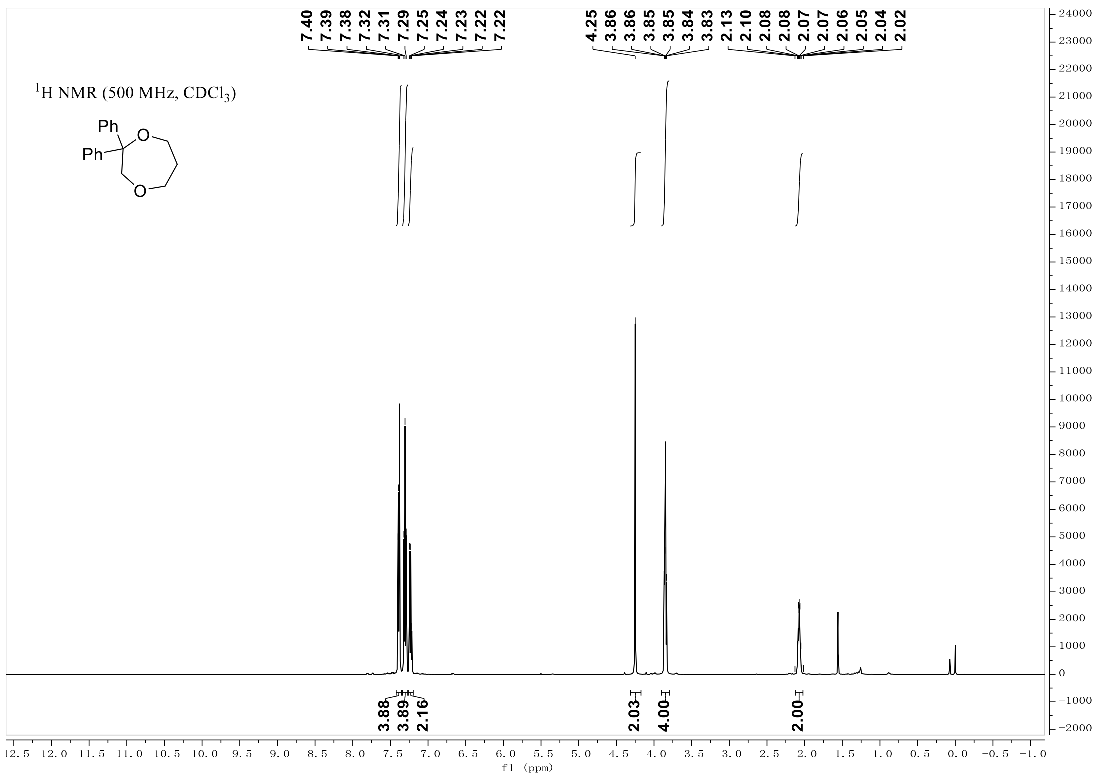

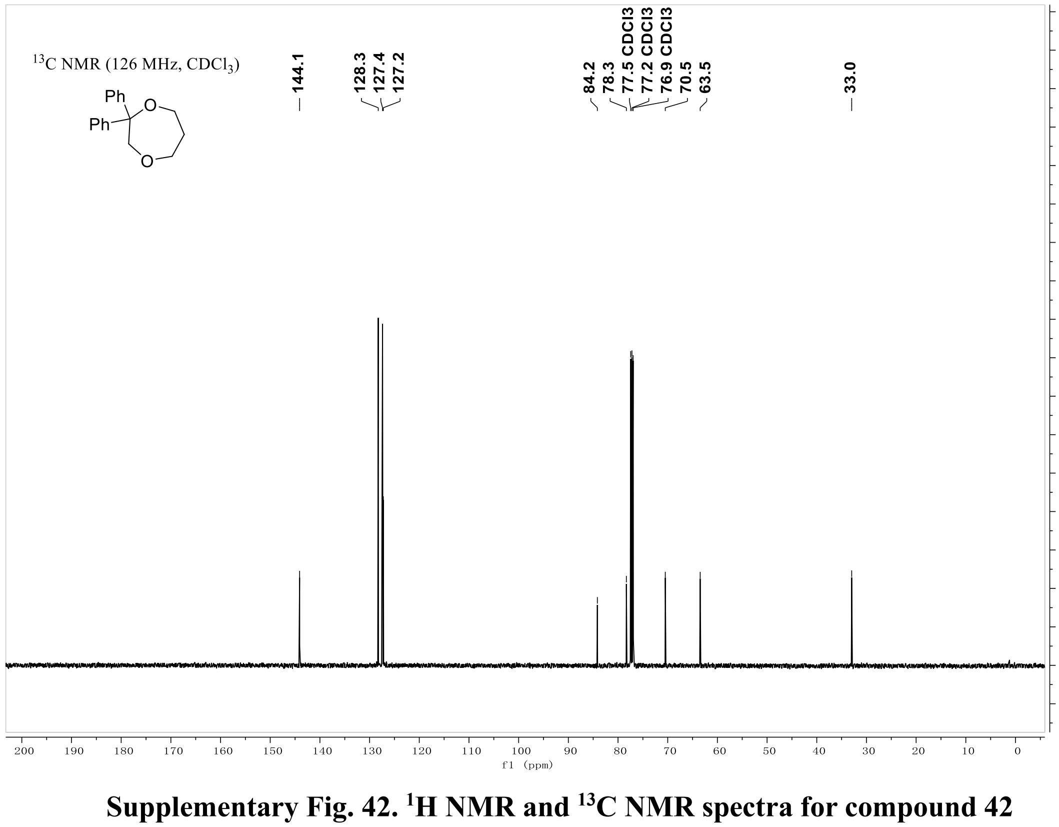

Supplementary Fig. 42. ^{1}H NMR and ^{13}C NMR spectra for compound 42

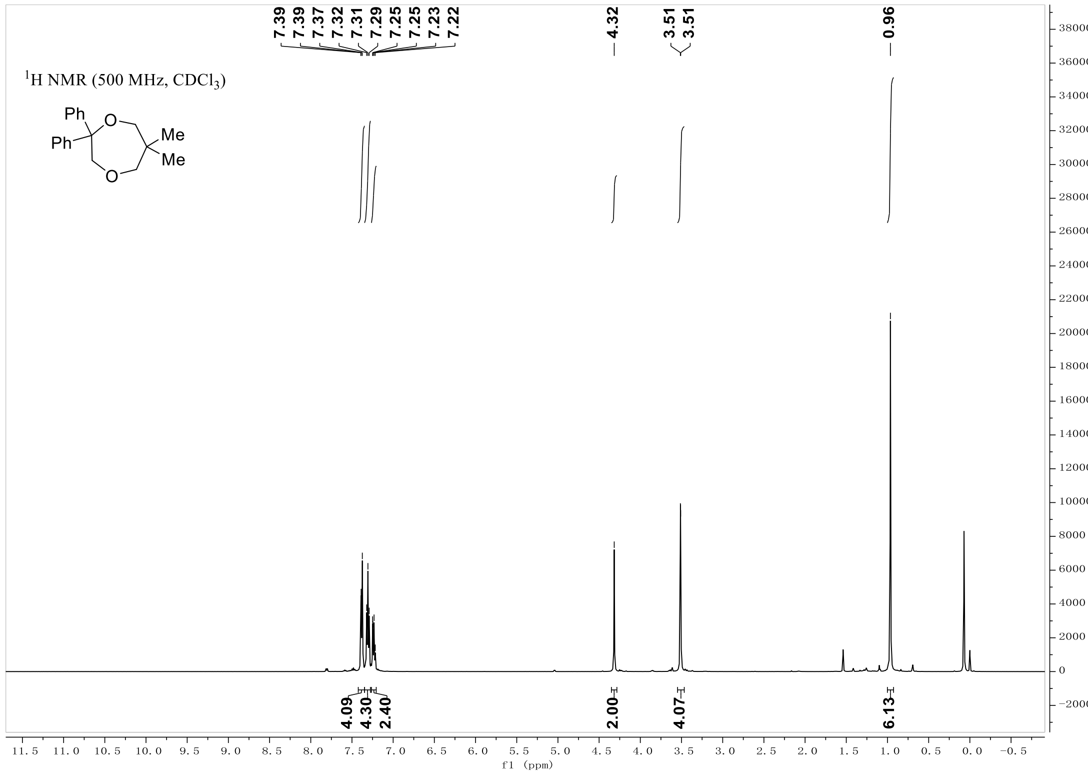

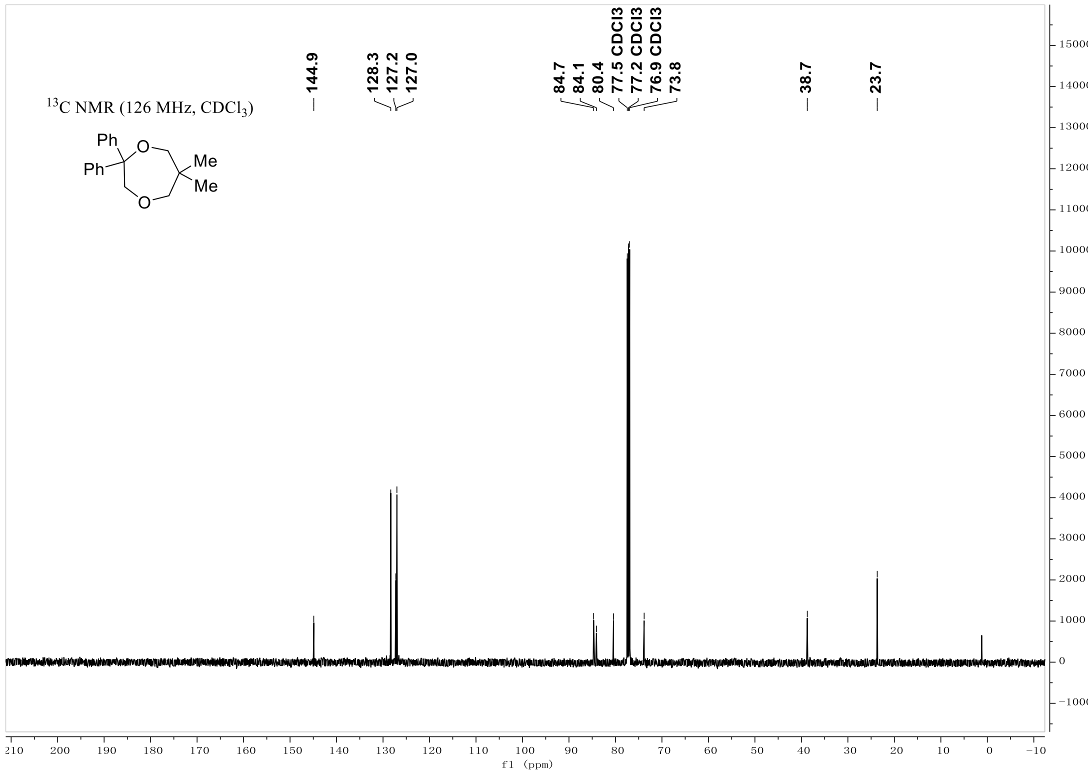

Supplementary Fig. 43. ^{1}H NMR and ^{13}C NMR spectra for compound 43

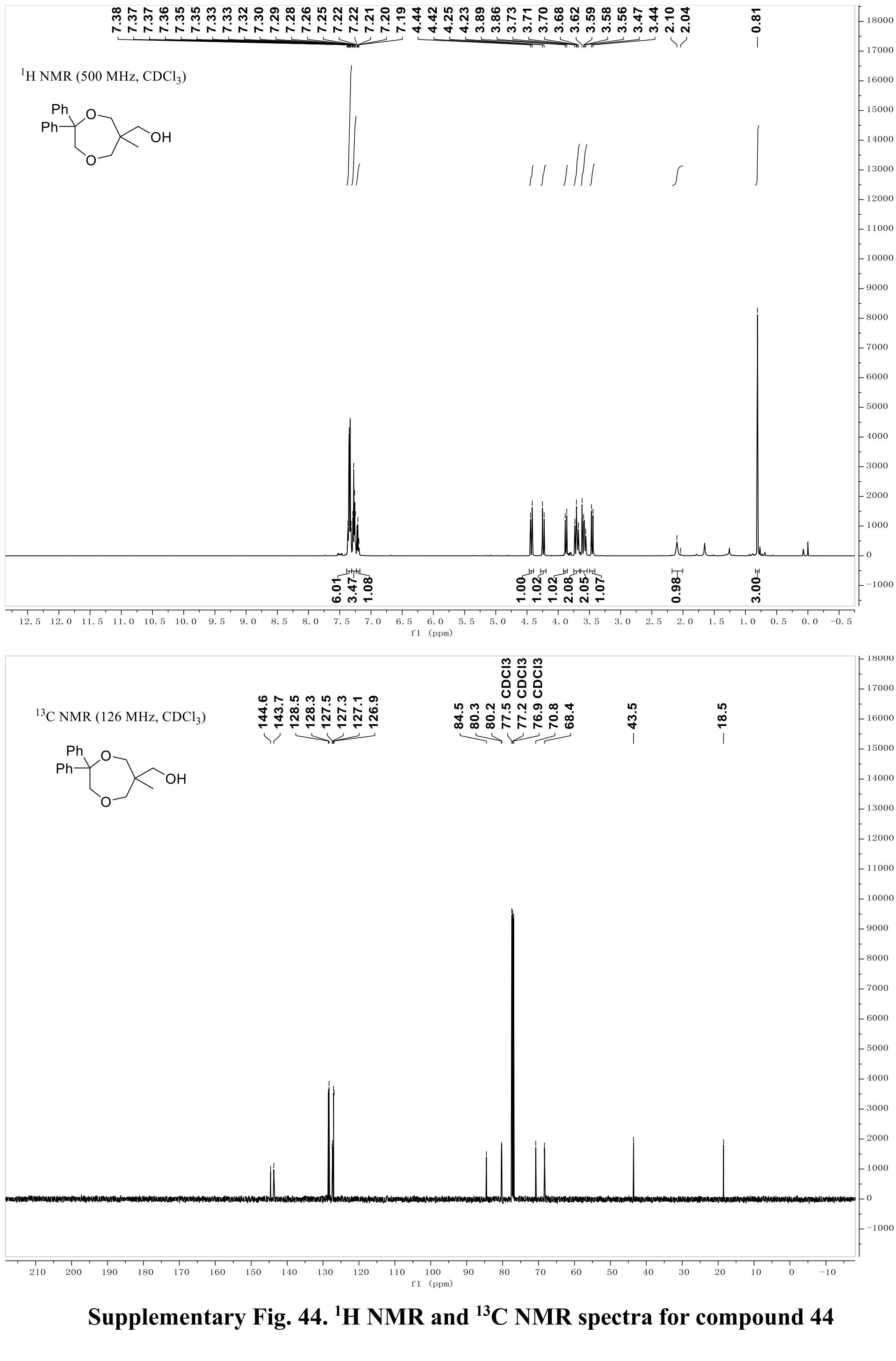

Supplementary Fig. 44. ^{1}H NMR and ^{13}C NMR spectra for compound 44

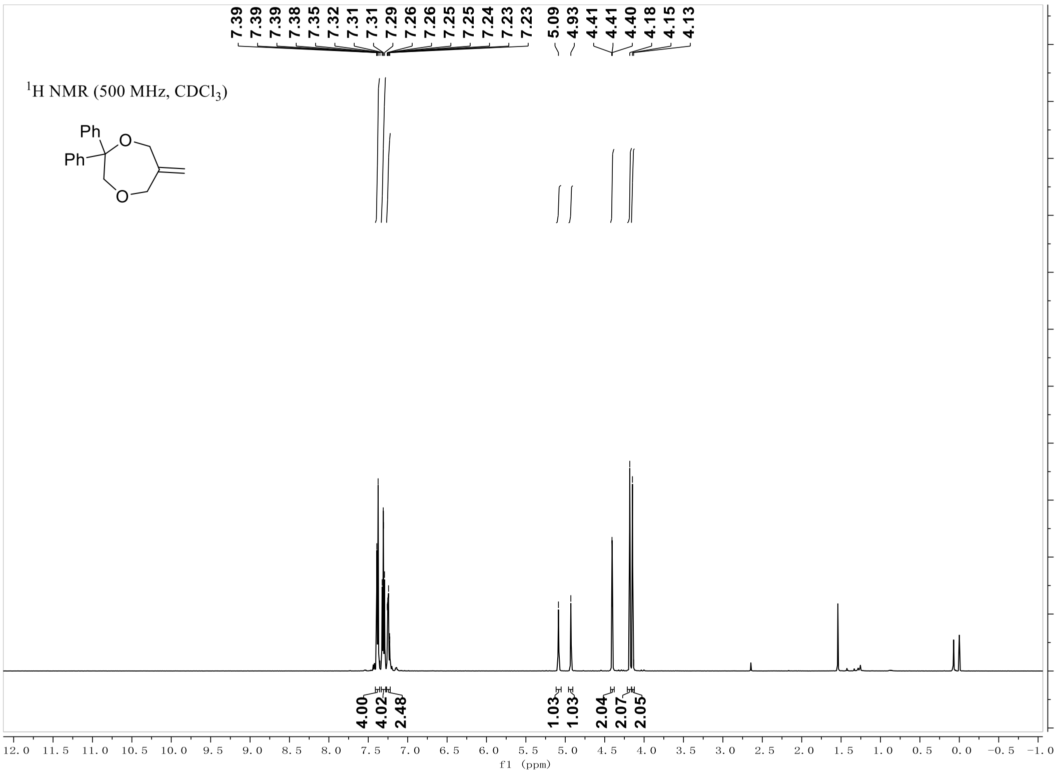

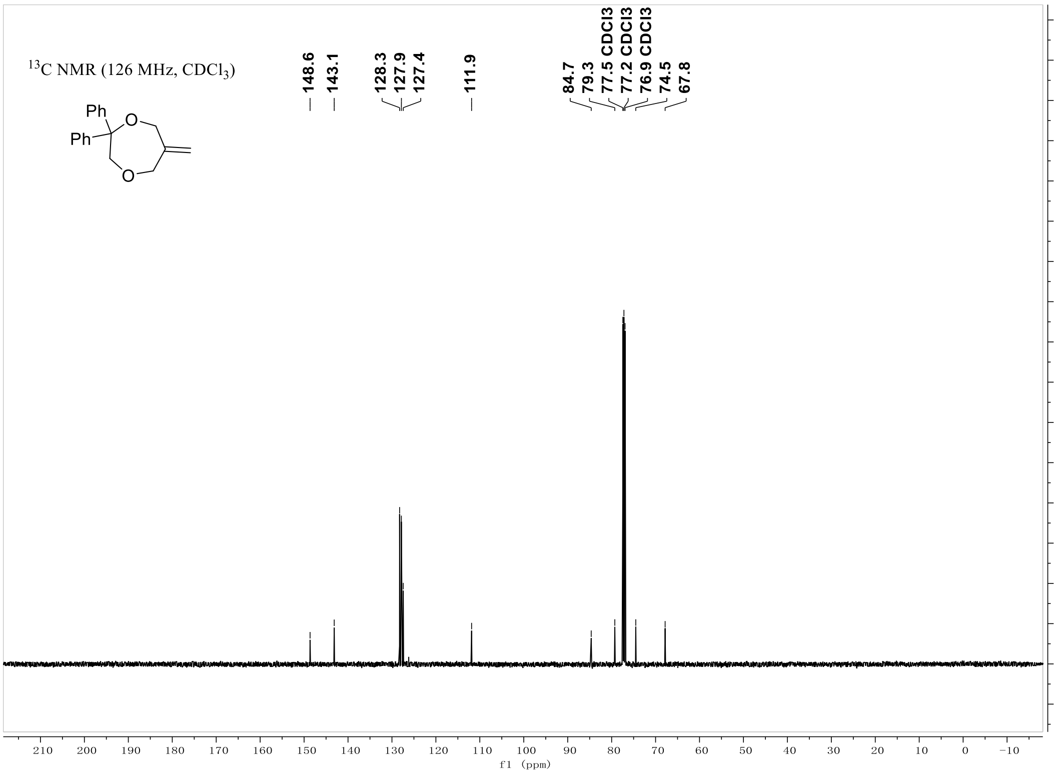

Supplementary Fig. 45. ^{1}H NMR and ^{13}C NMR spectra for compound 45

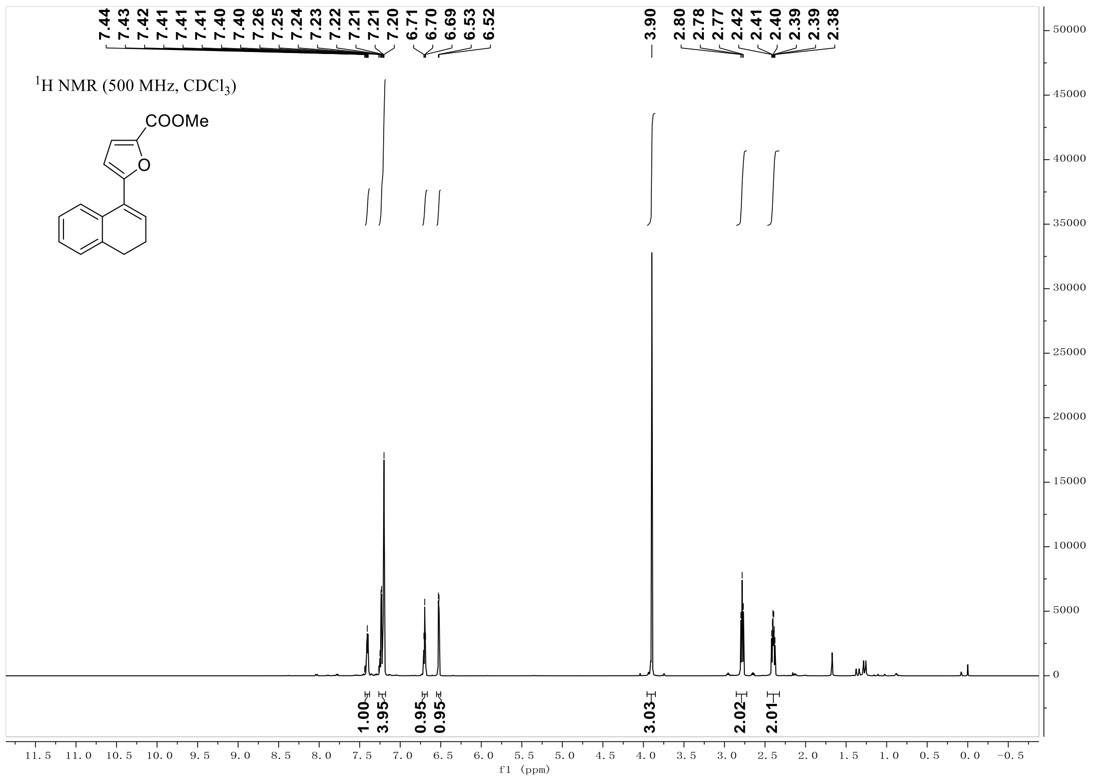

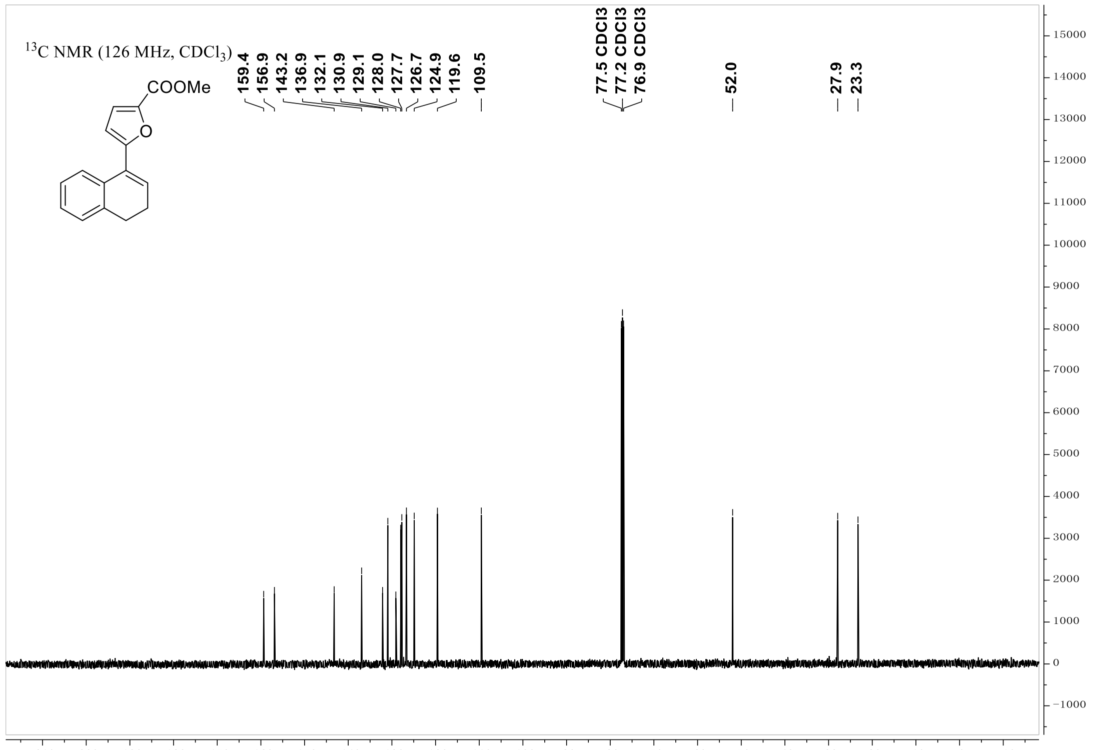

Supplementary Fig. 46. ^{1}H NMR and ^{13}C NMR spectra for compound S3

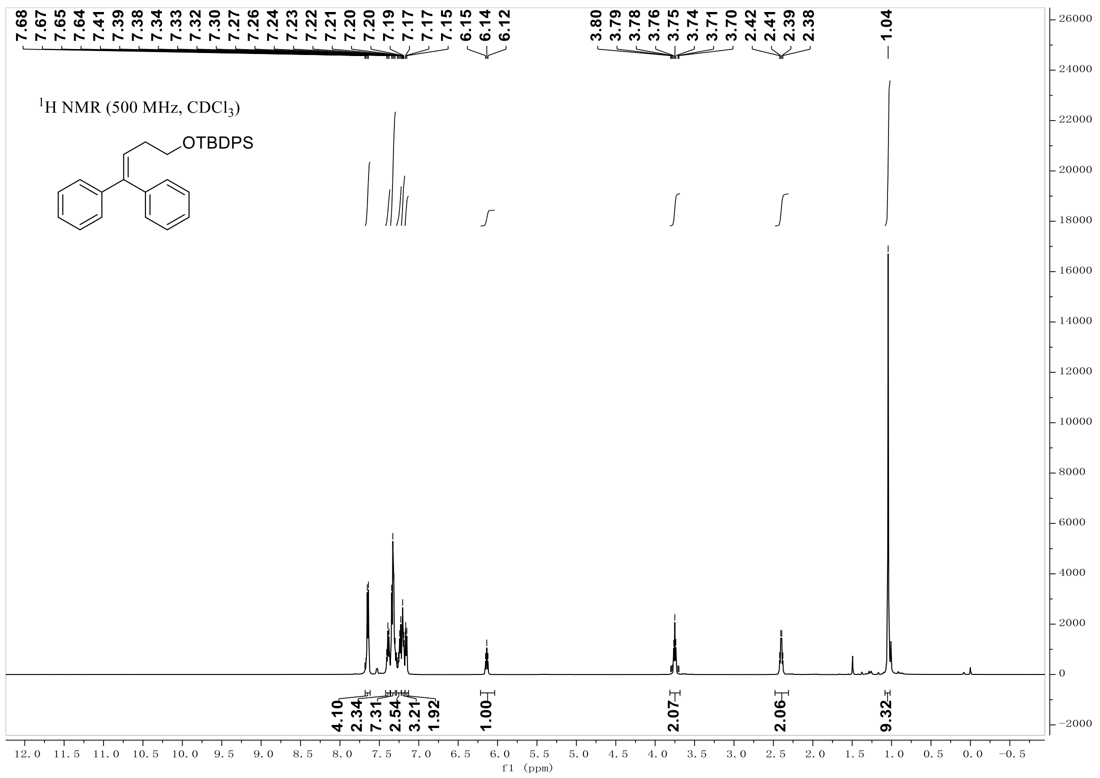

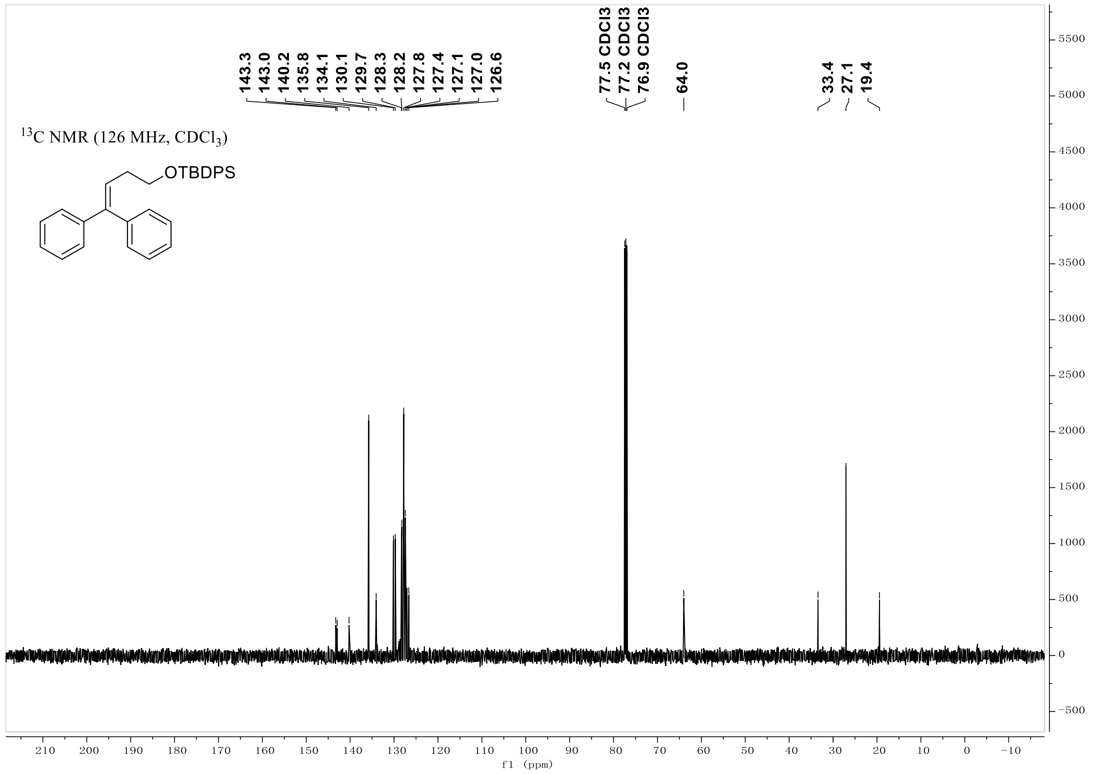

Supplementary Fig. 47. ^{1}H NMR and ^{13}C NMR spectra for compound S5

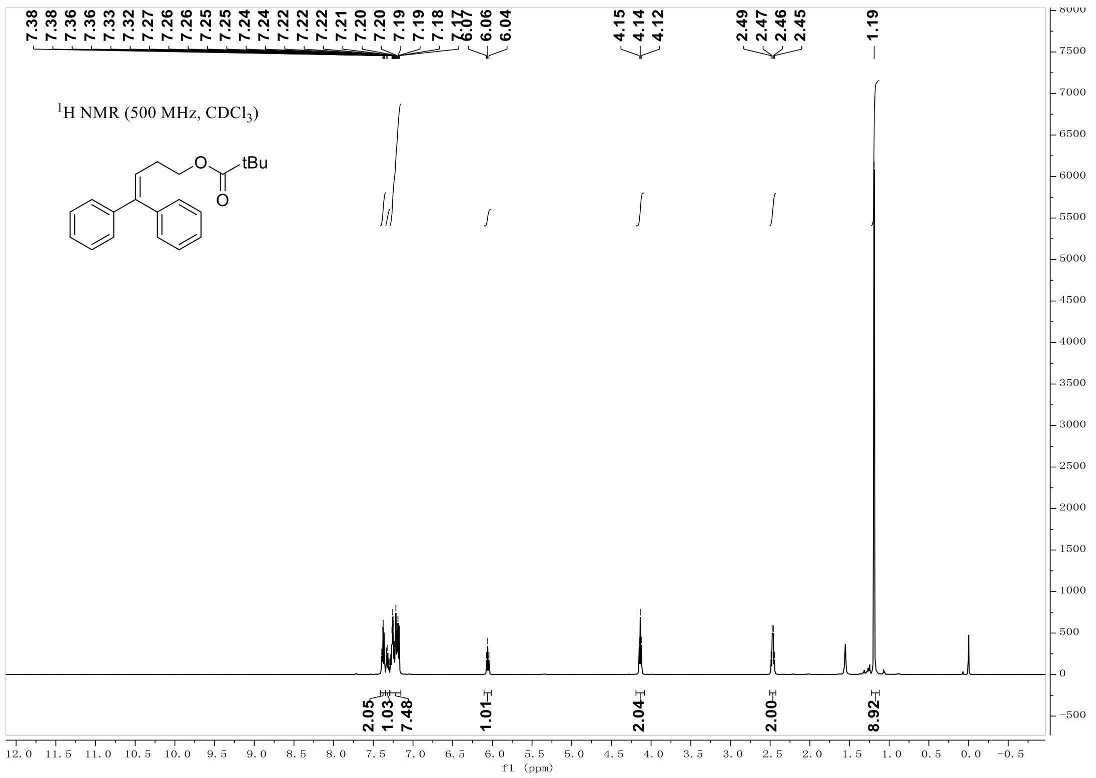

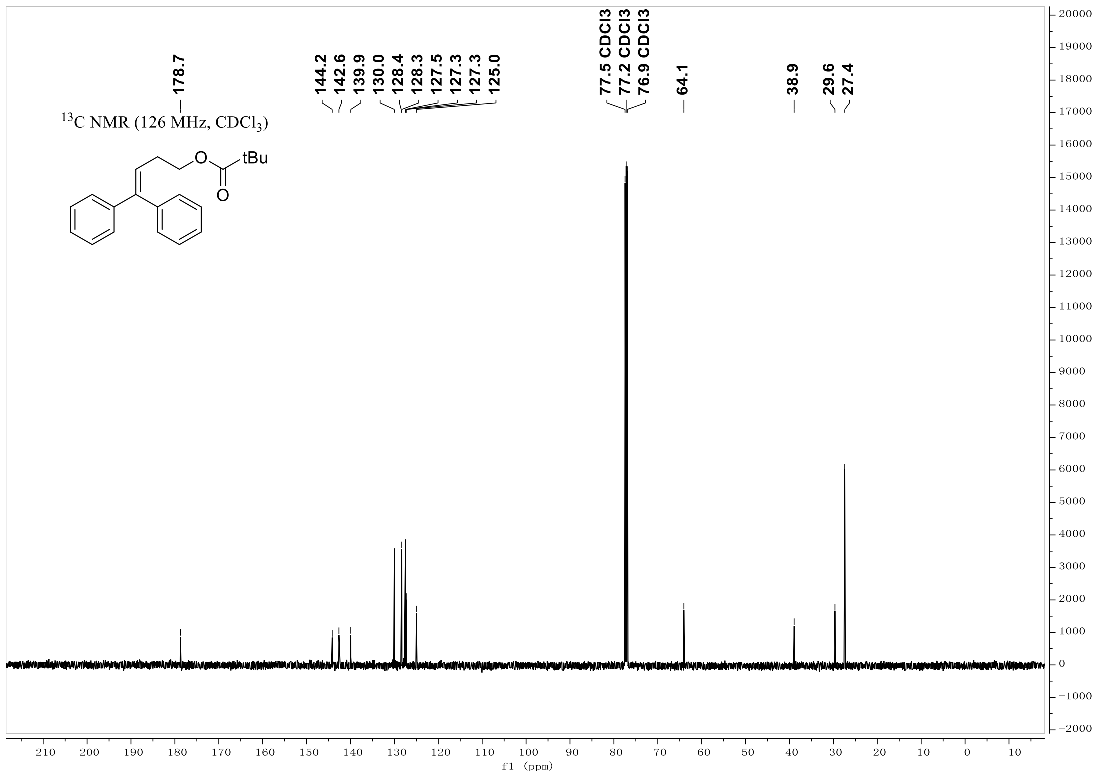

Supplementary Fig. 48. ^{1}H NMR and ^{13}C NMR spectra for compound S7

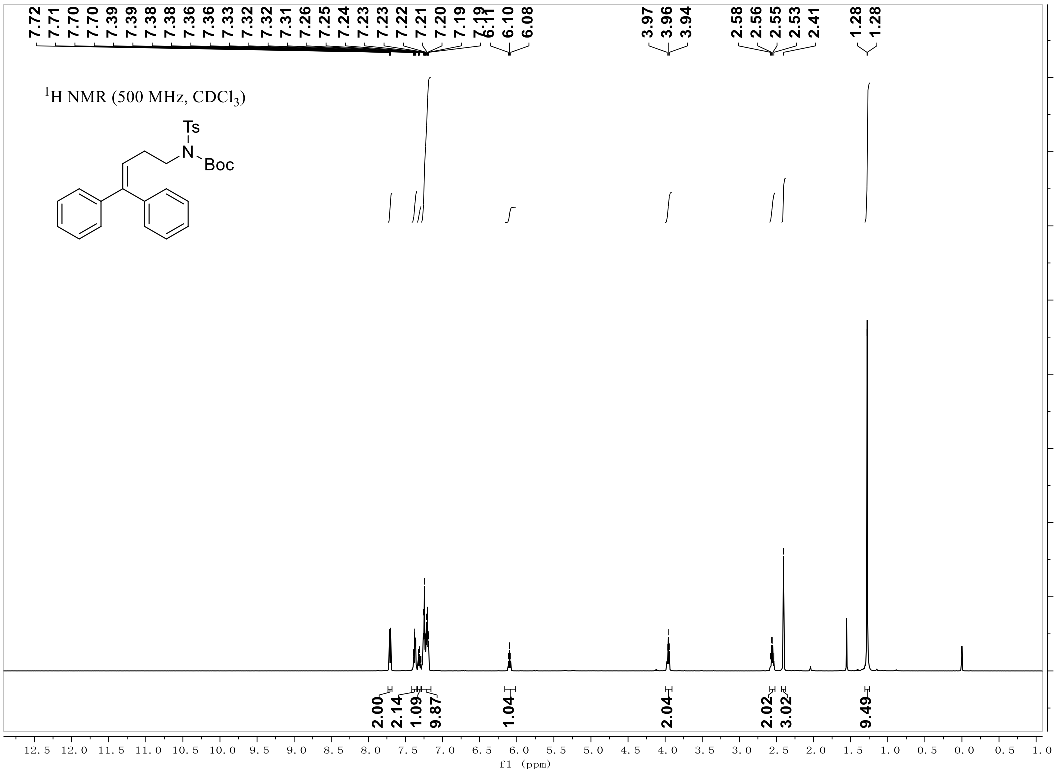

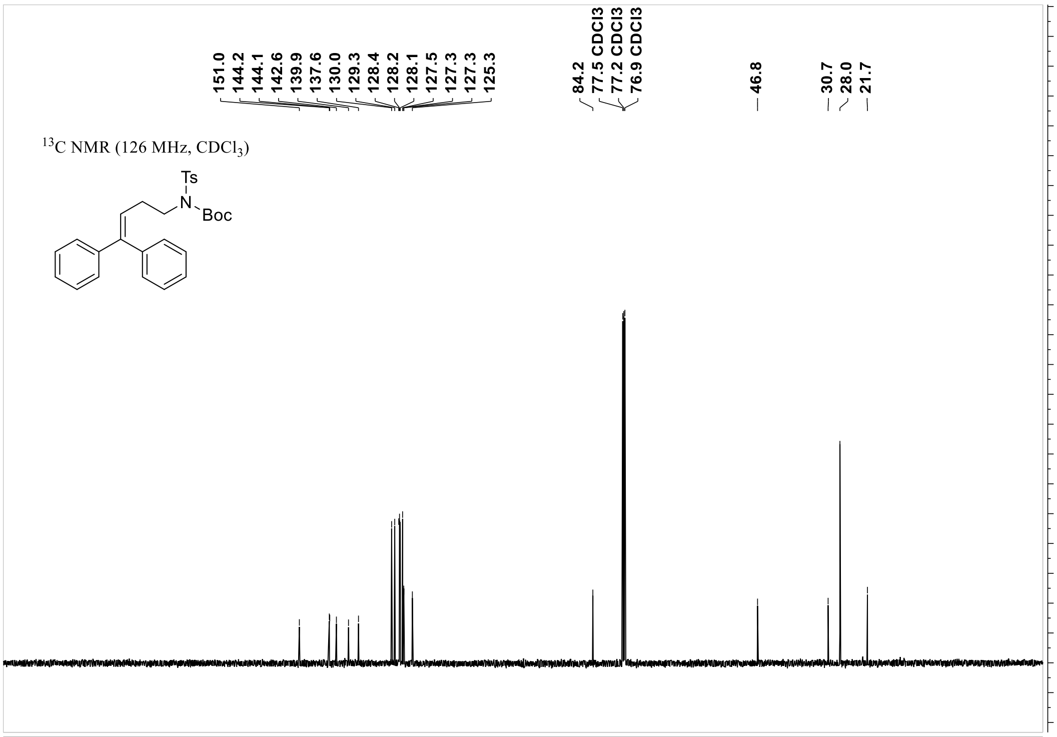

Supplementary Fig. 49. ^{1}H NMR and ^{13}C NMR spectra for compound S8

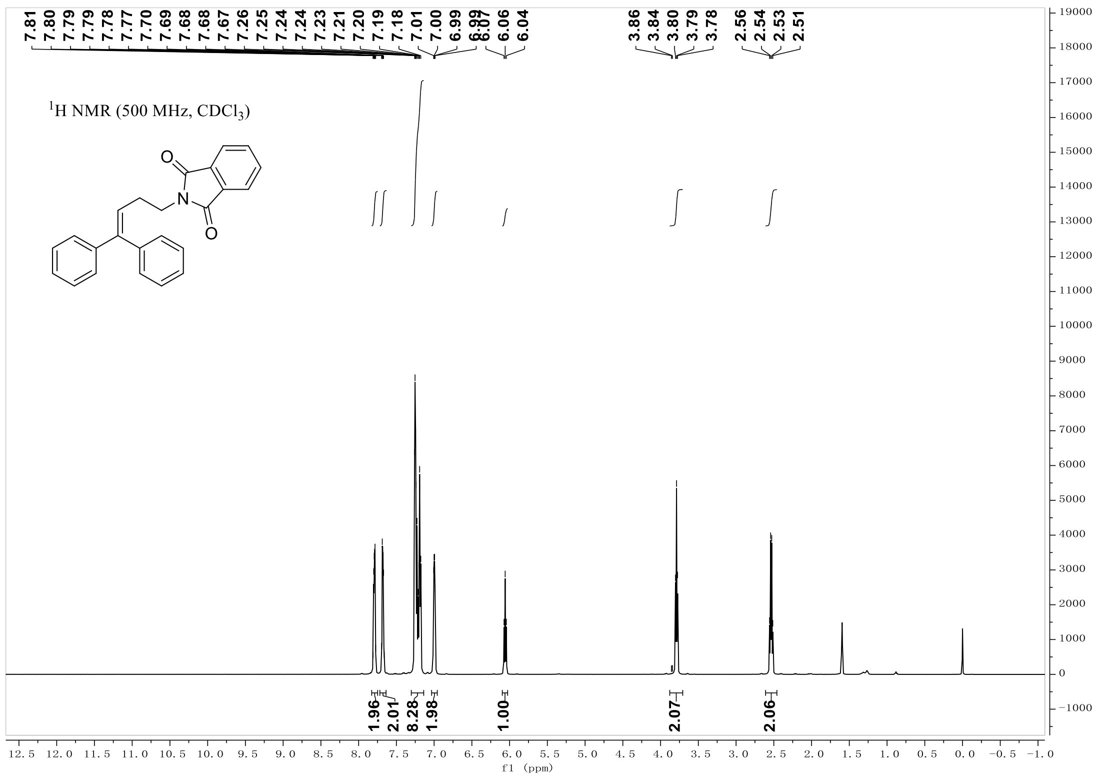

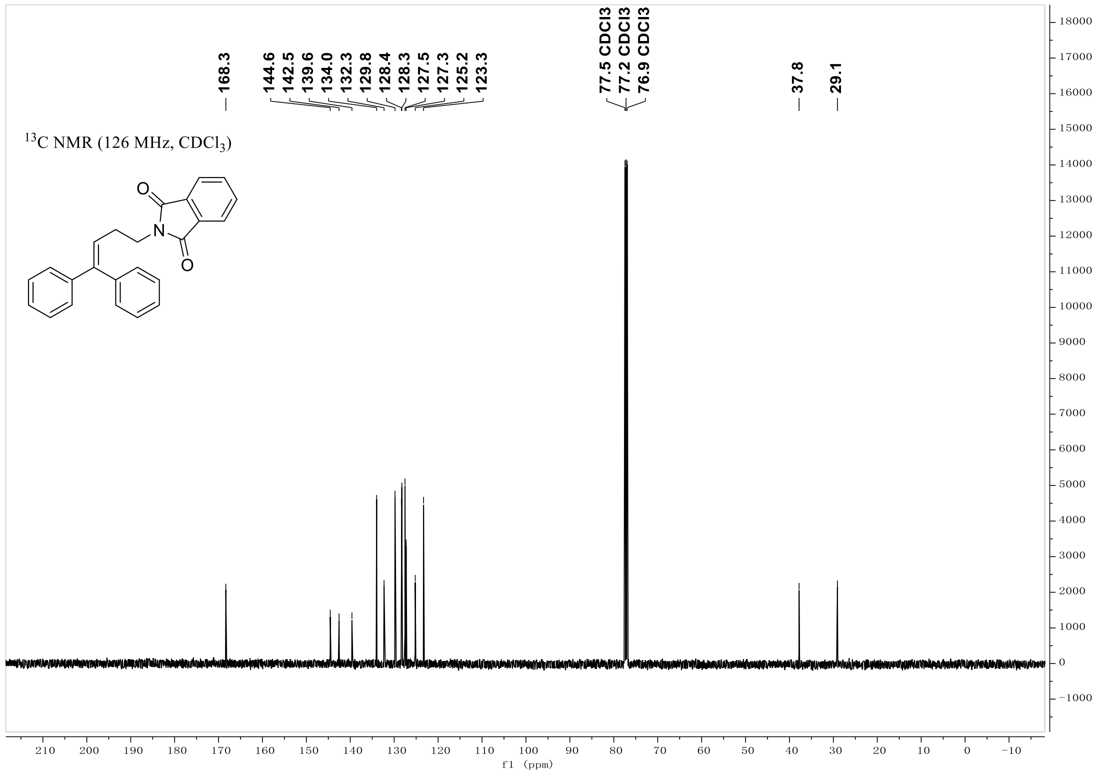

Supplementary Fig. 50. ^{1}H NMR and ^{13}C NMR spectra for compound S9

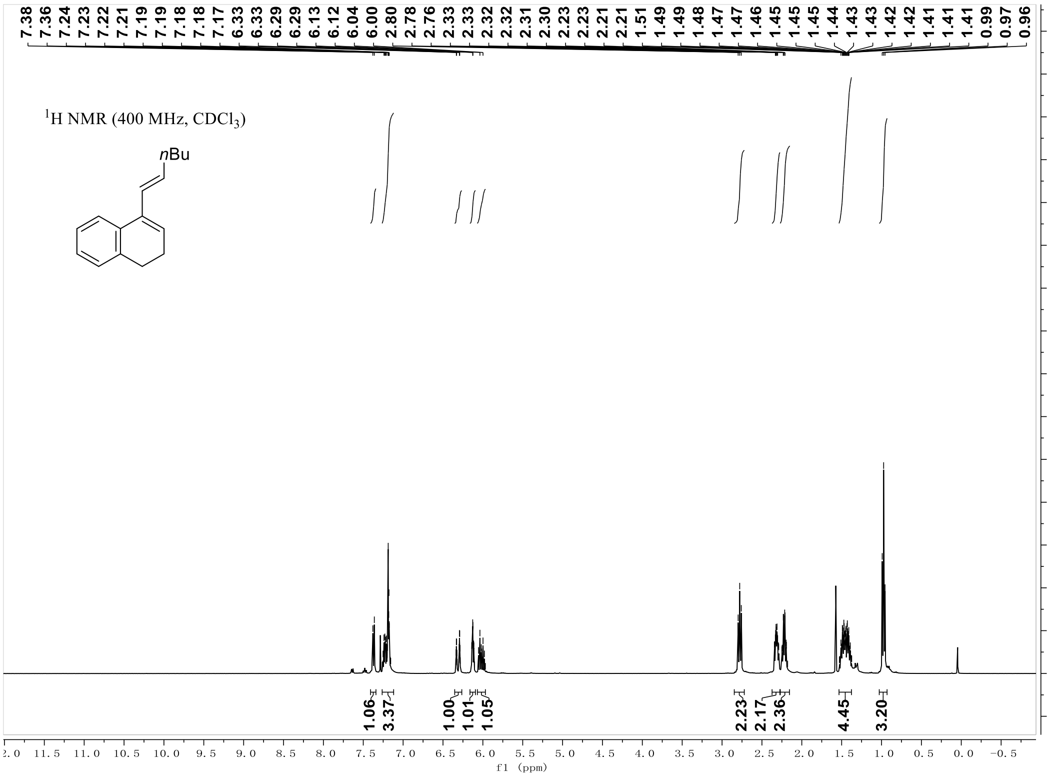

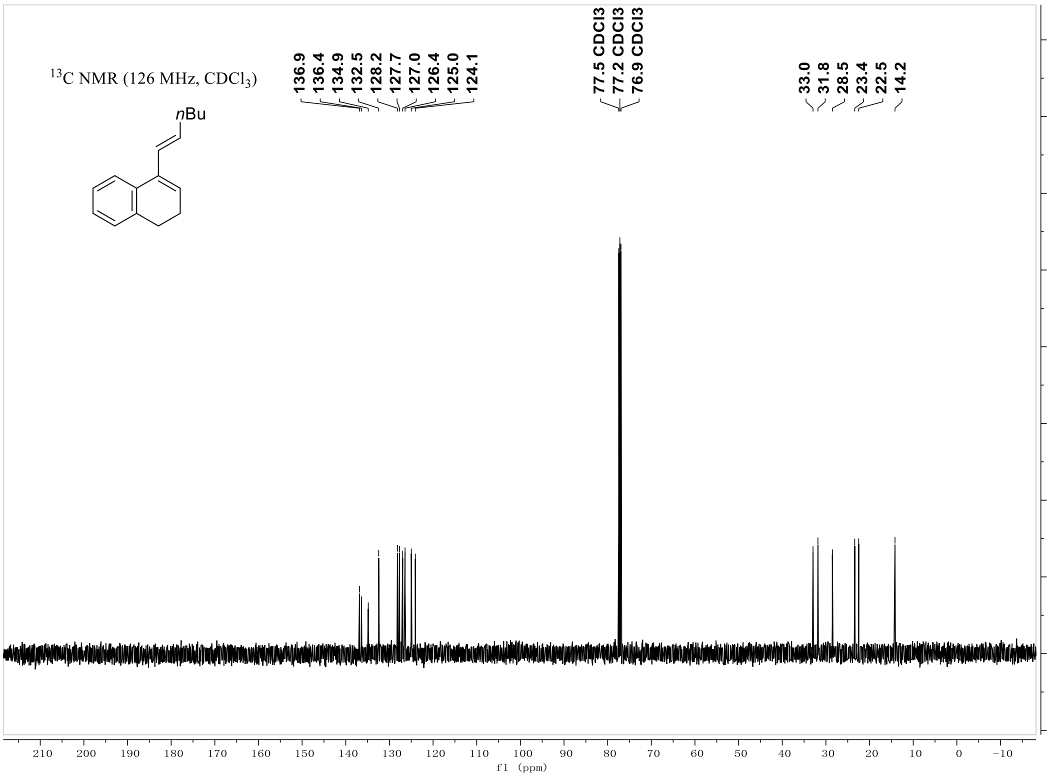

Supplementary Fig. 51. ^{1}H NMR and ^{13}C NMR spectra for compound S11

Supplementary Methods

1. General Information

Ethylene glycol was purchased from TCI. Other solvents and commercially available reagents were used without purification. Flash column chromatography was performed with silica gel (200–300 mesh). Cyclic voltammograms were recorded on a CHI 760E potentiostat. NMR spectra were recorded on Bruker AV-500 instruments. Data were reported as chemical shifts in ppm relative to TMS (0.00 ppm) for ^{1}H and $CDCl_3$ (77.2 ppm) for ^{13}C. The abbreviations used for explaining the multiplicities were as follows: s = singlet, d = doublet, t = triplet, q = quartet, m = multiplet, br = broad. Infrared spectra were recorded on a Nicolet AVATER FTIR330 spectrometer. High resolution mass spectra (ESI) were recorded by the instrumentation center of Department of Chemistry, Xiamen University, on a Micromass QTOF2 Quadruple/Time-of-Flight Tandem mass spectrometer. Reticulated vitreous carbon (100 pores per inch) can be obtained from Goodfellow.

2. Synthesis and Characterization of Substrates

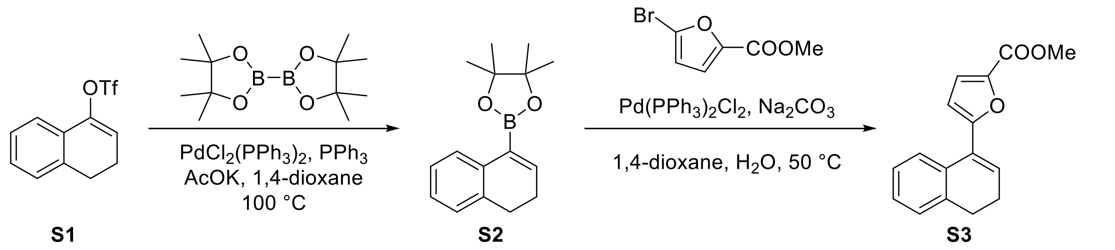

Methyl 5-(3,4-dihydronaphthalen-1-yl)furan-2-carboxylate (S3). A mixed solution of **S1** (1.15 g, 4.10 mmol, 1.00 equiv), bis(pinacolato)diboron (1.14 g, 4.50 mmol, 1.10 equiv), $PdCl_2(PPh_3)_2$ (84 mg, 0.12 mmol, 0.03 equiv), PPh_3 (66 mg, 0.25 mmol, 0.06 equiv), and AcOK (0.60 g, 6.1 mmol, 1.5 equiv) in 1,4-dioxane (30 mL) was heated under stirring at 100 °C for 18 h. The reaction mixture was cooled to rt, the insoluble materials were separated by filtration, and the filtrate was concentrated under reduced pressure. The resulting residue was purified by silica gel column chromatography to afford **S2** (0.89 g, 85% yield), which was directly used without further purification. **S2** (0.31 g, 1.2 mmol, 1.2 equiv), methyl 5-(4,4,5,5-tetramethyl-1,3,2-dioxaborolan-2-yl)furan-2-carboxylate (0.21 g, 1.0 mmol, 1.0 equiv), $Pd(PPh_3)_2Cl_2$ (58 mg, 0.05 mmol, 0.05 equiv) and Na_2CO_3 (0.42 g, 4.0 mmol, 4.0 equiv) were dissolved in a mixture of 1,4-dioxane/H_2O (2.5:1, 14 mL). The resulting mixture was deoxygenated with a stream of argon for 10 min, then heated to 50 °C until complete consumption of the boronic ester (monitored by ^{1}H NMR). The reaction mixture was cooled down to rt and quenched with saturated NH_4Cl (10 mL). The reaction mixture was extracted with ethyl acetate (3 x 15 mL). The combined organic solution was washed with H_2O, saturated NaCl, dried over anhydrous Na_2SO_4, filtered and evaporated under reduced pressure. The residue was purified by silica gel column chromatography to give the desired **S3** as a light yellow oil (0.22 g, 86% yield). ^{1}H NMR (500 MHz, $CDCl_3$) δ 7.44–7.38 (m, 1H), 7.28–7.18 (m, 4H), 6.70 (t, J = 5.0 Hz, 1H), 6.53 (d, J = 3.5 Hz, 1H), 3.90 (s, 3H), 2.78 (t, J = 7.9 Hz, 2H), 2.40 (td, J = 7.9, 5.0 Hz, 2H); ^{13}C NMR (126 MHz, $CDCl_3$) δ 159.4, 156.9, 143.2, 136.9, 132.1, 130.9, 129.1, 128.0, 127.7, 126.7, 124.9, 119.6, 109.5, 52.0, 27.9, 23.3; IR (neat, cm^{-1}): 2942, 1714, 1502, 1307, 1135,

1022, 758; ESI HRMS *m/z* $(M+Na)^+$ calcd 277.0835, obsd 277.0831.

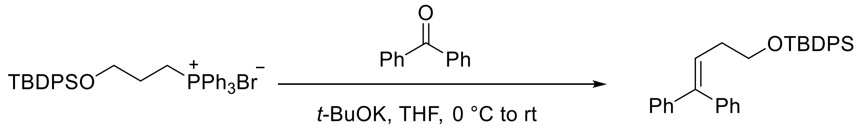

S4 **S5**

***tert*-Butyl((4,4-diphenylbut-3-en-1-yl)oxy)diphenylsilane (S5). S4** (25.53 g, 40 mmol, 1.50 equiv) was suspended in dry THF (200 mL) and cooled to 0 °C. *tert*-BuOK (4.49 g, 40 mmol, 1.50 equiv) was added in one portion and the reaction was stirred at 0 °C for 30 min. Benzophenone (4.87 g, 27.0 mmol, 1.00 equiv) in dry THF (10 mL) was added dropwise and the reaction was warm to RT and stirred overnignt. The reaction was quenched with H_2O and extracted with Et_2O. The combined organic layers were washed with brine and dried over Na_2SO_4, filtered and concentrated under reduced pressure. The crude product was purified by flash column chromatography on silica gel to afford **S5** as a light yellow oil (13.27 g, 94% yield). ^{1}H NMR (500 MHz, $CDCl_3$) δ 7.68–7.61 (m, 4H), 7.42–7.36 (m, 2H), 7.35–7.29 (m, 7H), 7.28–7.22 (m, 2H), 7.22–7.17 (m, 3H), 7.17–7.13 (m, 2H), 6.14 (t, *J* = 7.4 Hz, 1H), 3.75 (t, *J* = 6.4 Hz, 2H), 2.45–2.36 (m, 2H), 1.04 (s, 9H); ^{13}C NMR (126 MHz, $CDCl_3$) δ 143.3, 143.0, 140.2, 135.8, 134.1, 130.1, 129.7, 128.3, 128.2, 127.8, 127.4, 127.1, 127.0, 126.6, 64.0, 33.4, 27.1, 19.4; IR (neat, cm^{-1}): 3070, 2925, 1949, 1591, 1427, 1074, 822, 499; ESI HRMS *m/z* $(M+Na)^+$ calcd 485.2271, obsd 485.2269.

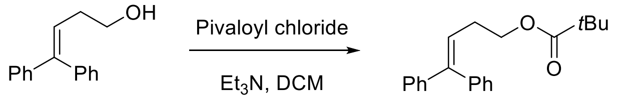

S6 **S7**

4,4-Diphenylbut-3-en-1-yl pivalate (S7). To a solution of **S6** (0.66 g, 3.0 mmol, 1.0 equiv) and Et3N (0.62 mL, 4.5 mmol, 1.5 equiv) in anhydrous CH2Cl2 (15 mL) at 0 °C was added dropwise pivaloyl chloride (0.55 mL, 4.5 mmol, 1.5 equiv). The resulting reaction mixture was warmed to rt and stirred until the consumption of alcohol (monitored by TLC). The solvent was removed under reduced pressure. The residue was suspended in AcOEt (25 mL) and filtered. The filtrate was concentrated under reduced pressure. The residue was purified by flash column chromatography on silica gel to afford **S7** as a white solid (0.80 g, 86% yield). ^{1}H NMR (500 MHz, $CDCl_3$) δ 7.42–7.35 (m, 2H), 7.35–7.29 (m, 1H), 7.29–7.15 (m, 7H), 6.06 (t, *J* = 7.3 Hz, 1H), 4.14 (t, *J* = 6.5 Hz, 2H), 2.53–2.41 (m, 2H), 1.19 (s, 9H); ^{13}C NMR (126 MHz, $CDCl_3$) δ 178.7, 144.2, 142.6, 139.9, 130.0, 128.4, 128.3, 127.5, 127.3, 127.3, 125.0, 64.1, 38.9, 29.6, 27.4; IR (neat, cm^{-1}): 3054, 2962, 1724, 1481, 1286, 1159, 771, 698; ESI HRMS *m/z* $(M+Na)^+$ calcd 331.1669, obsd 331.1662.

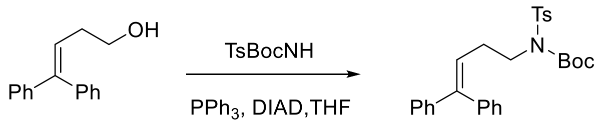

S6 **S8**

***tert*-Butyl (4,4-diphenylbut-3-en-1-yl)(tosyl)carbamate (S8).** Diisopropyl azodicarboxylate (1.37 mL, 7.80 mmol, 1.30 equiv) was added dropwise to a solution of **S6** (1.28 g, 6.00 mmol, 1.00 equiv), N-(*tert*-butoxycarbonyl)-*p*-toluenesulfonamide (2.08 g, 7.80

mmol, 1.30 equiv) and PPh_3 (2.02 g, 7.80 mmol, 1.30 equiv) in THF (30 mL) at 0 °C. The reaction mixture was stirred at rt for 12 h and then concentrated under reduced pressure. The residue was chromatographed through silica gel eluting with EtOAc/hexanes to give the title compound as a light yellow oil (2.55 g, 89% yield). ^{1}H NMR (500 MHz, $CDCl_3$) δ 7.75–7.67 (m, 2H), 7.41–7.34 (m, 2H), 7.34–7.29 (m, 1H), 7.29–7.16 (m, 9H), 6.10 (t, J = 7.5 Hz, 1H), 3.96 (t, J = 7.1 Hz, 2H), 2.59–2.52 (m, 2H), 2.41 (s, 3H), 1.28 (s, 9H); ^{13}C NMR (126 MHz, $CDCl_3$) δ 151.0, 144.2, 144.1, 142.6, 139.9, 137.6, 130.1, 129.3, 128.4, 128.3, 128.1, 127.6, 127.3, 127.3, 125.3, 84.2, 46.8, 30.7, 28.0, 21.8; IR (neat, cm^{-1}): 2977, 1735, 1349, 1141, 766, 675, 544; ESI HRMS m/z (M+Na)$^+$ calcd 500.1866, obsd 500.1878.

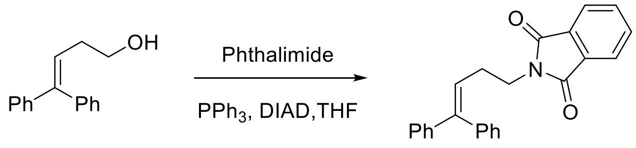

S6 **S9**

2-(4,4-diphenylbut-3-en-1-yl)isoindoline-1,3-dione (S9). Diisopropyl azodicarboxylate (1.19 mL, 6.80 mmol, 1.30 equiv) was added dropwise to a solution of **S6** (1.18 g, 5.31 mmol, 1.00 equiv), phthalimide (1.0 g, 6.8 mmol, 1.3 equiv) and PPh_3 (1.78 g, 6.8 mmol, 1.3 equiv) in THF (30 mL) at 0 °C. The reaction mixture was stirred at rt for 12 h and then concentrated under reduced pressure. The residue was chromatographed through silica gel eluting with EtOAc/hexanes to give the title compound as a white solid (1.18 g, 63% yield). ^{1}H NMR (500 MHz, $CDCl_3$) δ 7.83–7.76 (m, 2H), 7.71–7.65 (m, 2H), 7.30–7.14 (m, 8H), 7.04–6.96 (m, 2H), 6.06 (t, J = 7.6 Hz, 1H), 3.79 (t, J = 6.8 Hz, 2H), 2.61–2.45 (m, 2H); ^{13}C NMR (126 MHz, $CDCl_3$) δ 168.3, 144.6, 142.6, 139.6, 134.0, 132.3, 129.8, 128.4, 128.3, 127.5, 127.3, 125.2, 123.3, 37.8, 29.1; IR (neat, cm^{-1}): 3052, 1774, 1718, 1396, 1025, 721; ESI HRMS m/z (M+Na)$^+$ calcd 376.1308, obsd 376.1308.

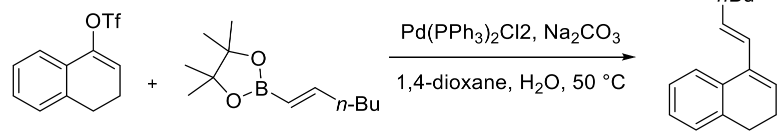

S1 **S10** **S11**

(*E*)-4-(hex-1-en-1-yl)-1,2-dihydronaphthalene (S11). The title compound was prepared from **S10** by following the procedure described for the synthesis of **S3**. Yellow oil; Yield = 99%; ^{1}H NMR (400 MHz, $CDCl_3$) δ 7.37 (d, J = 7.4 Hz, 1H), 7.26–7.15 (m, 3H), 6.35–6.27 (m, 1H), 6.13 (td, J = 4.9, 1.1 Hz, 1H), 6.02 (dt, J = 15.5, 6.9 Hz, 1H), 2.78 (t, J = 7.9 Hz, 2H), 2.36–2.27 (m, 2H), 2.22 (qd, J = 7.1, 1.5 Hz, 2H), 1.53–1.37 (m, 4H), 0.97 (t, J = 7.1 Hz, 3H); ^{13}C NMR (126 MHz, $CDCl_3$) δ 136.9, 136.4, 134.9, 132.5, 128.2, 127.7, 127.0, 126.4, 125.0, 124.1, 33.0, 31.8, 28.5, 23.4, 22.5, 14.2; IR (neat, cm^{-1}): 2941, 1598, 1193, 1070, 789.

3. Characterization Data for the Electrolysis Products

The consumed charge (electricity) F mol^{-1} denotes Faraday (F) per mol of alkene.

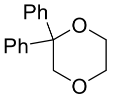

2,2-Diphenyl-1,4-dioxane (4). White solid; Yield = 91%; Electricity = 3.7 F mol^{-1}; ^{1}H NMR (500 MHz, $CDCl_3$) δ 7.43–7.36 (m, 4H), 7.36–7.29 (m, 4H), 7.28–7.22 (m, 2H), 4.12 (s, 2H), 3.79–3.75 (m, 2H), 3.72–3.67 (m, 2H); ^{13}C NMR (126 MHz, $CDCl_3$) δ 143.1, 128.4, 127.4, 78.9, 73.0, 67.2, 61.7; IR (neat, cm^{-1}): 2979, 2896, 2858, 1442, 1079, 897, 698, 607; ESI HRMS *m/z* (M+Na)$^+$ calcd 263.1043, obsd 263.1042.

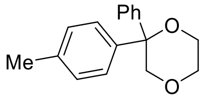

2-Phenyl-2-(*p*-tolyl)-1,4-dioxane (7). White solid; Yield = 72%; Electricity = 4.0 F mol^{-1}; ^{1}H NMR (500 MHz, $CDCl_3$) δ 7.39 (d, J = 7.5 Hz, 2H), 7.35–7.20 (m, 5H), 7.13 (d, J = 7.9 Hz, 2H), 4.12, 4.09 (ABq, 2H, J_{AB} = 15.0 Hz), 3.79–3.72 (m, 2H), 3.72–3.63 (m, 2H), 2.32 (s, 3H); ^{13}C NMR (126 MHz, $CDCl_3$) δ 143.3, 140.1, 137.1, 129.1, 128.4, 127.4, 127.3, 78.9, 73.1, 67.2, 61.7, 21.2; IR (neat, cm^{-1}): 2954, 2852, 1446, 1114, 899, 698, 580; ESI HRMS *m/z* (M+Na)$^+$ calcd 277.1199, obsd 277.1201.

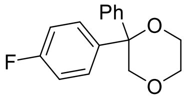

2-(4-Fluorophenyl)-2-phenyl-1,4-dioxane (8). White solid; Yield = 64%; Electricity = 5.4 F mol^{-1}; ^{1}H NMR (500 MHz, $CDCl_3$) δ 7.40–7.29 (m, 6H), 7.28–7.23 (m, 1H), 7.04–6.96 (m, 2H), 4.08 (s, 2H), 3.79–3.74 (m, 2H), 3.70–3.64 (m, 2H); ^{13}C NMR (126 MHz, $CDCl_3$) δ 162.1 (d, $J_{C\text{-}F}$ = 246.2 Hz), 142.9, 138.9 (d, $J_{C\text{-}F}$ = 3.1 Hz), 129.3 (d, $J_{C\text{-}F}$ = 8.0 Hz), 128.4, 127.6, 127.3, 115.2 (d, $J_{C\text{-}F}$ = 21.3 Hz), 78.6, 73.1, 67.2, 61.7; ^{19}F NMR (471 MHz, $CDCl_3$) δ −115.4; IR (neat, cm^{-1}): 2958, 2856, 1510, 1226, 1112, 829, 577; ESI HRMS *m/z* (M+Na)$^+$ calcd 281.0948, obsd 281.0951.

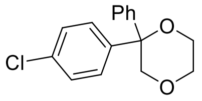

2-(4-Chlorophenyl)-2-phenyl-1,4-dioxane (9). White solid; Yield = 73%; Electricity = 4.7 F mol^{-1}; ^{1}H NMR (500 MHz, $CDCl_3$) δ 7.41–7.21 (m, 9H), 4.08 (s, 2H), 3.79–3.73 (m, 2H), 3.72–3.61 (m, 2H); ^{13}C NMR (126 MHz, $CDCl_3$) δ 142.7, 141.7, 133.4, 129.0, 128.6, 128.5, 127.6, 127.3, 78.6, 72.9, 67.2, 61.7; IR (neat, cm^{-1}): 2958, 2854, 1488, 1110, 899, 700, 611; ESI HRMS *m/z* (M+Na)$^+$ calcd 297.0653, obsd 297.0650.

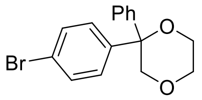

2-(4-Bromophenyl)-2-phenyl-1,4-dioxane (10). White solid; Yield = 56%; Electricity = 8.6 F mol^{-1}; ^{1}H NMR (500 MHz, $CDCl_3$) δ 7.48–7.42 (m, 2H), 7.38–7.30 (m, 4H), 7.30–7.23 (m, 3H), 4.08 (s, 2H), 3.79–3.72 (m, 2H), 3.70–3.64 (m, 2H); ^{13}C NMR (126 MHz, $CDCl_3$) δ 142.6, 142.3, 131.5, 129.4, 128.5, 127.7, 127.3, 121.6, 78.6, 72.8, 67.2, 61.7; IR (neat, cm^{-1}): 3054, 2852, 1486, 1114, 899, 700, 609; ESI HRMS *m/z* (M+Na)$^+$ calcd 341.0148, obsd 341.0150.

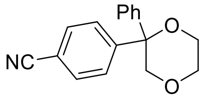

4-(2-Phenyl-1,4-dioxan-2-yl)benzonitrile (11). Light yellow oil; Yield = 62%; Electricity = 4.8 F mol^{-1}; ^{1}H NMR (500 MHz, $CDCl_3$) δ 7.62 (d, J = 8.0 Hz, 2H), 7.52 (d, J = 8.0 Hz, 2H), 7.38–7.31 (m, 4H), 7.31–7.26 (m, 1H), 4.14, 4.07 (ABq, 2H, J_{AB} = 12.4 Hz), 3.81–3.73 (m, 2H), 3.71 (dt, J = 12.3, 4.6 Hz, 1H), 3.67–3.61 (m, 1H); ^{13}C NMR (126 MHz, $CDCl_3$) δ 148.6, 141.8, 132.3, 128.7, 128.2, 127.9, 127.2, 118.9, 111.4, 78.6, 72.5, 67.1, 61.8; IR (neat, cm^{-1}): 2917, 2229, 1602, 1448, 1108, 897, 698, 617; ESI HRMS m/z (M+Na)$^+$ calcd 288.0995, obsd 288.0996.

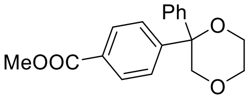

Methyl 4-(2-phenyl-1,4-dioxan-2-yl)benzoate (12). White solid; Yield = 58%; Electricity = 4.8 F mol^{-1}; ^{1}H NMR (500 MHz, $CDCl_3$) δ 8.03–7.95 (m, 2H), 7.52–7.45 (m, 2H), 7.40–7.35 (m, 2H), 7.35–7.30 (m, 2H), 7.29–7.24 (m, 1H), 4.15, 4.11 (ABq, 2H, J_{AB} = 12.5 Hz), 3.90 (s, 3H), 3.81–3.74 (m, 2H), 3.74–3.63 (m, 2H); ^{13}C NMR (126 MHz, $CDCl_3$) δ 167.0, 148.3, 142.4, 129.7, 129.3, 128.5, 127.7, 127.4, 127.3, 78.8, 72.7, 67.2, 61.8, 52.3; IR (neat, cm^{-1}): 3033, 2964, 2852, 1718, 1448, 1274, 1106, 897, 760, 611; ESI HRMS m/z (M+Na)$^+$ calcd 321.1097, obsd 321.1098.

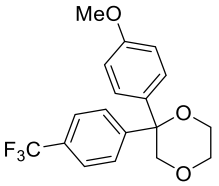

2-(4-Methoxyphenyl)-2-(4-(trifluoromethyl)phenyl)-1,4-dioxane (13). Colorless oil; Yield = 50%; Electricity = 4.2 F mol^{-1}; ^{1}H NMR (500 MHz, $CDCl_3$) δ 7.58 (d, J = 8.3 Hz, 2H), 7.51 (d, J = 8.3 Hz, 2H), 7.29 (d, J = 8.5 Hz, 2H), 6.87 (d, J = 8.5 Hz, 2H), 4.13, 4.03 (ABq, 2H, J_{AB} = 12.4 Hz), 3.83–3.74 (m, 5H), 3.74–3.59 (m, 2H); ^{13}C NMR (126 MHz, $CDCl_3$) δ 159.1, 147.6, 134.4, 129.6 (q, $J_{C\text{-}F}$ = 32.4 Hz), 128.8, 127.7, 125.3 (q, $J_{C\text{-}F}$ = 3.7 Hz), 124.3 (q, $J_{C\text{-}F}$ = 272.0 Hz), 113.9, 78.4, 72.9, 67.2, 61.7, 55.4; ^{19}F NMR (471 MHz, $CDCl_3$) δ −62.5; IR (neat, cm^{-1}): 2966, 2850, 1616, 1510, 1321, 1072, 837, 609; ESI HRMS m/z (M+Na)$^+$ calcd 361.1022, obsd 361.1022.

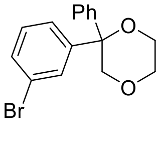

2-(3-Bromophenyl)-2-phenyl-1,4-dioxane (14). Colorless oil; Yield = 61%; Electricity = 4.8 F mol^{-1}; ^{1}H NMR (500 MHz, $CDCl_3$) δ 7.66–7.57 (m, 1H), 7.39–7.37 (m, 3H), 7.33 (t, J = 7.5 Hz, 2H), 7.30–7.23 (m, 2H), 7.20–7.14 (m, 1H), 4.10, 4.06 (ABq, 2H, J_{AB} = 12.4 Hz), 3.81–3.72 (m, 2H), 3.72–3.62 (m, 2H); ^{13}C NMR (126 MHz, $CDCl_3$) δ 145.6, 142.4, 130.6, 130.4, 129.9, 128.5, 127.7, 127.3, 126.2, 122.8, 78.5, 72.8, 67.1, 61.7; IR (neat, cm^{-1}): 2966, 2861, 1564, 1446, 1114, 696, 607; ESI HRMS m/z (M+Na)$^+$ calcd 341.0148, obsd 341.0145.

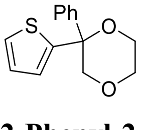

2-Phenyl-2-(thiophen-2-yl)-1,4-dioxane (15). White solid; Yield = 42%; Electricity = 4.2 F

mol^{-1}; ^{1}H NMR (500 MHz, $CDCl_3$) δ 7.48–7.42 (m, 2H), 7.38–7.32 (m, 2H), 7.32–7.26 (m, 2H), 6.99–6.93 (m, 2H), 4.20, 4.05 (ABq, 2H, J_{AB} = 12.2 Hz), 3.90–3.70 (m, 4H); ^{13}C NMR (126 MHz, $CDCl_3$) δ 147.4, 142.6, 128.5, 127.9, 126.7, 126.7, 126.5, 125.9, 77.6, 74.1, 67.1, 61.8; IR (neat, cm^{-1}): 3064, 2956, 2852, 1596, 1446, 1232, 1106, 700; ESI HRMS *m/z* (M+Na)$^+$ calcd 269.0607, obsd 269.0606.

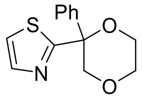

2-(2-Phenyl-1,4-dioxan-2-yl)thiazole (16). Light yellow oil; Yield = 40%; Electricity = 6.8 F mol^{-1}; ^{1}H NMR (500 MHz, $CDCl_3$) δ 7.79 (d, *J* = 3.2 Hz, 1H), 7.50–7.44 (m, 2H), 7.38–7.31 (m, 3H), 7.30–7.25 (m, 1H), 4.59, 4.10 (ABq, 2H, J_{AB} = 12.0 Hz), 3.97 (ddd, *J* = 11.5, 7.5, 3.6 Hz, 1H), 3.89 (dt, *J* = 11.5, 3.8 Hz, 1H), 3.84–3.74 (m, 2H); ^{13}C NMR (126 MHz, $CDCl_3$) δ 173.7, 142.7, 140.9, 128.7, 128.1, 126.0, 120.4, 79.0, 72.8, 66.8, 62.5; IR (neat, cm^{-1}): 3083, 2912, 1450, 1112, 895, 698, 619; ESI HRMS *m/z* (M+Na)$^+$ calcd 270.0559, obsd 270.0551.

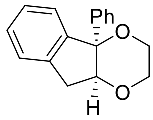

(±)-(4a*R*,9a*S*)-4a-Phenyl-2,3,4a,9a-tetrahydro-9*H*-indeno[1,2-*b*][1,4]dioxine (17). White solid; Yield = 66%; Electricity = 6.0 F mol^{-1}; ^{1}H NMR (500 MHz, $CDCl_3$) δ 7.39–7.31 (m, 4H), 7.30–7.21 (m, 3H), 7.17–7.10 (m, 2H), 4.28 (d, *J* = 3.8 Hz, 1H), 3.96–3.68 (m, 4H), 2.87 (dd, *J* = 16.1, 3.8 Hz, 1H), 2.80 (d, *J* = 16.1 Hz, 1H); ^{13}C NMR (126 MHz, $CDCl_3$) δ 142.6, 142.3, 141.4, 128.9, 128.3, 128.0, 127.5, 127.4, 125.8, 125.1, 87.3, 83.0, 64.9, 61.7, 36.4; IR (neat, cm^{-1}): 3056, 2894, 1598, 1448, 1103, 741, 606, 436; ESI HRMS *m/z* (M+H)$^+$ calcd 275.1043, obsd 275.1040.

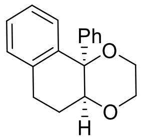

(±)-(4a*S*,10b*R*)-10b-Phenyl-2,3,4a,5,6,10b-hexahydronaphtho[1,2-*b*][1,4]dioxine (18). Colorless oil; Yield = 68%; Electricity = 5.7 F mol^{-1}; ^{1}H NMR (500 MHz, $CDCl_3$) δ 7.42–7.37 (m, 1H), 7.31–7.18 (m, 6H), 7.17–7.12 (m, 2H), 4.01 (td, *J* = 11.5, 3.1 Hz, 1H), 3.90 (dd, *J* = 4.5, 1.6 Hz, 1H), 3.78 (dd, *J* = 11.4, 2.7 Hz, 1H), 3.72 (td, *J* = 11.7, 2.7 Hz, 1H), 3.65 (dd, *J* = 11.9, 3.1 Hz, 1H), 3.12 (ddd, *J* = 18.1, 12.4, 6.6 Hz, 1H), 2.69 (dd, *J* = 18.1, 6.4 Hz, 1H), 1.84 (dt, *J* = 14.2, 5.4 Hz, 1H), 1.77 (tdd, *J* = 14.2, 6.5, 1.6 Hz, 1H); ^{13}C NMR (126 MHz, $CDCl_3$) δ 145.9, 139.1, 136.7, 128.4, 128.3 (2s), 127.8, 127.6 (2s), 126.8, 79.0, 78.0, 67.0, 61.4, 24.7, 24.3; IR (neat, cm^{-1}): 3058, 2948, 2860, 1490, 1448, 1106, 935, 754, 596; ESI HRMS *m/z* (M+Na)$^+$ calcd 289.1199, obsd 289.1200.

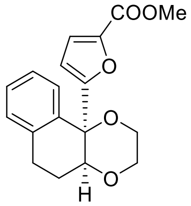

(±)-Methyl 5-((4a*S*,10b*R*)-2,3,5,6-Tetrahydronaphtho[1,2-*b*][1,4]dioxin-10b(4a*H*)-yl)furan-2-carboxylate (19). Light yellow oil; Yield = 69%; Electricity = 5.8 F mol^{-1}; ^{1}H NMR (500 MHz, $CDCl_3$) δ 7.58 (dd, *J* = 7.0, 2.0 Hz, 1H), 7.32–7.23 (m, 2H), 7.21–7.15 (m, 1H), 6.99 (d, *J* = 3.5 Hz, 1H), 5.78 (d, *J* = 3.5 Hz, 1H), 4.41 (d, *J* = 4.2 Hz, 1H), 4.02 (dt, *J* = 11.4, 7.2 Hz, 1H), 3.87 (s, 3H), 3.72 (d, *J* = 11.4 Hz, 1H), 3.67–3.59 (m, 2H), 3.09 (ddd, *J* = 17.1, 12.5, 6.2 Hz, 1H), 2.66 (dd, *J* = 17.1, 6.5 Hz, 1H), 2.07–1.95 (m, 1H), 1.86–1.70 (m, 1H); ^{13}C NMR (126 MHz, $CDCl_3$) δ 160.6, 159.2, 144.5, 138.8, 133.8, 128.7, 128.3, 128.1, 126.5, 118.3, 112.8, 74.3, 74.0, 66.5, 61.1, 52.0, 25.7, 24.1; IR (neat, cm^{-1}): 2933, 2856, 1737, 1589, 1513, 1432, 1299, 1110, 756; ESI HRMS *m/z* $(M+Na)^+$ calcd 337.1046, obsd 2337.1045.

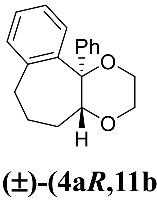

(±)-(4a*R*,11b*R*)-11b-Phenyl-2,3,4a,6,7,11b-hexahydro-5*H*-benzo[3,4]cyclohepta[1,2-*b*][1,4]dioxine (trans-20). The relative configuration was determined using X-ray crystallography experiment. White solid; Yield = 25%; Electricity = 8.2 F mol^{-1}; ^{1}H NMR (500 MHz, $CDCl_3$) δ 8.15 (d, *J* = 7.9 Hz, 1H), 7.54–7.43 (m, 2H), 7.40–7.31 (m, 1H), 7.29–7.20 (m, 4H), 7.01 (d, *J* = 7.3 Hz, 1H), 3.97–3.91 (m, 1H), 3.88–3.76 (m, 3H), 3.63–3.58 (m, 1H), 2.48–2.38 (m, 1H), 2.35 (dd, *J* = 14.0, 6.6 Hz, 1H), 2.16–2.04 (m, 2H), 1.87 (ddt, *J* = 14.7, 7.3, 4.4 Hz, 1H), 1.49–1.36 (m, 1H); ^{13}C NMR (126 MHz, $CDCl_3$) δ 142.7, 141.6, 139.7, 130.6, 130.0, 127.8, 127.8, 127.7, 127.1, 126.2, 81.2, 80.1, 67.5, 59.7, 35.5, 35.2, 26.9.

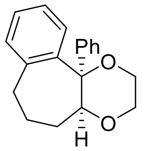

(±)-(4a*S*,11b*R*)-11b-Phenyl-2,3,4a,6,7,11b-hexahydro-5*H*-benzo[3,4]cyclohepta[1,2-*b*][1,4]dioxine (20). Colorless oil; Yield = 39%; Electricity = 8.2 F mol^{-1}; ^{1}H NMR (500 MHz, $CDCl_3$) δ 7.59–7.53 (m, 1H), 7.29–7.19 (m, 8H), 4.24 (t, 1H), 4.08–3.99 (m, 1H), 3.91–3.80 (m, 2H), 3.70–3.59 (m, 1H), 3.17 (dd, *J* = 15.3, 8.9 Hz, 1H), 2.74 (dd, *J* = 15.3, 10.3 Hz, 1H), 1.96–1.85 (m, 2H), 1.69–1.58 (m, 1H), 1.45 (dtt, *J* = 12.5, 6.7, 2.6 Hz, 1H); ^{13}C NMR (126 MHz, $CDCl_3$) δ 143.7, 143.0, 139.3, 131.4, 131.1, 128.1, 128.0, 127.9, 127.7, 126.7, 85.1, 85.0, 68.9, 61.3, 37.6, 31.1, 22.2; IR (neat, cm^{-1}): 3052, 2917, 1444, 1097, 939, 752, 592; ESI HRMS *m/z* $(M+Na)^+$ calcd 303.1356, obsd 303.1356.

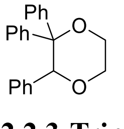

2,2,3-Triphenyl-1,4-dioxane (21). Colorless oil; Yield = 41%; Electricity = 4.8 F mol^{-1}; ^{1}H NMR (500 MHz, $CDCl_3$) δ 7.45 (d, *J* = 7.8 Hz, 2H), 7.41–7.36 (m, 2H), 7.34–7.25 (m, 3H), 7.22–7.16 (m, 2H), 7.15–7.08 (m, 6H), 5.35 (s, 1H), 4.11–3.97 (m, 1H), 3.88–3.74 (m, 3H); ^{13}C NMR (126 MHz, $CDCl_3$) δ 144.1, 142.4, 137.6, 129.3, 128.7, 128.5, 128.1, 127.9, 127.7, 127.5, 127.4, 127.0, 83.4, 82.3, 65.1, 61.4; IR (neat, cm^{-1}): 3062, 2950, 1494, 1106, 752, 702, 609; ESI HRMS *m/z* $(M+Na)^+$ calcd 339.1356, obsd 339.1351.

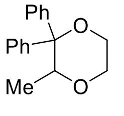

3-Methyl-2,2-diphenyl-1,4-dioxane (22). Colorless oil; Yield = 83%; Electricity = 3.9 F mol^{-1}; ^{1}H NMR (500 MHz, $CDCl_3$) δ 7.45 (d, *J* = 7.6 Hz, 2H), 7.40 (d, *J* = 7.5 Hz, 2H), 7.34–7.18 (m, 6H), 4.40 (q, *J* = 6.7 Hz, 1H), 4.03–3.93 (m, 1H), 3.67–3.54 (m, 3H), 1.19 (d, *J* = 6.7 Hz, 3H); ^{13}C NMR (126 MHz, $CDCl_3$) δ 143.9, 143.2, 128.6, 128.2, 128.0, 127.9, 127.3, 127.0, 81.3, 75.7, 62.8, 61.2, 15.8; IR (neat, cm^{-1}): 3058, 2960, 2871, 1965, 1600, 1446, 1116, 706, 584; ESI HRMS *m/z* $(M+Na)^+$ calcd 277.1199, obsd 277.1197.

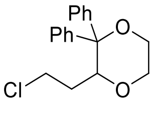

3-(2-Chloroethyl)-2,2-diphenyl-1,4-dioxane (23). White solid; Yield = 78%; Electricity = 4.4 F mol^{-1}; ^{1}H NMR (500 MHz, $CDCl_3$) δ 7.45 (d, *J* = 7.7 Hz, 2H), 7.38–7.31 (m, 3H), 7.31–7.18 (m, 5H), 4.49 (dd, *J* = 10.5, 3.4 Hz, 1H), 3.92–3.83 (m, 1H), 3.69–3.55 (m, 3H), 3.48–3.44 (m, 2H), 2.46 (ddt, *J* = 15.6, 10.5, 5.5 Hz, 1H), 1.51 (dtd, *J* = 15.6, 8.1, 3.4 Hz, 1H); ^{13}C NMR (126 MHz, $CDCl_3$) δ 143.3, 142.9, 128.6, 128.2, 128.2, 127.6, 127.5, 127.2, 80.9, 75.6, 62.0, 61.1, 41.9, 31.0; IR (neat, cm^{-1}): 3060, 2958, 2867, 1446, 1103, 698, 640; ESI HRMS *m/z* $(M+Na)^+$ calcd 325.0966, obsd 325.0967.

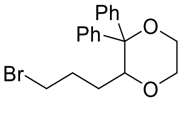

3-(3-Bromopropyl)-2,2-diphenyl-1,4-dioxane (24). White solid; Yield = 53%; Electricity = 4.8 F mol^{-1}; ^{1}H NMR (500 MHz, CDC13) δ 7.44 (d, *J* = 7.7 Hz, 2H), 7.36 (d, *J* = 7.7 Hz, 2H), 7.34–7.18 (m, 6H), 4.22 (dd, *J* = 10.6, 3.5 Hz, 1H), 3.96–3.86 (m, 1H), 3.69–3.52 (m, 3H), 3.34 (dt, *J* = 9.9, 6.4 Hz, 1H), 3.27 (dt, *J* = 9.9, 7.1 Hz, 1H), 2.13 (dtd, *J* = 14.8, 10.1, 4.8 Hz, 1H), 1.95 (tdd, *J* = 14.5, 11.8, 7.3 Hz, 1H), 1.77–1.62 (m, 1H), 1.25–1.14 (m, 1H); ^{13}C NMR (126 MHz, CDC13) δ 143.6, 143.0, 128.6, 128.1, 128.1, 127.8, 127.4, 127.1, 81.2, 78.3, 62.3, 61.1, 33.7, 29.7, 26.6; IR (neat, cm^{-1}): 2956, 1448, 1101, 752.1, 698.1; ESI HRMS *m/z* $(M+Na)^+$ calcd 383.0617, obsd 383.0620.

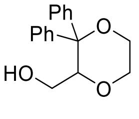

(3,3-Diphenyl-1,4-dioxan-2-yl)methanol (25). Light yellow oil; Yield = 66%; Electricity = 4.7 F mol^{-1}; ^{1}H NMR (500 MHz, $CDCl_3$) δ 7.44 (d, *J* = 7.7 Hz, 2H), 7.38–7.30 (m, 4H), 7.30–7.24 (m, 3H), 7.24–7.19 (m, 1H), 4.46 (dd, *J* = 9.7, 4.1 Hz, 1H), 4.07 (t, *J* = 10.8 Hz, 1H), 4.04–3.95 (m, 1H), 3.73–3.58 (m, 3H), 3.16 (ddd, *J* = 12.1, 8.2, 4.0 Hz, 1H), 1.93–1.78 (m, 1H); ^{13}C NMR (126 MHz, $CDCl_3$) δ 143.1, 142.4, 128.4, 128.4, 128.2, 127.6, 127.4, 127.1, 79.5, 77.9, 61.7, 61.0, 59.1; IR (neat, cm^{-1}): 3463, 2954, 1446, 1118, 698; ESI HRMS *m/z* $(M+Na)^+$ calcd 293.1148, obsd 293.1144.

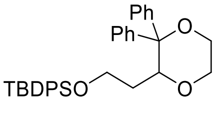

***tert*-Butyl(2-(3,3-diphenyl-1,4-dioxan-2-yl)ethoxy)diphenylsilane (26)**. Light yellow oil; Yield = 59%; Electricity = 3.9 F mol^{-1}; ^{1}H NMR (500 MHz, $CDCl_3$) δ 7.63–7.56 (m, 2H), 7.56–7.49 (m, 2H), 7.46–7.40 (m, 2H), 7.40–7.25 (m, 12H), 7.25–7.18 (m, 2H), 4.48 (dd, *J* = 10.3, 3.1 Hz, 1H), 3.85–3.75 (m, 1H), 3.66 (td, *J* = 9.4, 5.6 Hz, 1H), 3.63–3.49 (m, 4H), 2.13

(ddt, J = 14.9, 10.3, 5.0 Hz, 1H), 1.46 (dddd, J = 14.9, 9.3, 6.8, 3.1 Hz, 1H), 0.99 (s, 9H); ^{13}C NMR (126 MHz, $CDCl_3$) δ 143.8, 143.1, 135.7, 135.7, 134.1, 134.1, 129.6, 128.7, 128.2, 128.1, 128.0, 127.7, 127.7, 127.3, 127.0, 81.5, 76.2, 62.8, 61.2, 60.8, 31.2, 27.0, 19.4; IR (neat, cm^{-1}): 3072, 2923, 1587, 1429, 1259, 1137, 729, 613, 498; ESI HRMS *m/z* $(M+Na)^+$ calcd 545.2482, obsd 545.2484.

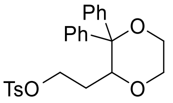

2-(3,3-Diphenyl-1,4-dioxan-2-yl)ethyl 4-methylbenzenesulfonate (27). White solid; Yield = 85%; Electricity = 4.8 F mol^{-1}; ^{1}H NMR (500 MHz, $CDCl_3$) δ 7.72 (d, J = 8.0 Hz, 2H), 7.37 (d, J = 7.7 Hz, 2H), 7.33–7.21 (m, 9H), 7.21–7.16 (m, 1H), 4.30 (dd, J = 10.7, 3.6 Hz, 1H), 4.03–3.93 (m, 2H), 3.77 (ddd, J = 11.5, 7.8, 3.5 Hz, 1H), 3.63–3.51 (m, 2H), 3.51–3.44 (m, 1H), 2.43 (s, 3H), 2.29 (ddt, J = 15.9, 10.7, 5.6 Hz, 1H), 1.44 (dtd, J = 15.9, 7.9, 3.6 Hz, 1H); ^{13}C NMR (126 MHz, $CDCl_3$) δ 144.7, 143.1, 142.6, 133.2, 129.9, 128.5, 128.2, 128.1, 128.0, 127.6, 127.5, 127.2, 80.7, 74.8, 67.8, 61.9, 60.9, 27.5, 21.8; IR (neat, cm^{-1}): 2956, 1600, 1448, 1355, 1176, 1091, 930, 698, 552; ESI HRMS *m/z* $(M+Na)^+$ calcd 461.1393, obsd 461.1400.

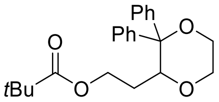

2-(3,3-Diphenyl-1,4-dioxan-2-yl)ethyl pivalate (28). White solid; Yield = 65%; Electricity = 4.4 F mol^{-1}; ^{1}H NMR (500 MHz, $CDCl_3$) δ 7.43 (d, J = 7.7 Hz, 2H), 7.37 (d, J = 7.7 Hz, 2H), 7.34–7.19 (m, 6H), 4.35 (dd, J = 10.7, 3.1 Hz, 1H), 4.10–4.01 (m, 1H), 4.02–3.89 (m, 2H), 3.69–3.53 (m, 3H), 2.28 (ddt, J = 15.6, 10.7, 5.4 Hz, 1H), 1.44 (dddd, J = 15.1, 9.4, 6.8, 3.1 Hz, 1H), 1.13 (s, 9H); ^{13}C NMR (126 MHz, $CDCl_3$) δ 178.5, 143.5, 142.8, 128.6, 128.2, 128.1, 127.9, 127.4, 127.2, 81.1, 76.3, 62.6, 61.5, 61.1, 38.8, 27.4, 27.3; IR (neat, cm^{-1}): 2954, 2865, 1722, 1446, 1280, 1170, 761, 706; ESI HRMS *m/z* $(M+Na)^+$ calcd 391.1880, obsd 391.1874.

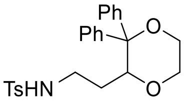

N-(2-(3,3-Diphenyl-1,4-dioxan-2-yl)ethyl)-4-methylbenzenesulfonamide (29). Light yellow oil; Yield = 75%; Electricity = 5.6 F mol^{-1}; ^{1}H NMR (500 MHz, $CDCl_3$) δ 7.69 (d, J = 8.0 Hz, 2H), 7.34 (d, J = 7.0 Hz, 2H), 7.32–7.18 (m, 10H), 4.83 (t, J = 6.2 Hz, 1H), 4.13 (dd, J = 11.0, 3.3 Hz, 1H), 3.89–3.81 (m, 1H), 3.63–3.48 (m, 3H), 3.02–2.84 (m, 2H), 2.43 (s, 3H), 2.15 (ddt, J = 14.4, 11.0, 6.3 Hz, 1H), 1.24 (dtd, J = 14.4, 6.7, 3.3 Hz, 1H); ^{13}C NMR (126 MHz, $CDCl_3$) δ 143.4, 143.3, 142.2, 137.2, 129.8, 128.5, 128.3, 128.1, 128.0, 127.5, 127.3, 127.2, 81.1, 79.0, 63.3, 60.9, 41.4, 28.0, 21.7; IR (neat, cm^{-1}): 3290, 3050, 2968, 1596, 1448, 1322, 1153, 700, 550; ESI HRMS *m/z* $(M+Na)^+$ calcd 460.1553, obsd 460.1548.

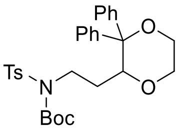

***tert*-Butyl (2-(3,3-diphenyl-1,4-dioxan-2-yl)ethyl)(tosyl)carbamate (30)**. White solid; Yield = 69%; Electricity = 4.0 F mol^{-1}; ^{1}H NMR (500 MHz, $CDCl_3$) δ 7.65 (d, J = 8.1 Hz,

2H), 7.47 (d, *J* = 7.7 Hz, 2H), 7.38–7.18 (m, 10H), 4.43 (dd, *J* = 11.4, 3.2 Hz, 1H), 4.06 (ddd, *J* = 11.9, 8.3, 3.8 Hz, 1H), 3.94 (ddd, *J* = 15.1, 10.6, 5.0 Hz, 1H), 3.78–3.51 (m, 4H), 2.51–2.39 (m, 4H), 1.39 (tdd, *J* = 14.1, 5.1, 3.2 Hz, 1H), 1.26 (s, 9H); ^{13}C NMR (126 MHz, $CDCl_3$) δ 150.9, 144.1, 143.4, 143.4, 137.4, 129.4, 128.6, 128.2, 128.2, 128.0, 127.4, 127.2, 127.0, 84.1, 80.7, 76.2, 61.3, 61.1, 44.9, 28.0, 27.9, 21.8; IR (neat, cm^{-1}): 2968, 1726, 1450, 1349, 1170, 702, 548; ESI HRMS *m/z* (M+Na)$^+$ calcd 560.2077, obsd 560.2087.

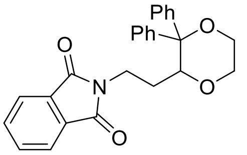

2-(2-(3,3-Diphenyl-1,4-dioxan-2-yl)ethyl)isoindoline-1,3-dione (31). White solid; Yield = 75%; Electricity = 4.8 F mol^{-1}; ^{1}H NMR (500 MHz, CDCl3) δ 7.81–7.74 (m, 2H), 7.71–7.64 (m, 2H), 7.38 (d, *J* = 7.7 Hz, 2H), 7.34 (d, *J* = 7.7 Hz, 2H), 7.28–7.16 (m, 5H), 7.16–7.10 (m, 1H), 4.35 (dd, *J* = 10.3, 3.0 Hz, 1H), 4.02 (ddd, *J* = 11.1, 6.8, 3.7 Hz, 1H), 3.74–3.56 (m, 5H), 2.43–2.29 (m, 1H), 1.54–1.41 (m, 1H); ^{13}C NMR (126 MHz, $CDCl_3$) δ 168.4, 143.4, 142.6, 133.9, 132.3, 128.5, 128.1, 127.9, 127.3, 127.2, 123.2, 81.1, 77.8, 62.7, 61.1, 35.8, 27.3; IR (neat, cm^{-1}): 2921, 1704, 1392, 1141, 920, 700; ESI HRMS *m/z* (M+Na)$^+$ calcd 436.1519, obsd 436.1520.

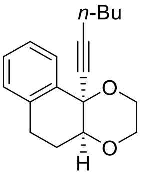

(±)-(4a*S*,10b*R*)-10b-(Hex-1-yn-1-yl)-2,3,4a,5,6,10b-hexahydronaphtho[1,2-*b*][1,4]dioxine (32). MeCN/CH_2Cl_2 (1/2) was used as the solvent. Colorless oil; Yield = 60%; Electricity = 9.1 F mol^{-1}; ^{1}H NMR (500 MHz, $CDCl_3$) δ 7.68–7.62 (m, 1H), 7.28–7.18 (m, 2H), 7.11 (d, *J* = 7.4 Hz, 1H), 4.03 (dd, *J* = 5.1, 1.7 Hz, 1H), 3.82 (td, *J* = 11.2, 2.8 Hz, 1H), 3.66 (ddd, *J* = 11.5, 2.9, 1.5 Hz, 1H), 3.58 (dt, *J* = 11.8, 2.9, 1.5 Hz, 1H), 3.47 (dd, *J* = 11.2, 2.8 Hz, 1H), 3.04 (ddd, *J* = 17.4, 11.9, 5.9 Hz, 1H), 2.67 (ddd, *J* = 16.9, 6.3, 2.3 Hz, 1H), 2.34–2.25 (m, 1H), 2.18 (t, *J* = 7.1 Hz, 2H), 2.12 (dtd, *J* = 13.8, 5.5, 2.1 Hz, 1H), 1.50–1.40 (m, 2H), 1.39–1.28 (m, 2H), 0.86 (t, *J* = 7.2 Hz, 3H); ^{13}C NMR (126 MHz, $CDCl_3$) δ 137.2, 136.2, 128.7, 127.8, 127.5, 126.7, 88.0, 81.8, 76.2, 70.8, 65.9, 60.9, 30.7, 25.9, 24.5, 22.1, 18.7, 13.7; IR (neat, cm^{-1}): 2941, 2235, 1446, 1278, 1089, 928, 754, 606; ESI HRMS *m/z* (M+Na)$^+$ calcd 293.1512, obsd 293.1510.

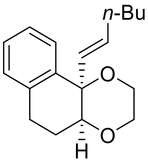

(±)-(4a*S*,10b*R*)-10b-((*E*)-Hex-1-en-1-yl)-2,3,4a,5,6,10b-hexahydronaphtho[1,2-*b*][1,4]dioxine (33). The cis-configuration was determined using NOE experiment. Colorless oil; Yield = 51%; Electricity = 7.5 F mol^{-1}; ^{1}H NMR (400 MHz, $CDCl_3$) δ 7.45–7.38 (m, 1H), 7.25–7.16 (m, 2H), 7.16–7.08 (m, 1H), 5.58 (dt, *J* = 15.5, 1.5 Hz, 1H), 5.20 (dt, *J* = 15.5, 6.8 Hz, 1H), 3.82 (td, *J* = 11.2, 3.4 Hz, 1H), 3.75 (dd, *J* = 4.4, 1.9 Hz, 1H), 3.66 (ddd, *J* = 11.4, 2.7, 0.9 Hz, 1H), 3.61–3.46 (m, 2H), 3.04 (ddd, *J* = 16.7, 12.3, 6.5 Hz, 1H), 2.62 (ddd, *J* =

17.0, 6.5, 1.5 Hz, 1H), 2.07 (dddd, J = 14.3, 12.4, 6.5, 1.9 Hz, 1H), 2.01–1.90 (m, 3H), 1.35–1.15 (m, 4H), 0.84 (t, J = 7.0 Hz, 3H); ^{13}C NMR (126 MHz, $CDCl_3$) δ 138.3, 136.6, 135.0, 134.9, 128.4, 128.2, 127.2, 126.2, 76.6, 76.2, 66.8, 61.2, 32.3, 31.3, 25.5, 24.4, 22.4, 14.1; IR (neat, cm^{-1}): 2958, 2852, 1452, 1114, 972, 764; ESI HRMS *m/z* $(M+Na)^+$ calcd 295.1669, obsd 295.1665.

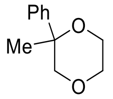

2-Methyl-2-phenyl-1,4-dioxane (34). Light yellow oil; Yield = 19%; electricity = 3.8 F mol^{-1}; ^1H NMR (500 MHz, $CDCl_3$) δ 7.49–7.45 (m, 2H), 7.41–7.35 (m, 2H), 7.30–7.26 (m, 1H), 4.15 (d, J = 11.9 Hz, 1H), 3.79–3.60 (m, 5H), 1.43 (s, 3H); ^{13}C NMR (126 MHz, $CDCl_3$) δ 143.6, 128.6, 127.3, 126.1, 74.6, 73.5, 67.2, 61.6, 26.2; IR (neat, cm^{-1}): 2912, 1130, 953, 700, 602; ESI HRMS *m/z* $(M+Na)^+$ calcd 201.0886, obsd 201.0884.

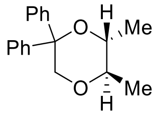

(5*R*,6*R*)-5,6-Dimethyl-2,2-diphenyl-1,4-dioxane (37). White solid; Yield = 72%; Electricity = 4.2 F mol^{-1}; ^1H NMR (400 MHz, $CDCl_3$) δ 7.55–7.47 (m, 2H), 7.40–7.32 (m, 2H), 7.31–7.22 (m, 5H), 7.21–7.14 (m, 1H), 4.63 (d, J = 12.3 Hz, 1H), 3.65 (d, J = 12.3 Hz, 1H), 3.39 (dq, J = 9.0, 6.1 Hz, 1H), 3.31 (dq, J = 9.0, 6.1 Hz, 1H), 1.15 (d, J = 6.1 Hz, 3H), 1.05 (d, J = 6.1 Hz, 3H); ^{13}C NMR (126 MHz, $CDCl_3$) δ 144.6, 142.6, 128.6, 128.4, 128.2, 127.2, 127.1, 125.9, 78.9, 77.8, 72.8, 71.4, 17.7, 17.4; IR (neat, cm^{-1}): 2981, 1600, 1448, 1091, 694, 602, 521; ESI HRMS *m/z* $(M+Na)^+$ calcd 291.1356, obsd 291.1354; $[\alpha]_D^{20}$ = +229.5° (*c* 1.0, $CHCl_3$).

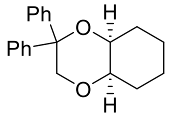

2,2-Diphenyloctahydrobenzo[*b*][1,4]dioxine (38). Colorless oil; Yield = 70%; Electricity = 3.9 F mol^{-1}; ^1H NMR (500 MHz, $CDCl_3$) δ 7.54–7.47 (m, 2H), 7.39–7.33 (m, 2H), 7.33–7.23 (m, 5H), 7.23–7.16 (m, 1H), 4.27, 3.91 (ABq, 2H, J_{AB} = 12.7 Hz), 3.84–3.77 (m, 1H), 3.57 (dt, J = 11.9, 4.1 Hz, 1H), 2.41–2.30 (m, 1H), 1.94–1.81 (m, 2H), 1.73–1.62 (m, 1H), 1.61–1.52 (m, 1H), 1.47 (dt, J = 13.3, 3.6 Hz, 1H), 1.43–1.32 (m, 1H), 1.31–1.17 (m, 1H); ^{13}C NMR (126 MHz, $CDCl_3$) δ 145.4, 142.6, 128.7, 128.4, 128.2, 127.4, 127.2, 126.0, 79.4, 72.7, 67.6, 64.7, 31.1, 24.6, 23.7, 20.6; IR (neat, cm^{-1}): 3058, 2946, 1446, 1376, 1099, 999, 696, 582; ESI HRMS *m/z* $(M+Na)^+$ calcd 317.1512, obsd 317.1508.

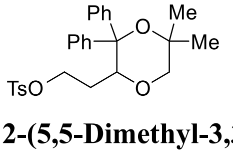

2-(5,5-Dimethyl-3,3-diphenyl-1,4-dioxan-2-yl)ethyl 4-methylbenzenesulfonate (39). 18 mmol of diol was used. The configuration was determined using NOE experiment. Colorless oil; Yield = 52%; Electricity = 10.6 F mol^{-1}; ^1H NMR (500 MHz, $CDCl_3$) δ 7.72 (d, J = 8.0 Hz, 2H), 7.36–7.29 (m, 4H), 7.29–7.14 (m, 8H), 4.21 (dd, J = 10.5, 3.7 Hz, 1H), 3.99–3.90 (m, 2H), 3.52, 3.29 (ABq, 2H, J_{AB} = 11.5 Hz), 2.44 (s, 3H), 2.20 (ddt, J = 15.8, 10.5, 5.6 Hz, 1H), 1.43 (dtd, J = 15.8, 7.8, 3.7 Hz, 1H), 0.97 (s, 3H), 0.85 (s, 3H); ^{13}C NMR (126 MHz, $CDCl_3$) δ 145.8, 144.8, 144.7, 133.3, 129.9, 128.6, 128.1, 128.0, 127.9, 127.8, 127.3, 127.0,

78.6, 75.1, 71.3, 71.1, 67.9, 28.2, 27.2, 27.2, 21.8; IR (neat, cm^{-1}): 3062, 2983, 1596, 1392, 1087, 671, 561; ESI HRMS *m/z* $(M+Na)^+$ calcd 489.1706, obsd 489.1711.

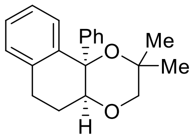

(±)-(4a*s*,10b*R*)-2,2-Dimethyl-10b-phenyl-2,3,4a,5,6,10b-hexahydronaphtho[1,2-*b*][1,4]dioxine (40). 18 mmol of diol was used. The reaction was conducted in MeCN/CH_2Cl_2 (1/2). The configuration was determined using NOE experiment. White solid; Yield = 45%; Electricity = 6.4 F mol^{-1}; ^1H NMR (500 MHz, $CDCl_3$) δ 7.37 (d, *J* = 7.8 Hz, 1H), 7.27–7.10 (m, 8H), 3.68 (dd, *J* = 4.3, 1.8 Hz, 1H), 3.65 (s, 2H), 3.22 (ddd, *J* = 17.9, 12.6, 6.1 Hz, 1H), 2.66 (dd, *J* = 16.8, 5.5 Hz, 1H), 1.97–1.82 (m, 2H), 1.26 (s, 3H), 0.79 (s, 3H); ^{13}C NMR (126 MHz, $CDCl_3$) δ 147.7, 140.1, 137.2, 130.6, 128.5, 127.9, 127.6, 127.2, 127.2, 125.9, 78.8, 76.8, 75.7, 71.1, 27.4, 25.5, 25.0, 24.3; IR (neat, cm^{-1}): 3054, 2973, 1452, 1170, 1099, 750, 559; ESI HRMS *m/z* $(M+H)^+$ calcd 295.1693, obsd 295.1699.

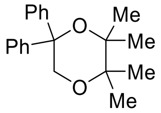

2,2,3,3-Tetramethyl-5,5-diphenyl-1,4-dioxane (41). A modified workup procedure was adapted to remove a ketone side product. The crude mixture was dissolved in 5 mL of MeOH and cooled to 0 °C. 5 mg of $NaBH_4$ was added and the resulting mixture was stirred at rt for 30 min. 1 mL of water was added and the resulting mixture was extracted with EtOAc (3 x 20 mL). The combined organic solution was dried over anhydrous $MgSO_4$ and concentrated under reduced pressure. The residue was chromatographed through silica gel eluting with ethyl acetate/hexanes to give the title compound. Colorless oil; Yield = 47%; Electricity = 5.5 F mol^{-1}; ^1H NMR (500 MHz, $CDCl_3$) δ 7.40–7.35 (m, 4H), 7.30–7.24 (m, 4H), 7.22–7.17 (m, 2H), 4.16 (s, 2H), 1.25 (s, 6H), 0.95 (s, 6H); ^{13}C NMR (126 MHz, CDCl3) δ 146.7, 128.0, 127.2, 126.9, 76.8, 76.0, 75.2, 25.6, 23.2; IR (neat, cm^{-1}): 2975, 1594, 1448, 1124, 987, 696, 602; ESI HRMS *m/z* $(M+Na)^+$ calcd 319.1669, obsd 319.1667.

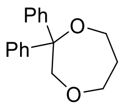

2,2-Diphenyl-1,4-dioxepane (42). Light yellow oil; Yield =55%; Electricity = 4.0 F mol^{-1}; ^1H NMR (500 MHz, $CDCl_3$) δ 7.43–7.35 (m, 4H), 7.34–7.27 (m, 4H), 7.26–7.20 (m, 2H), 4.25 (s, 2H), 3.91–3.79 (m, 4H), 2.13–2.02 (m, 2H); ^{13}C NMR (126 MHz, $CDCl_3$) δ 144.1, 128.3, 127.4, 127.2, 84.2, 78.3, 70.5, 63.5, 33.0; IR (neat, cm^{-1}): 3058, 2939, 2863, 1596, 1446, 1128, 696, 590; ESI HRMS *m/z* $(M+Na)^+$ calcd 277.1199, obsd 277.1196.

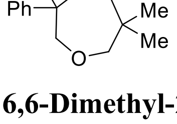

6,6-Dimethyl-2,2-diphenyl-1,4-dioxepane (43). White solid; Yield = 63%; Electricity = 3.9 F mol^{-1}; ^1H NMR (500 MHz, $CDCl_3$) δ 7.42–7.35 (m, 4H), 7.34–7.27 (m, 4H), 7.26–7.20 (m, 2H), 4.32 (s, 2H), 3.58–3.46 (m, 4H), 0.96 (s, 6H); ^{13}C NMR (126 MHz, CDCl3) δ 144.9, 128.3, 127.3, 127.0, 84.7, 84.1, 80.4, 73.8, 38.7, 23.7; IR (neat, cm^{-1}): 3061, 2953, 2872, 1599,

1446, 1223, 1128, 700; ESI HRMS *m/z* $(M+Na)^+$ calcd 305.1512, obsd 305.1510.

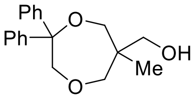

(6-Methyl-2,2-diphenyl-1,4-dioxepan-6-yl)methanol (44). Colorless oil; Yield = 36%; Electricity = 8.9 F mol^{-1}; ^{1}H NMR (500 MHz, $CDCl_3$) δ 7.39–7.31 (m, 6H), 7.31–7.24 (m, 3H), 7.23–7.18 (m, 1H), 4.43, 4.24 (ABq, 2H, J_{AB} = 13.5 Hz), 3.88, 3.45 (ABq, 2H, J_{AB} = 12.4 Hz), 3.72, 3.61 (ABq, 2H, J_{AB} = 13.0 Hz), 3.69, 3.57 (ABq, 2H, J_{AB} = 10.8 Hz), 2.10 (brs, 1H), 0.81 (s, 3H); ^{13}C NMR (126 MHz, $CDCl_3$) δ 144.6, 143.7, 128.5, 128.3, 127.5, 127.3, 127.1, 126.9, 84.5, 80.3, 80.2, 70.8, 68.4, 43.6, 18.5; IR (neat, cm^{-1}): 3442, 2919, 1444, 1130, 698; ESI HRMS *m/z* $(M+Na)^+$ calcd 321.1461, obsd 321.1456.

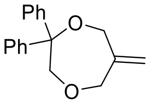

6-Methylene-2,2-diphenyl-1,4-dioxepane (45). A modified workup procedure was adapted to remove a ketone side product. The crude mixture was dissolved in 5 mL of MeOH and cooled to 0 °C. 5 mg of $NaBH_4$ was added and the resulting mixture was stirred at rt for 30 min. 1 mL of water was added and the resulting mixture was extracted with EtOAc (3 x 20 mL). The combined organic solution was dried over anhydrous $MgSO_4$ and concentrated under reduced pressure. The residue was chromatographed through silica gel eluting with ethyl acetate/hexanes to give the title compound. Colorless oil; Yield = 55%; Electricity = 6.4 F mol^{-1}; ^{1}H NMR (500 MHz, $CDCl_3$) δ 7.41–7.35 (m, 4H), 7.34–7.28 (m, 4H), 7.27–7.22 (m, 2H), 5.09 (s, 1H), 4.93 (s, 1H), 4.42–4.39 (m, 2H), 4.18 (s, 2H), 4.15 (s, 2H); ^{13}C NMR (126 MHz, $CDCl_3$) δ 148.6, 143.1, 128.3, 127.9, 127.4, 111.9, 84.7, 79.3, 74.5, 67.8; IR (neat, cm^{-1}): 3054, 2931, 1454, 1446, 1122, 908, 698, 590; ESI HRMS *m/z* $(M+Na)^+$ calcd 289.1199, obsd 289.1195.
